# AURORA A interacts with DICER and SETD2 to promote S-phase progression

**DOI:** 10.1038/s44319-026-00850-0

**Published:** 2026-06-30

**Authors:** Juliane Müller, Tina Daunke, Nicola Berner, Pranjali Bhandare, Anneli Gebhardt, Lasse Hoffmann, Mathias Diebold, Markus Vogt, Julia Hofstetter, Yiliam Cruz-Garcia, Nevenka Dudvarski-Stankovic, Katharina Schneider, Bikash Adhikari, Stephanie Lamer, Andreas Schlosser, Gabriele Büchel, Martin L Sos, Bernhard Küster, Stefan Knapp, Stephanie Wilhelm, Elmar Wolf

**Affiliations:** 1 Institute of Biochemistry, University of Kiel, 24118 Kiel, Germany; 2 Theodor Boveri Institute, Department of Biochemistry and Molecular Biology, Biocenter, University of Würzburg, 97074 Würzburg, Germany; 3 TUM School of Life Science, Technische Universität München, 85354 Freising, Germany; 4 Institute for Pharmaceutical Chemistry, Goethe-University Frankfurt, 60438 Frankfurt am Main, Germany; 5 Structural Genomics Consortium (SGC), Buchmann Institute for Life Sciences (BMLS), Goethe-University Frankfurt, 60438 Frankfurt am Main, Germany; 6 German Cancer Consortium (DKTK), partner site Munich, a partnership between DKFZ and Ludwig-Maximilians-Universität Munich, Department of Translational Oncology, German Cancer Research Center (DKFZ) Heidelberg, 69120 Heidelberg, Germany; 7 Rudolf-Virchow-Zentrum - Center for Integrative and Translational Bioimaging, University of Würzburg, Würzburg, 97080 Germany; 8 Department of Medicine III, LMU University Hospital, 81377 Munich, Germany; 9 Research Unit for Translational Proteolysis Research, Institute of Experimental Medicine, University Hospital Schleswig-Holstein, 24105 Kiel, Germany

**Keywords:** Cell Cycle, DNA Replication, Recombination & Repair, RNA Biology

## Abstract

The oncogenic kinase AURORA A is essential for mitotic progression, and its catalytic inhibition arrests cells at the G2/M-transition. Unexpectedly, degradation of AURORA A by PROTACs (proteolysis targeting chimeras) induces profound S-phase defects, revealing a non-catalytic scaffolding function of AURORA A. To dissect this function, we profile the AURORA A S-phase interactome and identify multiple RNA-binding proteins not characterized as AURORA A substrates. Among these, the ribonuclease DICER directly associates with AURORA A to form an abundant nuclear complex. RNA degradation shifts AURORA A, DICER, and additional RNA-binding proteins from heavy to light gradient fractions, implicating RNA-dependent complex function. In contrast, PROTAC-mediated depletion of AURORA A alters the gradient migration behavior and chromatin association of the histone methyltransferase SETD2, which is known to prevent spurious transcription. These findings reveal a dual-output model for the S-phase AURORA A complex: First, RNA-binding proteins are recruited to R-loops, which may arise from transcription-replication conflicts. DICER then processes the R-loop, while AURORA A simultaneously recruits SETD2, which facilitates efficient resolution of replicative stress by preventing spurious transcription.

## Synopsis

AURORA A has a non-catalytic scaffolding function during S phase, forming an RNA-dependent complex with DICER and other RNA-binding proteins to promote R-loop resolution. AURORA A also recruits SETD2, which suppresses spurious transcription and facilitates the coordination of transcription and DNA replication.

- AURORA A associates with numerous proteins during S phase, forming an RNase-sensitive multiprotein complex.
- Within this complex, AURORA A directly interacts with DICER, and both proteins co-localize in S-phase nuclei.
- The S-phase AURORA A complex also includes the histone methyltransferase SETD2.
- Chromatin association of SETD2 is dependent on AURORA A.

## Abstract (web summary)

AURORA A has a non-catalytic scaffolding function during S phase, forming an RNA-dependent complex with DICER and other RNA-binding proteins to promote R-loop resolution. AURORA A also recruits SETD2, which suppresses spurious transcription and facilitates the coordination of transcription and DNA replication.

## Graphical abstract

**Figure:**
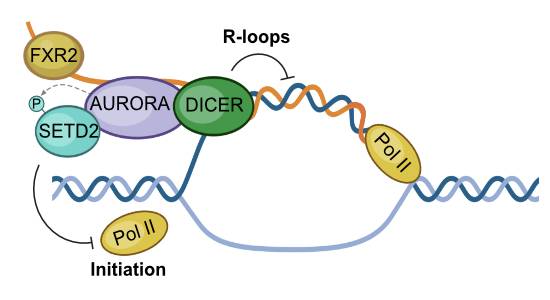

**Figure:**
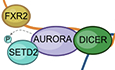

## Introduction

The three members of the AURORA kinase family, AURORA A, B and C, play essential roles in regulating mitosis in mammalian cells (Hochegger et al, 2013; Vats et al, 2025). AURORA A is of particular interest, because its gene is located in a genomic region that is frequently amplified in various human cancers (Bischoff et al, 1998; Tanner et al, 2000), and its overexpression has been shown to induce tumor formation in mouse models (Treekitkarnmongkol et al, 2016; Wang et al, 2006). Moreover, the depletion or inactivation of AURORA A can lead to synthetic lethality when combined with the loss of tumor suppressor genes or oncogene activation (Gong et al, 2019; Mollaoglu et al, 2017; Wu et al, 2018), defining it as a priority target for tumor therapy. Thus, various therapeutic strategies targeting the oncogenic function of AURORA A have been developed and are tested in clinical trials (Pradhan et al, 2021).

The oncogenic function of AURORA A is particularly well-characterized in the context of neuroblastoma, where it forms stable protein complexes with N-MYC (Richards et al, 2016). N-MYC, the neuronal counterpart of the hallmark oncogene c-MYC, is commonly amplified in neuroblastoma and strongly associated with high-risk disease progression (Bottomley et al, 2022). Neuroblastoma cells harboring N-MYC amplification exhibit a particularly pronounced sensitivity to AURORA A depletion (Otto et al, 2009). Interestingly, the AURORA A/N-MYC complex appears to fulfill a function distinct from the canonical roles typically attributed to mitotic AURORA kinases: Despite AURORA A expression peaks during mitosis, the AURORA A/N-MYC complex does not assemble at this stage. Instead, it is predominantly present in replicating cells during S-phase (Buchel et al, 2017). In line with this observation, conformational inhibitors of the AURORA A/N-MYC complex, such as Alisertib (Brockmann et al, 2013) and CD532 (Gustafson et al, 2014), induce S-phase defects in neuroblastoma cells. These defects include cell cycle arrest in S-phase and reduced BrdU incorporation during DNA replication. Interestingly, N-MYC is not a phosphorylation target of AURORA A (Kettenbach et al, 2011), and its complex formation occurs independently of its kinase activity (Richards et al, 2016). Taken together, the characterization of the AURORA A/N-MYC complex suggests that AURORA A mediates critical non-catalytic functions during S-phase in neuroblastoma cells.

To target the scaffolding functions of AURORA A, we and others have developed potent and selective PROTACs (proteolysis targeting chimeras) against AURORA A in recent years (Adhikari et al, 2020; Bozilovic et al, 2022; Chung et al, 2025; Liu et al, 2022; Nelson et al, 2025; Pogash and Fletcher, 2025; Rishfi et al, 2023; Sflakidou et al, 2024; Tang et al, 2025; Wang et al, 2021). PROTACs are small bifunctional molecules that recruit E3 ubiquitin ligases to specific cellular target proteins, thereby inducing their ubiquitination and subsequent proteasomal degradation (Bekes et al, 2022). As expected, PROTAC-mediated degradation of AURORA A in neuroblastoma cells phenocopies the effect of conformational AURORA A/N-MYC inhibitors (Tang et al, 2025).

Surprisingly, however, PROTAC-induced degradation of AURORA A–unlike its catalytic inhibition–also results in a pronounced S-phase arrest in cells that do not express N-MYC (Adhikari et al, 2020; Wang et al, 2021). This suggests that AURORA A exerts non-catalytic functions during S-phase even in the absence of N-MYC, hence in the majority of cancer cells. In contrast to neuroblastoma cells, however, it remains unclear what scaffolding roles AURORA A might fulfill in these contexts, and which protein networks it engages during S-phase in cells not expressing N-MYC.

The aim of this study was to investigate the non-catalytic functions of AURORA A during S-phase in non-neuroblastoma cells. Using a combination of biochemical assays and proteomics, we identified an extensive network of AURORA A-interacting proteins that assembled during S-phase in acute myeloid leukemia (AML) cells. The S-phase interactome of AURORA A was notably enriched in RNA-binding proteins. Using gradient centrifugation, we demonstrated that the ribonuclease DICER and the histone methyltransferase SETD2 required RNA to be integrated into these complexes. PROTAC-induced degradation of AURORA A disrupted this association, leading to the dissociation of SETD2 from chromatin, resulting in S-phase defects.

## Results

### AURORA A interacts with RNA-binding proteins during S-phase

**Figure 1:**
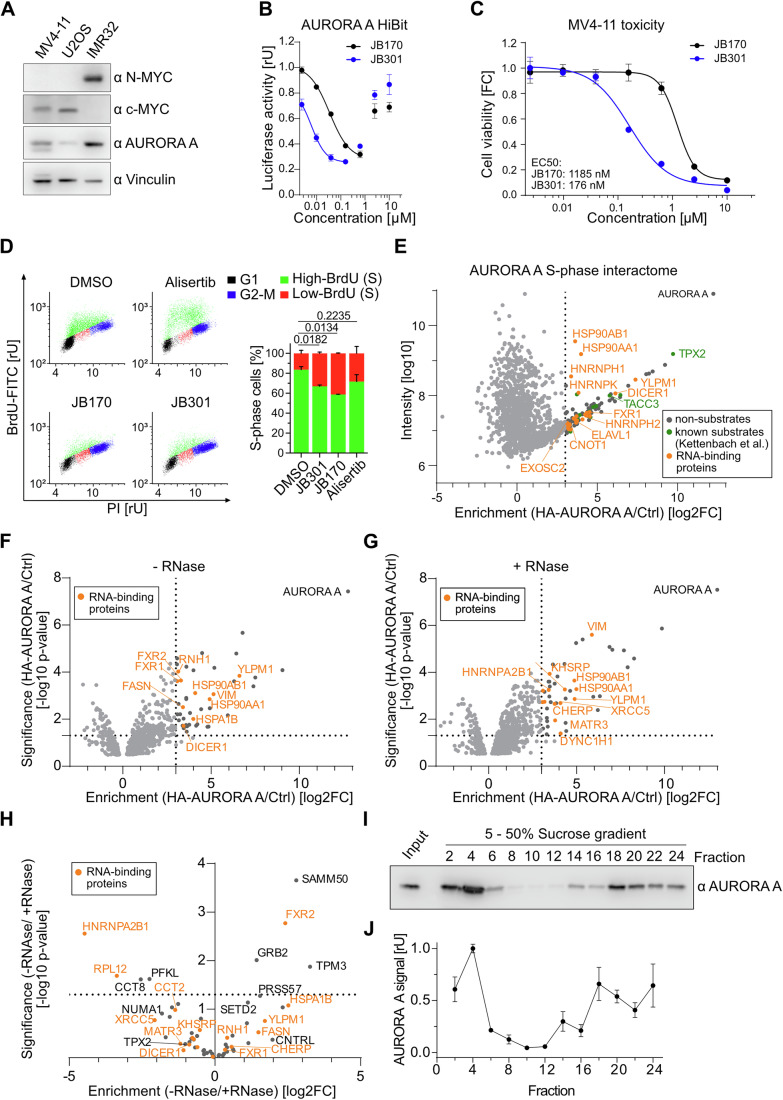
AURORA A interacts with RNA-binding proteins during S-phase. (**A**) Immunoblots showing N-MYC, c-MYC, and AURORA A expression levels in AML MV4-11 cells, osteosarcoma U2OS cells and neuroblastoma IMR32 cells. One representative loading control (Vinculin) is shown. (**B**) AURORA A levels after 6 h of PROTAC treatment based on luciferase measurements. MV4-11 AURORA A-HiBiT cells were treated with different concentrations of JB170 and JB301 for 6 h. Cells were lysed and complemented with the second luciferase fragment and the substrate. Luminescence was measured and normalized to DMSO-treated cells. Each point represents the mean ± SD of three technical replicates. (**C**) PROTAC effect on cell viability. MV4-11 cells were treated with various PROTAC concentrations for 72 h. Cell viability was measured using an alamarBlue assay and compared to DMSO-treated cells. Each point represents the mean ± SD from three technical replicates and was normalized to DMSO-treated cells. EC50: half maximal effective concentration. (**D**) Cell cycle distribution analyzed by flow cytometry. Unsynchronized MV4-11 cells were treated with the AURORA A inhibitor Alisertib (0.5 µM) or PROTACs (JB170: 0.5 µM or JB301: 0.2 µM) for 16 h. Cells were labeled with BrdU and stained with PI followed by analysis with flow cytometry. The percentage of cells in S-phase (High-BrdU: green, Low-BrdU: red) was determined in biological triplicate experiments (*p < 0.05, unpaired two-tailed Welch’s *t*-test). The gating strategy is depicted in Fig. EV1B, and all cell cycle phases are quantified in Fig. EV1C. (**E**) Enrichment plot for AURORA A interactors in S-phase synchronized MV4-11 cells. Enrichment of proteins after exogenous HA-AURORA A IP is shown and compared to cells not expressing HA-AURORA A. The experiment was performed in unicates (1x N-tag HA-AURORA A, 1x C-tag HA-AURORA A) and analyzed by mass spectrometry. (**F**) Volcano plot for AURORA A interactors in MV4-11 cells. The experiment was performed in biological duplicates (2x N-tag HA-AURORA A, 2x C-tag HA-AURORA A) and *p* values were calculated using a two-sample unpaired *t*-test. AURORA A substrates (Kettenbach et al, 2011) and RNA-binding proteins (mammalian RNA-binding protein resource, proteins are labeled as RNA-binding if they were found in at least 5 out of 18 studies (Caudron-Herger et al, 2019)) are colored. Dotted lines indicate the cut-offs for significantly enriched proteins (log2FC >3, *p* value <0.05). (**G**) HA-AURORA A IP was conducted as in (**F**) but with the addition of a RNase mixture. (**H**) Volcano plot depicting the differences in − and + RNase IP conditions for AURORA A interactors. The difference in enrichment is plotted versus the significance of this difference. The *p* values were calculated by a two-sample unpaired *t*-test. RNA-binding proteins are labeled in orange. (**I**) Immunoblot of gradient fractions. MV4-11 HA-CRBN cell lysate was loaded on a 5–50% sucrose gradient, centrifuged and fractionated. The precipitated fractions were loaded on an immunoblot. (**J**) Quantification of immunoblot in (**I**) and two additional biological replicates. The AURORA A signal was normalized to the input, and the highest signal was set to 1.0. Each point represents the mean ± SEM. Source data are available online for this figure.

**Figure EV1:**
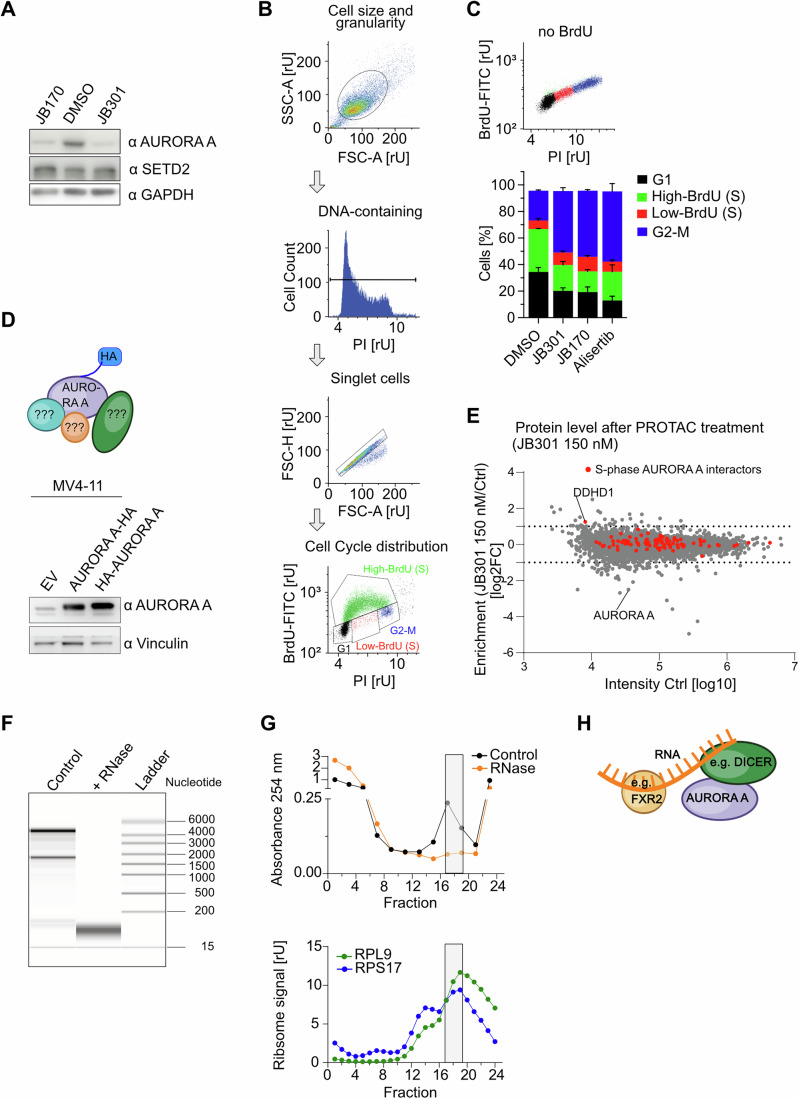
AURORA A interacts with RNA-binding proteins during S-phase. (**A**) Immunoblots showing AURORA A and SETD2 levels after AURORA A degradation with the PROTACs JB170 and JB301. MV4-11 cells were treated for 6 h with either JB170 (600 nM) or JB301 (150 nM), and cell lysates were blotted. (**B**) Flow cytometry gating strategy for BrdU-PI-based cell cycle analysis. Representative gating from an exemplary experiment is shown, illustrating the sequential selection of cells used to determine cell cycle distribution. A similar gating strategy was used for all the BrdU-PI flow cytometry experiments. (**C**) Top: BrdU-PI cell cycle profile of unsynchronized MV4-11 cells without BrdU incorporation, serving as a control for Low-BrdU S-phase cells in Fig. 1D. Bottom: Quantification of BrdU-PI cell cycle distribution from three independent biological replicates. One representative replicate is displayed in Fig. 1D. (**D**) Top: Model of AURORA A and potential interactors. Below: Immunoblots showing AURORA A expression levels in MV4-11 empty vector (EV) and HA-tagged AURORA A overexpressing cells (N- and C-tag). (**E**) Enrichment plot after AURORA A PROTAC treatment. MV4-11 cells overexpressing HA-CRBN were treated with 150 nM JB301, followed by cell lysis and label-free quantification by mass spectroscopy. S-phase AURORA A interactors (Fig. 1E) are labeled in red. (**F**) RNA profile in MV4-11 HA-CRBN cell lysates treated with or without an RNase cocktail (RNase A, RNase III, RNase H, RNase I, and RNase T1). RNA was extracted from cell lysates using Trizol and loaded on a fragment analyzer. (**G**) Top: UV trace of gradient fractions. MV4-11 HA-CRBN cells were lysed and treated with or without an RNase mixture. The lysate was loaded on a 5–50% sucrose gradient, centrifuged and fractionated. Absorbance at 245 nm was measured for all precipitated fractions. Below: Gradient fractions were analyzed by label-free mass spectroscopy and normalized LFQ intensities for ribosome subunits RPL9 and RPS17 are shown. (**H**) Model: AURORA A together with direct and indirect RNA interactors.

To investigate the scaffolding functions of AURORA A in non-neuroblastoma cells, we selected the human AML cell line MV4-11, which does not express N-MYC (Fig. 1A). Notably, these cells exhibit significant sensitivity to AURORA A degradation induced by the PROTACs JB170 (Adhikari et al, 2020) or JB301 (Bozilovic et al, 2022), as evident by the pronounced growth inhibition following PROTAC treatment for 3 days (Figs. 1B,C and EV1A). Moreover, similar to neuroblastoma cells and consistent with previous observations (Adhikari et al, 2020), MV4-11 cells undergo S-phase arrest upon AURORA A degradation, whereas catalytic inhibition of AURORA A resulted in a G2/M arrest. This was demonstrated by flow cytometry analysis following assessing DNA content via propidium iodide (PI) staining and BrdU incorporation (Figs. 1D and EV1B,C).

To better understand this S-phase phenotype, we aimed to identify AURORA A binding partners in S-phase cells. We generated MV4-11 cells stably expressing HA-tagged AURORA A via lentiviral transduction and subjected them to a double thymidine block to enrich for cells in S-phase, followed by native immunoprecipitation of AURORA A complexes using HA-specific antibodies (Fig. EV1D). We then identified AURORA A-interacting proteins by label-free quantitative mass spectrometry, comparing protein enrichment in HA immunoprecipitants from HA-AURORA A-expressing cells to cells expressing only untagged AURORA A (Fig. 1E). As anticipated, AURORA A (log₂FC = 12.2) and its well-characterized binding partner TPX2 (log₂FC = 9.7) showed the highest fold enrichment, confirming the specificity of the pulldown. Among the 116 AURORA A interaction partners (log₂FC >3; Dataset EV1), only 11 were described previously as substrates of AURORA A kinase activity in systematic studies (Kettenbach et al, 2011; Müller et al, 2025), suggesting that either the AURORA A’s substrate spectrum were cell cycle-specific or that S-phase AURORA A engaged in extensive interactions with non-substrate proteins. Short-term degradation of AURORA A by JB301 showed no destabilization of any of the interactors, indicating that PROTAC treatment did not lead to cross-ubiquitylation within the AURORA A interactome (Fig. EV1E). Interestingly, among the 105 non-substrate interactors, 28% (29 proteins) were annotated as RNA-binding proteins. These include YLPM1, which is implicated in telomerase regulation and mutated in gastrointestinal tumors (Xie et al, 2024), DICER, a ribonuclease involved in micro-RNA/siRNA processing (Vergani-Junior et al, 2021) and DNA damage repair (Alagia et al, 2025; Burger et al, 2017; Camino et al, 2023; Cheng et al, 2025), as well as different heterogenous ribonucleoproteins (Figs. 1E; Dataset EV1).

The high abundance of RNA-binding proteins in the S-phase interactome of AURORA A raised the question of whether these interactions occurred via RNA or through direct protein–protein contact with AURORA A. To address this question, we again analyzed AURORA A binding partners by mass spectrometry using samples that were treated with various RNases and compared these interactomes to those containing intact RNA. Gel electrophoresis confirmed the presence of intact RNA in control samples, while RNA was degraded in RNase-treated lysates (Fig. EV1F). To directly assess the influence of RNA on the AURORA A interactome, we conducted both experimental conditions in parallel. Each condition had two biological replicates expressing C-terminally HA-tagged AURORA A and two expressing N-terminally HA-tagged AURORA A. Volcano plot analyses demonstrated that in the presence of intact RNA, 10 RNA-binding proteins (23% of all proteins; Figs. 1F; Dataset EV2) were enriched in the AURORA A interactome, while RNA degradation did not overall reduce the number of interacting RNA-binding proteins (14 proteins, 30%; Fig. 1G). A direct comparison of protein enrichment under both conditions revealed that a few RNA-binding proteins, such as the heterogenous ribonucleoprotein 2B1 (HNRNPA2B1), that regulates mRNA stability and miRNA processing (Wu et al, 2023), exhibited increased interaction upon RNA degradation, while others, including the Fragile X mental retardation syndrome-related protein 2 (FXR2) lost their association with AURORA A (Fig. 1H). Notably, the majority of RNA-binding proteins remained part of the AURORA A interactome irrespective of the RNA status, among them DICER, YLPM1, XRCC5 (involved in DNA repair) (Taccioli et al, 1994) and MATR3 (involved in chromatin remodeling) (Taccioli et al, 1994), indicating that their association with AURORA A was based on protein–protein interactions.

We sought to determine whether AURORA A formed dimeric complexes with individual RNA-binding proteins or whether it was incorporated into larger multiprotein assemblies comprising multiple RNA-binding proteins simultaneously. To separate AURORA A-containing complexes based on their size and complexity, MV4-11 cells were lysed under native conditions, and the lysates were subjected to ultracentrifugation using a linear 5–50% sucrose gradient. Immunoblot analysis of AURORA A across every second of the 24 gradient fractions revealed two distinct populations: a “light” population peaking in fraction 4 and a “heavy” population peaking in fraction 18 corresponding to ~80S (Fig. 1I and EV1G). Biological replicate experiments (*n* = 3) showed that 59% of AURORA A migrated in the heavy fractions of the gradient (Fig. 1J). Overall, these findings indicated that AURORA A assembles into extensive protein–protein complexes during S-phase, and many interactors have not been previously identified as substrates of AURORA A in phosphoproteomic analyses (Fig. EV1H).

### Integrity of AURORA A protein complexes depends on RNA

**Figure 2:**
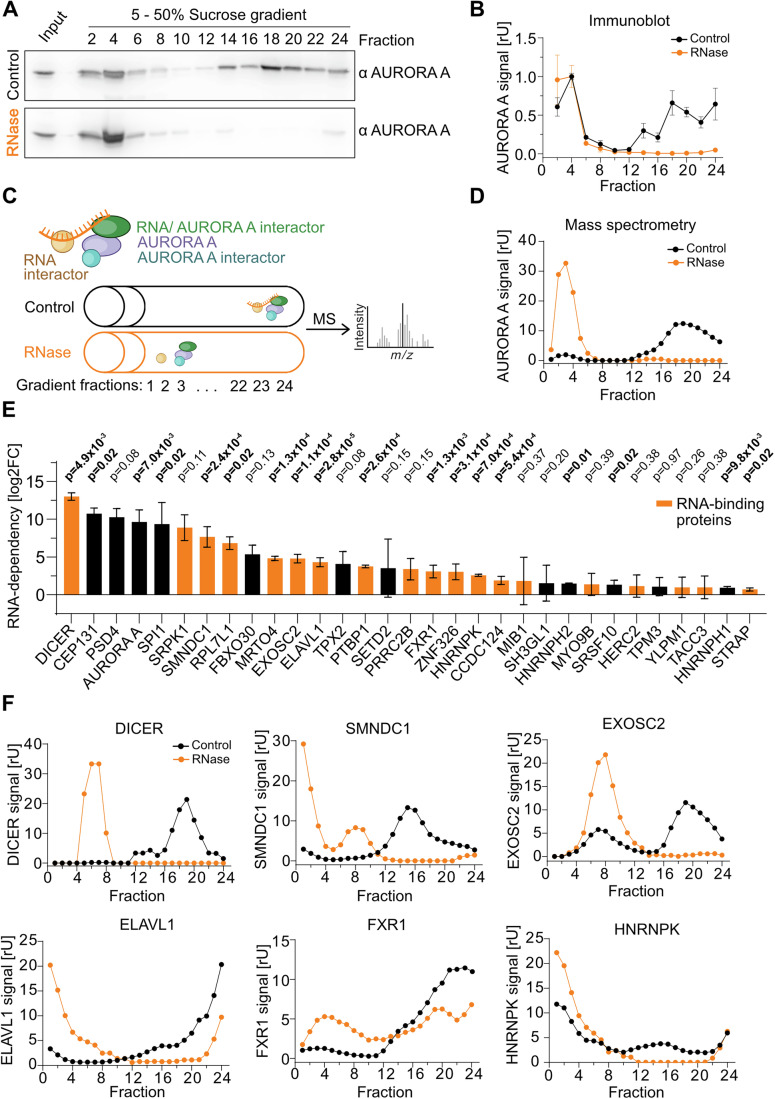
Integrity of AURORA A protein complexes depends on RNA. (**A**) Immunoblots of gradient fractions. MV4-11 HA-CRBN cells were lysed and treated with or without an RNase mixture. The lysate was loaded on a 5–50% sucrose gradient, centrifuged and fractionated. The precipitated fractions were loaded on an immunoblot. (**B**) Quantification of gradient centrifugation immunoblots ± RNase treatment. Quantification was performed with three biological replicates per condition. The AURORA A signal was normalized to the input, and the highest signal was set to 1.0. Each point represents the mean ± SEM. (**C**) Model of gradient centrifugation ± RNase to identify AURORA A high-molecular-weight complex partner via mass spectroscopy. (**D**) Sedimentation profiles of AURORA A in a 5–50% sucrose gradient analyzed by mass spectroscopy. MV4-11 HA-CRBN cell lysates were treated ±RNase, loaded on a 5–50% sucrose gradient, and individual fractions were analyzed by label-free mass spectroscopy. AURORA A intensities of three biological replicates were normalized to 100 and are shown for every fraction. (**E**) RNA-dependency of S-phase AURORA A interactors in gradient centrifugation. Gradient centrifugation and analysis were performed as described in (**D**). The RNA-dependency was calculated by the division of heavy (F12–F24) and light fractions (F1–F11) for DMSO and RNase conditions (DMSO and RNase score, respectively). The quotient of DMSO and RNase score was defined as RNA-dependency. Data were plotted for AURORA A interactors, which shift to lower fractions with RNase treatment. *P* values were calculated from three biological replicates using a two-sample unpaired *t*-test. AURORA A interactors were identified in an IP experiment (Fig. 1E), and RNA-binding proteins are labeled in orange. (**F**) Sedimentation profiles of selected AURORA A interactors from (E). Source data are available online for this figure.

**Figure EV2:**
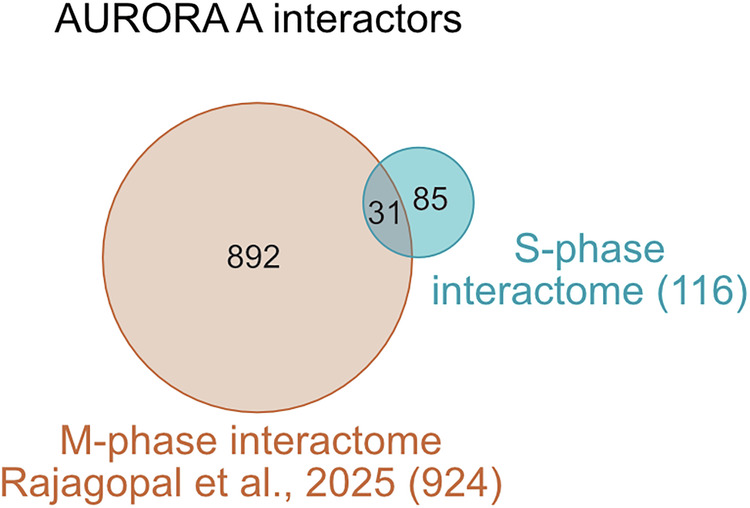
Integrity of AURORA A protein complexes depends on RNA. Overlay of the AURORA A S-phase interactome of this study with the published mitotic interactome (Rajagopal et al, 2025). Only interactors with the following cut-off are shown: enrichment HA-AURORA A versus control log2FC >3.

Given the high abundance of RNA-binding proteins in the AURORA A interactome, we next investigated whether the sedimentation behavior of the heavy AURORA A complexes was dependent on the presence of intact RNA. To this end, we repeated the sucrose gradient ultracentrifugation and fractionation, using lysates treated with an RNase mix. Strikingly, upon RNase treatment, AURORA A signals were nearly absent in the region around fraction 18, indicating a substantial shift of AURORA A complexes from high-molecular-weight fractions towards lower-molecular-weight fractions in the gradient (96% in light, 4% in heavy; Fig. 2A,B). The altered migration pattern of AURORA A after RNase treatment confirmed similar observations reported in a recent elegant study systematically exploring RNA-dependent proteins in mitotic cells (Rajagopal et al, 2025). However, the S-phase interactome of AURORA A differed fundamentally from its mitotic interactome (Fig. EV2), leaving it unclear which specific protein and RNA species are responsible for the observed massive shift of S-phase AURORA A complexes. We therefore aimed to identify the proteins that constituted the RNA-dependent high-molecular-weight AURORA A complex and to assess how RNase treatment affected the migration behavior of these proteins within the gradient.

To this end, we repeated the gradient centrifugation using RNase-treated and untreated lysates, but then systematically analyzed the protein content in each fraction by label-free mass spectrometry, based on the previously established R-DeeP protocol (Caudron-Herger et al, 2019). Mass spectrometric analysis was performed on 24 gradient fractions, with and without RNase treatment, in biological triplicates (Fig. 2C), resulting in the identification of 6679 proteins in total (Dataset EV3). We first examined the distribution of AURORA A across the gradient fractions and observed a pattern consistent with our immunoblot analyses: in untreated lysates, the majority of AURORA A detected across all fractions accumulated around fraction 18, whereas in RNase-treated lysates, AURORA A was no longer detectable in these heavy fractions but instead it peaked in the light fractions (fractions 2–4; Fig. 2D). To identify proteins with similar behavior, we next calculated the abundance ratio between the light (fractions 1–11) and heavy (fractions 12–24) halves of the gradient for all proteins interacting with AURORA A. Of all 116 proteins identified in the S-phase interactome, 18 showed a significant shift toward lighter fractions upon RNase treatment (Fig. 2E). Among these were 12 RNA-binding proteins, including DICER, SMNDC1, EXOSC2, ELAVL1, FXR1, and HNRNPK (Fig. 2E,F). We conclude that, in addition to AURORA A itself, numerous other AURORA A interactors form high-molecular-weight complexes in an RNA-dependent manner. The shift of many AURORA A-interacting proteins suggests that RNA may be required for complex integrity or that RNA itself contributes to the retention of the AURORA A protein complex in heavier fractions.

### AURORA A interacts with the C-linker domain of DICER

**Figure 3:**
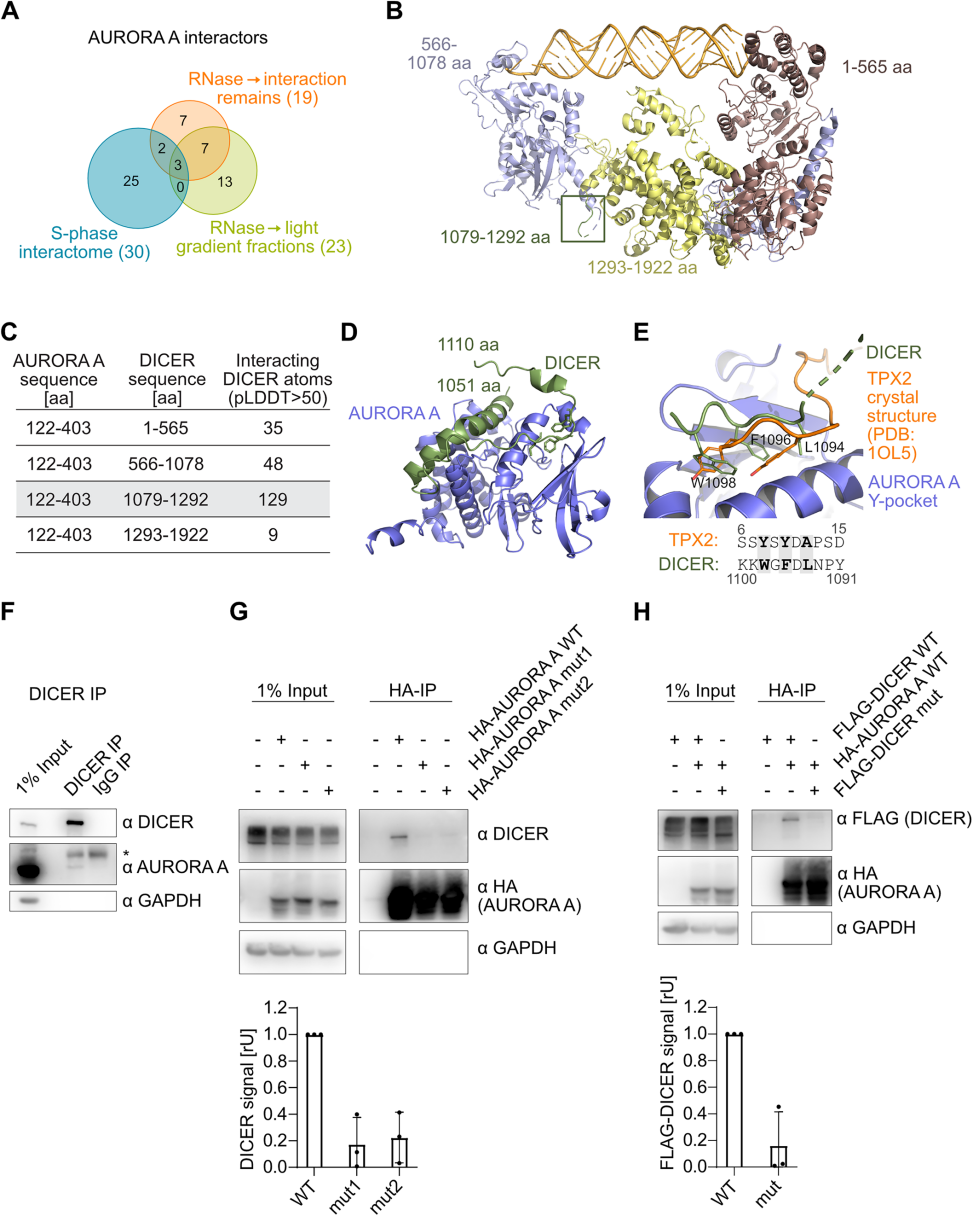
AURORA A interacts with the C-linker domain of DICER. (**A**) Venn diagram to select a representative protein from the RNA-dependent high-molecular-weight complex. The data of three experiments were overlaid: the S-phase interactome of AURORA A (Fig. 1E), RNA-binding proteins from the interactome study ±RNase (Fig. 1F, G), and GC AURORA A interactors, which shift with RNase treatment to lower fractions (Fig. 2E). The three overlapping proteins are YPLM1, FXR1, and DICER. (**B**) Cryo-EM structure of DICER in complex with RNA (PDB: 5ZAL) (Liu et al, 2018). The folded N-terminal helicase domain (brown), the pincer-like domain until connector domain (light blue) and the C-terminal RIIIDa domain (yellow) are shown. Marked in green is the predicted AURORA A interaction region (green, 1079–1292 aa) between the platform domain and the processing center. (**C**) List summarizing AlphaFold predictions for the catalytic AURORA A domain together with truncated DICER protein versions. For possible interacting atoms of DICER and AURORA A, exclusively atoms with a pLDDT score above 50 were taken into account. Possible interaction was defined as atoms of AURORA A and DICER at a distance of ≥5 Å. WT AURORA A: 1–403 aa; WT DICER: 1–1922 aa. aa amino acids, pLDDT per-residue measure of local confidence. (**D**) AlphaFold prediction of the catalytic domain of AURORA A (122–403 aa) and partial DICER (1051–1110 aa). The three DICER amino acids (L1094, F1096, and W1098), which are predicted to bind in the Y-pocket of AURORA A, are shown as sticks. (**E**) DICER (1051–1110 aa) and AURORA A (122–403 aa) predictions are overlayed with TPX2 crystal structure (PDB: 1OL5) (Bayliss et al, 2003). Top: DICER amino acids binding in the AURORA A Y-pocket are labeled. Bottom: Sequence alignment of TPX2 and DICER sequence, which binds in the AURORA A Y-pocket. Bold amino acids are important for binding the Y-pocket of AURORA A. (**F**) Immunoblot of endogenous DICER IP. Co-IP was performed with HEK293 cell lysate and magnetic beads coupled to a DICER antibody. The asterisk indicates the IgG heavy chain. (**G**) Top: Immunoblot of exogenous HA-AURORA A IP showing binding of AURORA A mutants to DICER WT. HEK293 cells were transfected with HA-AURORA A WT or HA-AURORA A mutants, and co-IP was performed using magnetic beads. Bottom: Quantification of DICER binding to AURORA A mutants. DICER signals in immunoblots were quantified in three biological replicate experiments. Data were normalized to WT interaction, and each bar represents the mean ± SD. HA-AURORA A mut1: W128E, I209R, Y197R, F157E, L169R, L178R, V182E, I184R, H187E, V252E, L188R, HA-AURORA A mut2: L169R, L178R, and V182E. (**H**) Top: Immunoblot of exogenous HA-AURORA A IP showing binding of DICER mutants to AURORA A WT. HEK293 cells were transfected with HA-AURORA A WT and co-transfected with FLAG-DICER WT or a FLAG-DICER mutant (Δ L1058–R1288), and co-IP was performed using magnetic beads. Bottom: Quantification of FLAG-DICER binding to AURORA A WT. FLAG-DICER signals in immunoblots were quantified in three biological replicate experiments. Data were normalized to WT interaction, and each bar represents the mean ± SD. Source data are available online for this figure.

The shift in AURORA A distribution upon RNase treatment during gradient centrifugation was striking, yet unexpected, given that AURORA A lacks a canonical RNA recognition motif (RRM). We therefore hypothesize that AURORA A is associated with RNA indirectly, through interactions with RNA-binding proteins that recruit it into RNA-containing protein complexes. To identify candidate RNA-binding proteins, we applied three selection criteria: First, they were identified as RNA-binding proteins within the AURORA A S-phase interactome by co-immunoprecipitation. (*n* = 30; Fig. 1E), second, they were proteins that shifted into lighter gradient fractions after RNase treatment, similar to AURORA A (*n* = 23; Fig. 2E) and third, their interaction with AURORA A remained stable following RNase treatment (*n* = 19; Fig. 1G), indicating a direct protein–protein interaction with AURORA A (Fig. 3A). In total, our proteomic experiments identified the RNA-binding proteins DICER, FXR1, and YLPM1 as candidate proteins.

**Figure EV3:**
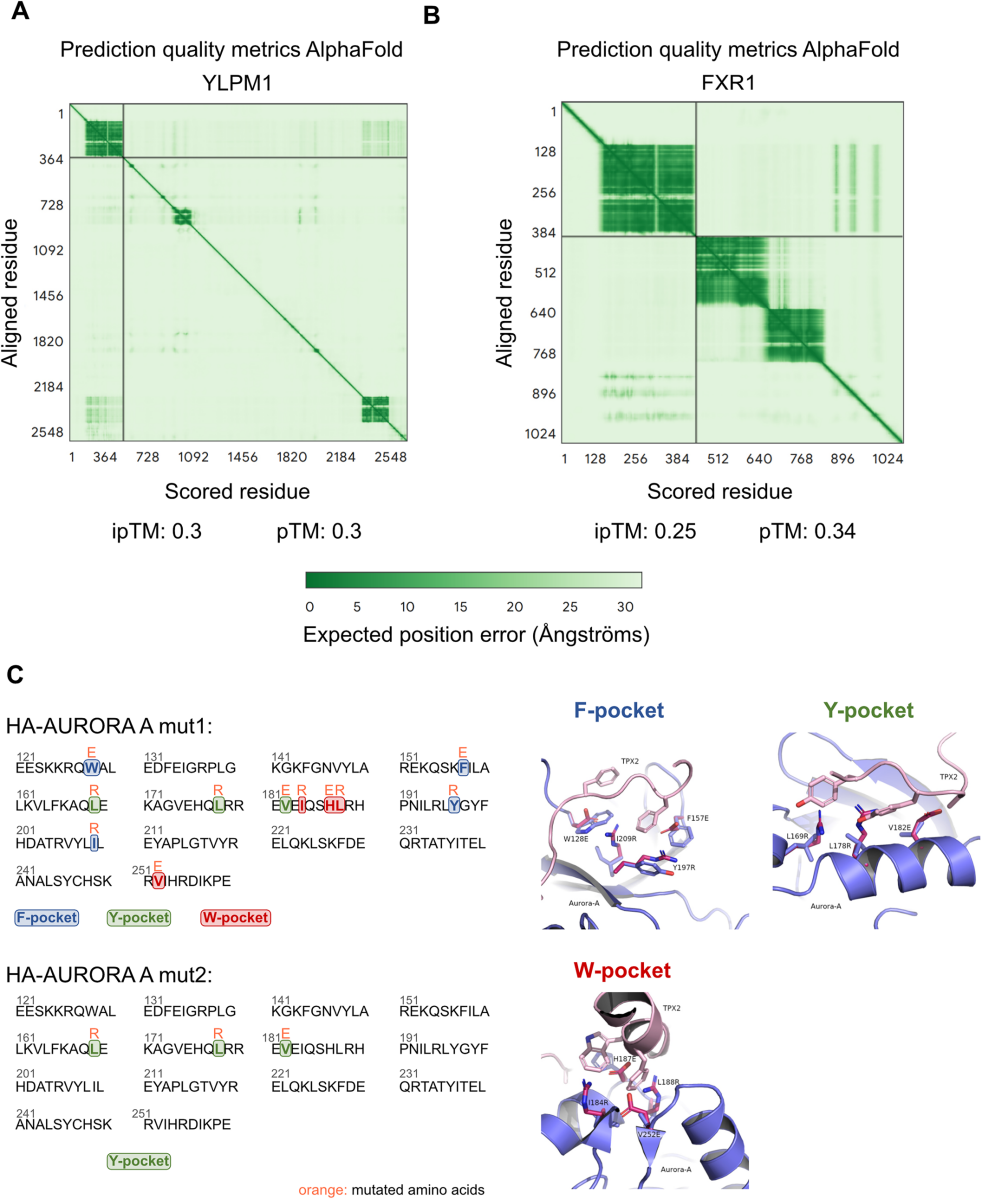
AURORA A interacts with the C-linker domain of DICER. (**A**) Predicted alignment error plot for the interaction of full-length AURORA A and full-length YLPM1. For predictions, AlphaFold 3 was used, and the best hit is shown here. ipTM interface predicted template modeling score, pTM predicted template modeling score. (**B**) As in (**A**), but here the prediction of the interaction of full-length AURORA A and full-length FXR1 is depicted. (**C**) Mutated amino acids in AURORA A F-, Y-, and W-pocket. Left: The WT AURORA A sequence is shown here (from amino acid 121 to 260). All amino acids which were mutated in this study are colored according to the TPX2 binding pockets. Exchanged amino acids are colored in orange. Right: Crystal structure of AURORA A with TPX2 (PDB: 1ol5) (Bayliss et al, 2003). TPX2 binding pockets of AURORA A are shown. Mutated amino acids of AURORA A are annotated.

To identify potential direct interactors of AURORA A, we next performed AlphaFold-based structural modeling of all three candidate proteins together with AURORA A. While these analyses identified no substantial interfaces between AURORA A and YLPM1 (ipTM = 0.3; Fig. EV3A) or FXR1 (ipTM = 0.25; Fig. EV3B), they revealed putative protein–protein interfaces between AURORA A and DICER. Since DICER is a huge protein (1922 aa, amino acids), DICER was divided in four parts based on the domain organization: the helicase domain (1–565 aa), the pincer-like domain (566–1078 aa), the C-linker (1079–1292 aa) and the RIIIDa domain (1293–1922 aa) (Lee et al, 2023) (Fig. 3B). Neither the helicase domain, nor the pincer-like domain nor the C-terminal RIIIDa domain showed significant interactions. However, the so-called C-linker domain, an intrinsically unstructured region showed reliable AURORA A contacts (129 amino acids with pLDDT > 50; Fig. 3C). A screen with additional DICER fragments defined a minimal interaction domain comprising an α-helix of the pincer-like domain together with the N-terminal portion of the C-linker domain (aa 1051–1110, Fig. 3D). On the AURORA A side, the model predicted that DICER bound to the so-called Y-pocket (Fig. 3E). The Y-pocket is a region of AURORA A, previously shown to interact with its well-characterized binding partner TPX2 via two tyrosine residues (“Y”) as hotspot interactions sites, in addition to TPX2-interactions to the F- and W-pocket of AURORA A (Bayliss et al, 2003; McIntyre et al, 2017). Interestingly, the predicted binding mode of DICER to AURORA A closely resembles that of TPX2, with aromatic amino acids interacting with the Y-positions (1102, 1104) and an additional leucine (1094) at a position where TPX2 exhibits a hydrophobic residue (A11) as well (Fig. 3E).

To investigate the interaction between AURORA A and DICER, we first performed immunoprecipitation experiments using endogenous proteins in HEK293 cells. Immunoprecipitation of DICER co-precipitated AURORA A, whereas control immunoprecipitations using nonspecific IgG antibodies did not detect AURORA A (Fig. 3F). To validate the binding sites suggested by the AlphaFold model, we then performed immunoprecipitation experiments using HEK293 cells stably expressing HA-tagged AURORA A and endogenous DICER. HA-affinity beads efficiently co-immunoprecipitated DICER from cells expressing HA-AURORA A, but not from parental control cells lacking exogenous protein expression (Fig. 3G). In contrast, mutation of AURORA A at the Y, F, and W pocket (“HA-AURORA A mut1”, nine missense mutations; Fig. EV3C) or mutation of the Y-pocket alone (“HA-AURORA A mut2”, three missense mutations; Fig. EV3C) reduced DICER binding to near-background levels (Fig. 3G).

To validate the AURORA A interaction domain of DICER, we finally expressed exogenous FLAG-tagged DICER together with HA-tagged AURORA A in HEK293 cells (Fig. 3H). This experiment confirmed a robust interaction of wildtype DICER with AURORA, when precipitated with HA-affinity beads. In contrast, expression of a DICER mutant lacking the interaction domain determined by alpha fold modeling (aa 1057–1287) markedly reduced co-immunoprecipitation efficiency, indicating that this region was required for the interaction with AURORA A (Fig. 3H). These results supported the conclusion that AURORA A and DICER interacted through structurally defined protein-binding domains.

### AURORA A and DICER are in close cellular proximity

**Figure 4:**
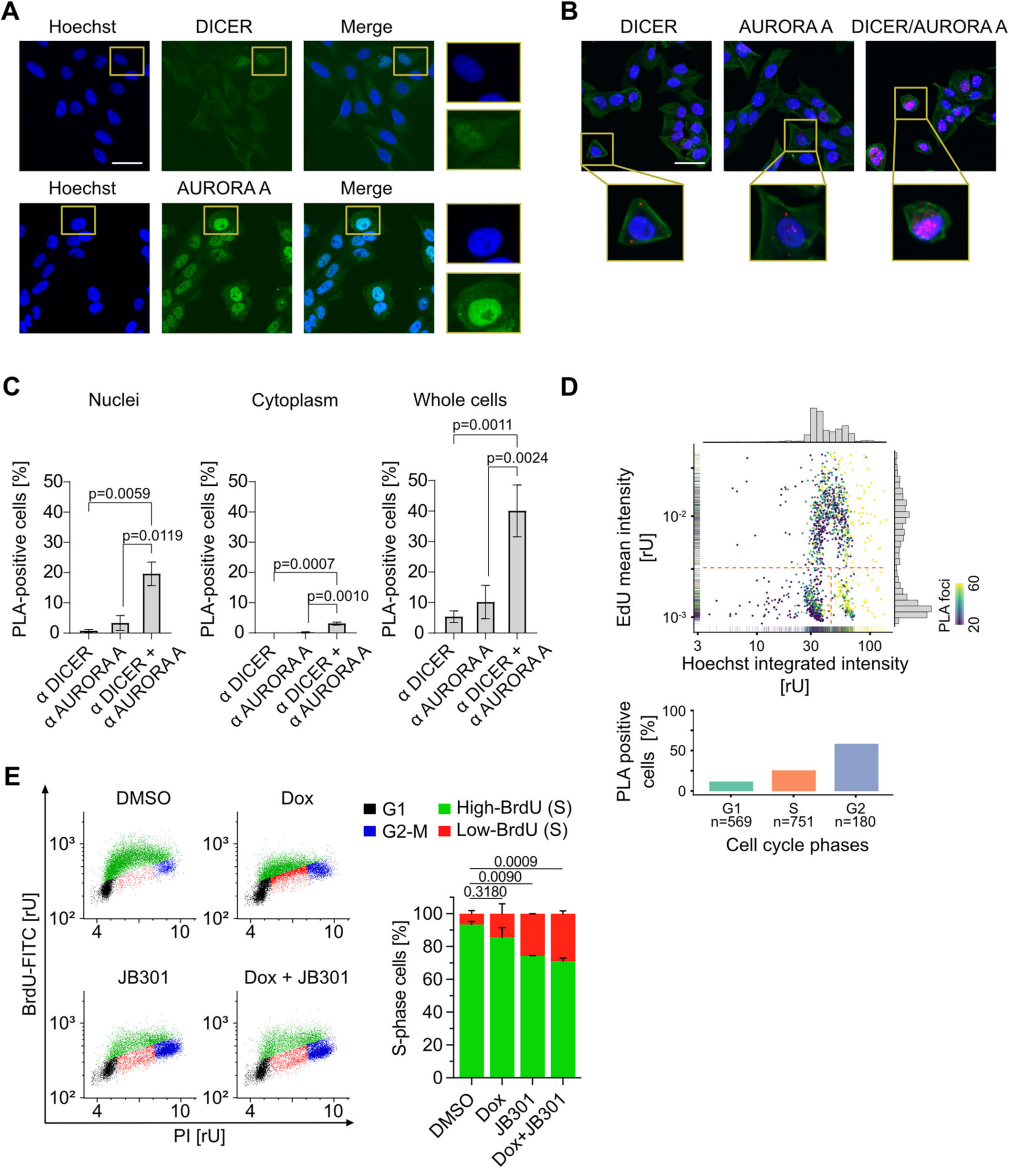
AURORA A and DICER are in close cellular proximity. (**A**) Immunofluorescence images of AURORA A and DICER showing its subcellular distribution in U2OS cells. Images were acquired with a 10x objective. Scale bar 50 µm. Example cells were picked and are shown on the right. For DICER, an example cell with a high signal in the nucleus was chosen. (**B**) Immunofluorescence images of PLAs between DICER and AURORA A in U2OS cells. Images were taken using a 10x objective and are labeled with the antibodies used for the PLA (DICER and AURORA A correspond to single-antibody controls, DICER/AURORA A: PLA). Red dots (λ647): intensity centers of proximity pairs, blue (λ405): Hoechst staining for nucleus, green (λ568): phalloidin staining for cytoskeleton. (**C**) Quantification of PLA-positive cells. The PLA spot intensity was analyzed and summarized for every nucleus, cytoplasm, or the whole cell in every field of view (FoV). Nuclei, cytoplasm or whole cells with a higher intensity of 25 (rU) were defined as positive. For each condition, three biological replicate experiments were conducted, and three FoVs were analyzed (total analyzed cells, DICER: 500, AURORA A: 429, DICER + AURORA A: 498). Data were normalized to the total number of cells in every FoV. Each bar represents the mean ± SEM. The *p* value between the boxes represents the Tukey’s multiple comparison test. (**D**) Cell cycle-resolved analysis of DICER/AURORA A PLA in U2OS cells. Scatter plot (top panel) shows individual nuclei (*n* = 1500) as dots plotted according to DNA content (Hoechst integrated intensity, log scale) and DNA synthesis (EdU mean intensity, log scale) on the x- and y-axis, respectively, enabling cell cycle assignment. Dots were colored according to the number of PLA foci per nucleus. The color scale was limited from 20 to 60 PLA foci, with values outside this range squished to the nearest limit. Marginal histograms represent the distribution of Hoechst and EdU intensities. Bar plot (bottom panel) shows the percentage of PLA-positive nuclei (defined as nuclei with >50 PLA dots) quantified per cell cycle phase. (**E**) Cell cycle distribution analyzed by flow cytometry. Unsynchronized MV4-11 cells expressing shDICER-4 (doxycycline inducible) were treated with doxycycline (1 µg/ml) or EtOH as a control for 2 days, followed by DMSO or 200 nM JB301 for 16 h. Doxycycline-treated cells are labeled with shDICER in the figure. 16 h prior BrdU staining, indicated cells were treated with 200 nM of JB301 or DMSO as a control. Cells were labeled with BrdU and stained with PI, followed by analysis with flow cytometry. The percentage of cells in S-phase (High-BrdU: green and Low-BrdU: red) was determined in biological triplicate experiments (**p < 0.01, ***p < 0.001 unpaired two-tailed Welch’s *t*-test), shown as average ±  SEM. All cell cycle phases are quantified in Fig. EV4F. Source data are available online for this figure.

**Figure EV4:**
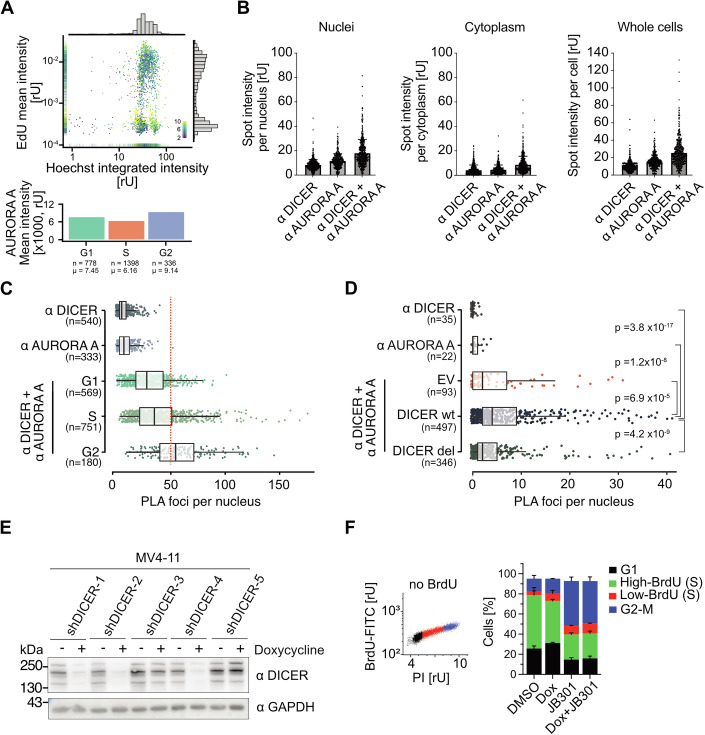
AURORA A and DICER are in close cellular proximity. (**A**) Cell cycle-resolved analysis of AURORA A immunofluorescence in U2OS cells. Scatter plot (top panel) shows individual nuclei (*n* = 2512) as dots plotted according to DNA content (Hoechst integrated intensity, log scale) and DNA synthesis (EdU mean intensity, log scale) on the x- and y-axis, respectively, enabling cell cycle assignment. Dots were colored according to the AURORA A immunofluorescence intensity per nucleus (x 1000 a.U.). The color scale of AURORA A mean intensity (x 1000 a.U.) was limited from 2 to 10, with values outside this range squished to the nearest limit. Marginal histograms represent the distribution of Hoechst and EdU intensities. Bar plot (bottom panel) shows the mean AURORA A immunofluorescence (x 1000 a.U.) signal quantified per cell cycle phase (*n* number of nuclei, μ mean, AU arbitrary units). (**B**) Quantification of spot intensity. The PLA spot intensity was analyzed and summarized per nucleus (left), cytoplasm (middle), or whole cell (right). For each condition (DICER, AURORA A, or DICER + AURORA A) three biological replicate experiments were conducted and in each case three FoVs were analyzed (total analyzed cells, DICER: 500, AURORA A: 429, DICER + AURORA A: 498). Each bar represents the mean ± SD. (**C**) Quantification of DICER/AURORA A PLA in cell cycle phases of U2OS cells. Jittered dot plot showing the number of PLA foci per nucleus for the cell cycle phases identified in U2OS cells from Fig. 4D. Overlaid boxplots show the median (center line), interquartile range (box, Q1–Q3), and whiskers extending to the most extreme values within 1.5 x interquartile range. The number of nuclei analyzed per condition is indicated as *n* (*n* ≥ 333). The dotted orange line marks the threshold of 50 PLA foci per nucleus used to classify PLA-positive cells in Fig. 4D. (**D**) Quantification of DICER/AURORA A PLA in DICER knock-out cells. Jittered dot plot showing the number of PLA foci per nucleus for the combined PLA condition in DICER knock-out cells treated transiently overexpressing of wildtype (DICER wt) and mutant DICER (DICER del) and for the single-antibody controls (DICER only and AURORA A only). Overlaid boxplots show the median (center line), interquartile range (box, Q1–Q3), and whiskers extending to the most extreme values within 1.5 x interquartile range. The number of nuclei analyzed per condition (*n*) is indicated below the corresponding condition. *p* values were calculated with the Wilcoxon test. (**E**) Western blot of 5 different doxycycline-inducible shDICER MV4-11 cell lines. Cells were treated with doxycycline (1 µg/ml) or EtOH as a control for 3 days. GAPDH was used as a loading control. (**F**) Left: BrdU-PI cell cycle profile of unsynchronized MV4-11 cells stably expressing doxycycline-inducible shDICER-4 without BrdU incorporation, serving as a control for Low-BrdU S-phase cells in Fig. 4E. Right: Quantification of BrdU-PI cell cycle distribution from three independent biological replicates shown as average ± SEM. One representative replicate is displayed in Fig. 4E.

Since immunoprecipitation and proteomic analyses were performed on cellular lysates, we next examined the subcellular distribution of AURORA A and DICER in intact cells using immunofluorescence and proximity ligation assays (PLAs). We first immunostained endogenous AURORA A and DICER in U2OS osteosarcoma cells using specific primary antibodies, followed by fluorescently labeled secondary antibodies. As expected, based on the cytoplasmic function of DICER in micro-RNA processing, the majority of DICER was located in the cytosol (Fig. 4A). However, our analysis suggested the presence of a minor yet significant nuclear fraction of DICER, consistent with previous reports indicating a nuclear function of DICER, such as reducing DNA/RNA-hybrid structures (R-loops) (Alagia et al, 2025; Burger and Gullerova, 2018; Burger et al, 2017; Camino et al, 2023; Cheng et al, 2025; White et al, 2014). Immunostaining of AURORA A showed that the protein was predominantly localized in the nucleoplasm, with a minor cytosolic fraction (Fig. 4A). Since the expression of AURORA A is regulated throughout the cell cycle, we next labeled cells with EdU and then stained the total cellular DNA (Hoechst), newly synthesized DNA (EdU) and AURORA A. Scatter plot analysis revealed highest AURORA A expression in G2/M-phase cells, while cells in G1 and S-phase express less but significant amounts of AURORA A (Fig. EV4A).

The partial nuclear localization of both endogenous AURORA A and DICER observed by immunofluorescence prompted us to perform PLAs to further assess their association within cells, using the same primary antibodies. Strikingly, U2OS cells treated with both antibodies exhibited a strong, predominantly nuclear, PLA signal compared to cells incubated with only one of the respective antibodies (Figs. 4B,C and EV4B). We then repeated the PLA experiment, this time again performing EdU labeling and Hoechst staining and estimated the amount of PLA-positive cells in different cell cycle phases. While G2/M-phase cells showed the highest fraction of PLA-positive cells, levels in S-phase cells were higher than in G1 or background controls (Figs. 4D and EV4C). This is particularly compelling, given that AURORA A expression is lowest during S-phase.

We next performed PLA experiments with exogenously expressed DICER lacking the AURORA A-interacting domain (aa 1057–1287) in DICER knock-out cells. In contrast to wildtype DICER, which again showed abundant PLA-positive cells, the mutant displayed markedly reduced PLA signals with AURORA A (Fig. EV4D). We concluded that AURORA A and DICER are in close proximity in situ in cultured human cancer cells and sought to determine whether the two proteins also exhibit a functional or genetic interaction. We therefore generated MV4–11 cell lines stably transduced with five different, doxycycline-inducible shRNAs targeting DICER and analyzed the reduction of DICER expression by immunoblotting after 3 days of doxycycline treatment (Fig. EV4E). We then selected the cell line exhibiting the strongest DICER depletion (78% reduction), and combined doxycycline treatment with PROTAC-mediated (JB301) degradation of AURORA A. Subsequent BrdU-PI FACS experiments demonstrated that the combined depletion of both proteins induced a rampant S-phase arrest (Figs. 4E and EV4F). We concluded that AURORA A and DICER could cooperate to ensure proper S-phase progression during the cell cycle in MV4-11 cells.

### AURORA A supports the association of SETD2 with high-molecular-weight complexes and chromatin

Our analyses so far demonstrated that S-phase AURORA A associates with RNA-binding proteins and that RNA is required for maintaining the integrity of this complex. However, these findings did not clearly reveal a critical effector function of AURORA A within the complex. Notably, PROTAC-mediated degradation–but not enzymatic inhibition–of AURORA A induces S-phase defects (Fig. 1D) (Adhikari et al, 2020). Based on this observation, we hypothesize that S-phase AURORA A acts as a scaffold protein required for the association of other complex members. To identify such proteins, we repeated gradient centrifugation followed by mass spectrometric analysis, this time in the presence and absence of AURORA A.

**Figure 5:**
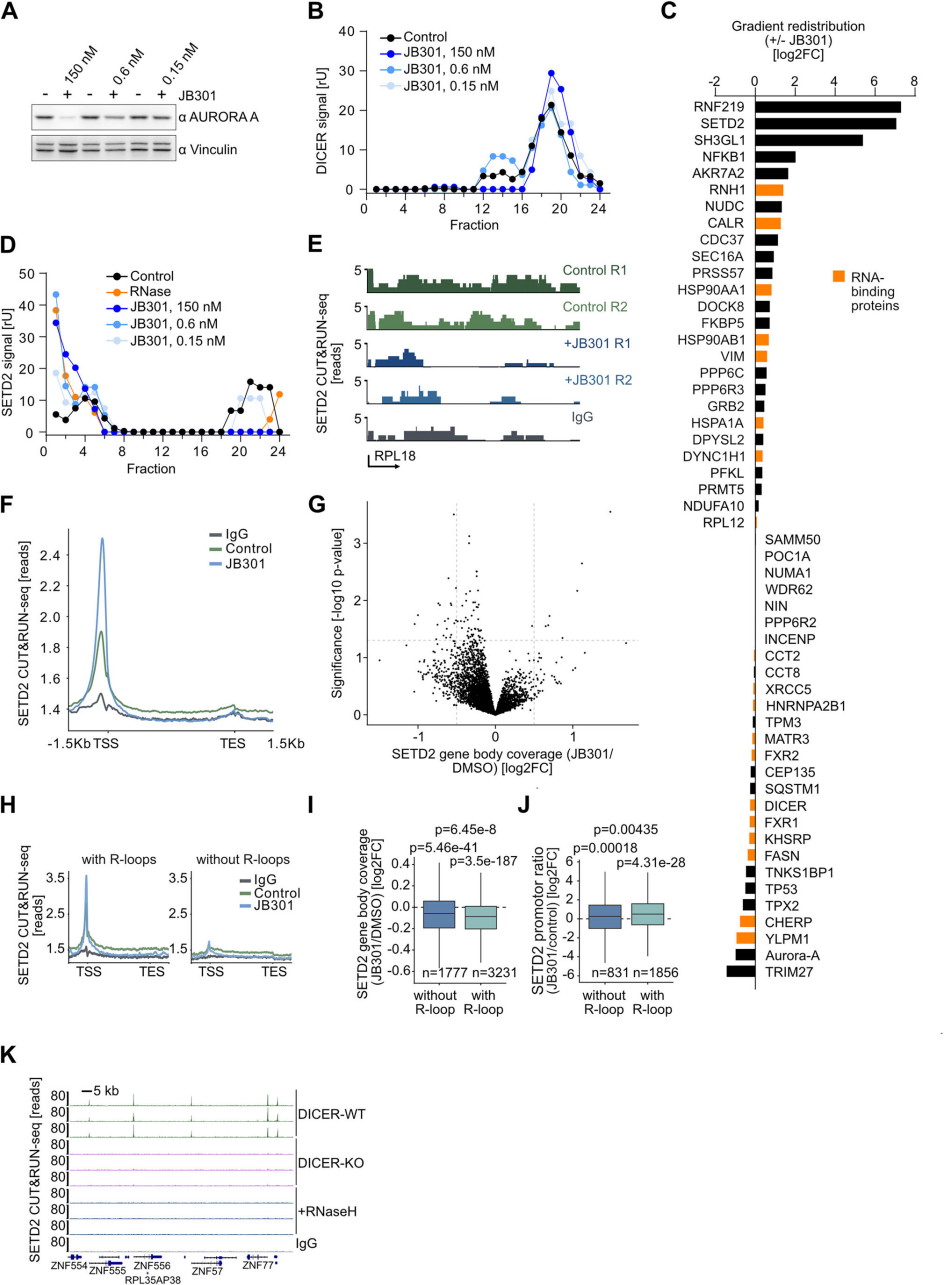
AURORA A supports the association of SETD2 with high-molecular-weight complexes and chromatin. (**A**) Immunoblot with input lysates for gradient centrifugation. MV4-11 cells overexpressing HA-CRBN were treated with different concentrations of JB301 for 6 h. (**B**) Sedimentation profiles of DICER in a 5–50% sucrose gradient analyzed by mass spectroscopy. MV4-11 were treated ±JB301 for 6 h, lysed and loaded on a 5–50% sucrose gradient. Individual fractions were analyzed by label-free mass spectroscopy. DICER intensities were normalized to 100 and are shown for every fraction. (**C**) PROTAC-dependency of AURORA A interactors in gradient centrifugation. Gradient centrifugation and analysis were performed as described in (**B**). The PROTAC-dependency was calculated by the division of heavy (F12–F24) and light fractions (F1–F11) for DMSO and PROTAC conditions (0.6 nM and 150 nM, DMSO and PROTAC score, respectively). The quotient of DMSO and PROTAC score was defined as PROTAC-dependency. Data were plotted for all AURORA A interactors. AURORA A interactors were identified in an IP experiment (Fig. 1G,H), and RNA-binding proteins are labeled in orange. (**D**) Sedimentation profiles for control, RNase and PROTAC conditions of SETD2 in a 5–50% sucrose gradient analyzed by mass spectroscopy. Gradient centrifugation and analysis were performed as described in (**B**), and SETD2 LFQ intensities were normalized to 100 and are shown for every fraction. (**E**) Genome browser tracks of the SETD2 CUT&RUN-seq signal in MV4-11 cells treated with DMSO or 150 nM JB301 for 6 h. The binding of the *RPL18* gene is shown and compared to IgG as a control. (**F**) Metagene plot of SETD2 CUT&RUN-seq signals in expressed genes (*n* = 10,821). Mean binding of SETD2 in MV4-11 cells treated with DMSO (*n* = 2, biological replicates) or 150 nM JB301 (*n* = 3, biological replicates) for 6 h compared to the IgG control. (**G**) Volcano plot of SETD2 CUT&RUN-seq signal as in panel (**F**) in the gene bodies of expressed genes(*n* = 5008). *p* value, Wald test. (**H**) Metagene plots of SETD2 CUT&RUN-seq signals, as in panel (**F**) in expressed genes with (*n* = 7118) and without (*n* = 3703) R-loops, respectively. (**I**) Box plot of the change (log2FC) in the SETD2 gene body reads as in panel (**G**) in expressed genes with (*n* = 3231) and without (*n* = 1777) R-loops. Boxplots show the median (center line), interquartile range (box, Q1–Q3), and whiskers extending to the most extreme values within 1.5 x interquartile range. Values outside this range are not shown. *p* values above the respective box represent the one-sample Wilcoxon signed-rank test against 0 (no change). The *p* value between the two boxes represents the Wilcoxon signed-rank test. (**J**) Box plot of the change (log2FC) in the SETD2 promoter ratio (i.e., the ratio of IgG control-subtracted, length-normalized reads in the promoter and gene body) in expressed genes with (*n* = 1856) and without (*n* = 831) R-loops. Boxplots show the median (center line), interquartile range (box, Q1–Q3), and whiskers extending to the most extreme values within 1.5 x interquartile range. Values outside this range are not shown. *p* values above the respective box represent the one-sample Wilcoxon signed-rank test against 0 (no change). The p-value between the two boxes represents the Wilcoxon signed-rank test. (**K**) Genome browser tracks of the SETD2 CUT&RUN-seq signal of DICER wildtype, DICER knock-out MV4-11 cells and DICER wildtype MV4-11 cells treated with RNase H after cell permeabilization. Protein-coding genes are labeled with their HGNC symbol. IgG, negative control. Source data are available online for this figure.

**Figure EV5:**
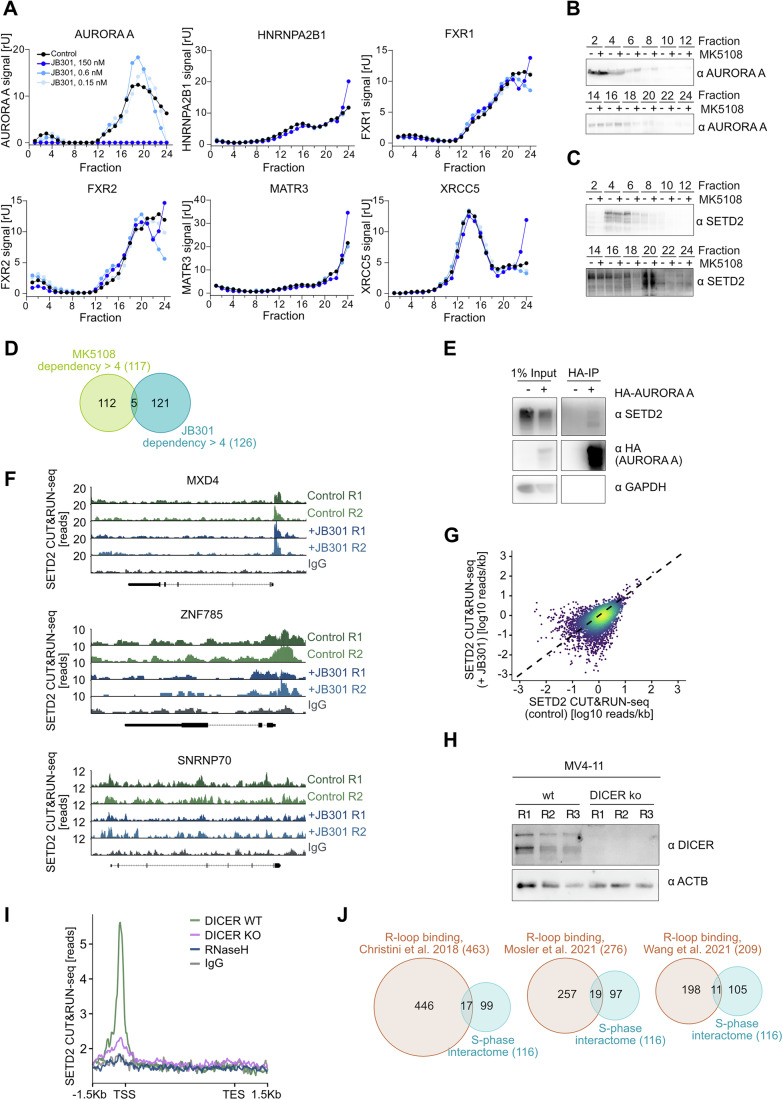
AURORA A supports the association of SETD2 with high-molecular-weight complexes and chromatin. (**A**) Sedimentation profiles of RNA-dependent AURORA A interactors in a 5–50% sucrose gradient analyzed by mass spectroscopy. MV4-11 overexpressing HA-CRBN were treated ±JB301 for 6 h, lysed and loaded on a 5–50% sucrose gradient. Individual fractions were analyzed by label-free mass spectroscopy. LFQ intensities were normalized to 100 and are shown for every fraction. (**B**) Immunoblots of gradient fractions. MV4-11 cells were treated with or without an MK5108 (150 nM) for 6 h. The lysate was loaded on a 5–50% sucrose gradient, centrifuged and fractionated. The precipitated fractions were loaded on an immunoblot, and AURORA A was detected by specific antibodies. (**C**) Immunoblots of gradient fractions. MV4-11 cells were treated with or without an MK5108 (150 nM) for 6 h. The lysate was loaded on a 5–50% sucrose gradient, centrifuged and fractionated. The precipitated fractions were loaded on an immunoblot, and SETD2 was detected by specific antibodies. Please note that the lower membrane was exposed for a longer duration to better visualize the differential gradient migration behavior of SETD2 induced by MK5108. (**D**) Global analysis of AURORA A PROTAC and inhibitor dependency. MV4-11 HA-CRBN cells were treated with either JB301 (150 and 0.6 nM) or with MK5108 (150 nM) for 6 h. The lysate was loaded on a 5–50% sucrose gradient, centrifuged, fractionated, and the proteins were analyzed by label-free mass spectroscopy. The PROTAC/ inhibitor-dependency was calculated by the division of heavy (F12–F24) and light fractions (F1–F11) for DMSO and PROTAC or inhibitor conditions, respectively. The quotient of DMSO and PROTAC/inhibitor score was defined as PROTAC-/inhibitor-dependency, respectively. For the Venn diagram, only proteins which were detected in both conditions were taken into consideration. (**E**) Immunoblot of exogenous HA-AURORA A IP showing binding of AURORA A to SETD2 WT. HEK293 cells were transfected with HA-AURORA A WT, and co-IP was performed using magnetic beads. (**F**) Genome browser track of the SETD2 CUT&RUN signal in MV4-11 cells treated with DMSO or 150 nM JB301 for 6 h. The binding of the MXD4, SNRNP70, and ZNF785 genes are shown and compared to IgG as a control. (**G**) Scatter plot of IgG control-subtracted SETD2 CUT&RUN-seq signals in the gene body (TSS + 1 kb until TES) of MV4-11 cells treated with DMSO or 150 nM JB301 for 6 h. (**H**) Immunoblot of DICER levels in wt and Dicer KO MV4-11 cells. ß-Actin was used as a loading control (ACTB). (**I**) Metagene plots of mean SETD2 CUT&RUN-seq signals of DICER wildtype, DICER knock-out MV4-11 cells and DICER wildtype MV4-11 cells treated with RNase H after cell permeabilization in expressed genes (*n* = 10,821) compared to IgG as a negative control. (**J**) Identification of R-loop binding AURORA A interactors. Various R-loop databases were screened for AURORA A interactors. The databases are from three different publications (Cristini et al, 2018; Mosler et al, 2021; Wang et al, 2018), each of which employs a distinct approach to identifying R-loop-binding proteins.

To acutely reduce AURORA A protein levels, we treated MV4-11 cells with increasing concentrations of the AURORA A PROTAC JB301. Maximal degradation was observed at 150 nM (90% depletion), whereas treatment with 0.6 nM or 0.15 nM resulted in only partial degradation (30 and 20%, respectively; Fig. 5A). We next prepared cell lysates, separated protein complexes by gradient centrifugation, and analyzed the protein composition of all gradient fractions by mass spectrometry in PROTAC-treated and control cells. In contrast to RNase treatment, AURORA A degradation did not cause a shift of DICER (Fig. 5B) or of any of the other interacting RNA-binding proteins into lighter gradient fractions (Fig. EV5A). However, three other AURORA A interacting proteins shifted from heavy to light gradient fractions upon AURORA A degradation: RNF219, SETD2, and SH3GL1 (Fig. 5C). To analyze if the shift of these proteins upon PROTAC treatment is based on AURORA As enzymatic function, we repeated the gradient centrifugation experiment, but this time with the AURORA A kinase inhibitor MK5108. Immunoblot analysis revealed, that MK5108 did not induce a shift of AURORA A, nor of SETD2 into lighter gradient fractions (Fig. EV5B,C). Mass spectrometry analysis further revealed, that of all 126 proteins shifting upon PROTAC treatment (proteome-wide), only five showed the same shift upon catalytic inhibition (Fig. EV5D). This indicated that the AURORA A protein, but not its enzymatic function, was required to maintain these proteins within large protein complexes in MV4-11 cells.

The shift of SETD2 (Su(var) 3–9 Enhancer of zest and Trithorax domain-containing 2) was particularly compelling, as it occurred in a dose-dependent manner and was complete at high PROTAC concentrations (Fig. 5D). For this reason, and because SETD2 also shifted from heavy to light gradient fractions upon RNase treatment (Fig. 5D), we subsequently focused on the role of SETD2 within the AURORA A complex. SETD2 is a histone methyltransferase that associates with RNA polymerase II during transcription, inducing trimethylation of H3K36 and thereby inhibiting spurious transcription (Markert et al, 2025; Molenaar and van Leeuwen, 2022). SETD2 function critically depends on its association with chromatin. Given the strong effect of AURORA A degradation on SETD2 abundance within high-molecular-weight complexes, we first validated its interaction with AURORA A by co-immunoprecipitation (Fig. EV5E) and then sought to investigate whether AURORA A influences the chromatin association of SETD2. We therefore again depleted AURORA A using JB301 and subsequently performed CUT&RUN experiments with a SETD2-specific antibody to analyze SETD2 chromatin association genome-wide. As controls, we repeated the experiment in cells retaining AURORA A and using a nonspecific control antibody. In control cells, consistent with its association with the transcription machinery, CUT&RUN reads obtained with the SETD2 antibody were enriched in genic regions compared to the nonspecific IgG control (Figs. 5E,F, and EV5F). In agreement with published experiments (Guo et al, 2022; Sun et al, 2023) and similar to RNA polymerase II profiles, SETD2 CUT&RUN signals spanned the entire genic region from the transcription start site to the transcription end site, with prominent peaks at the promoter-proximal pause site and downstream of the transcription end site. Strikingly, PROTAC-mediated degradation of AURORA A caused a pronounced redistribution of SETD2 CUT&RUN signals. While read density increased at the promoter-proximal pause site, SETD2 occupancy within gene bodies was almost reduced to background levels, as observed in analyses of individual genes (Figs. 5E and EV5F) and in metagene analysis across all expressed genes (Fig. 5F). To determine whether this redistribution was driven by a subset of genes or represented a global effect, we next compared SETD2 binding across individual genes. Direct comparison of read densities revealed decreased SETD2 occupancy in the bodies of most expressed genes (Figs. 5G and EV5G).

We next investigated whether distinct gene subsets were particularly affected by SETD2 loss. Strikingly, this analysis revealed a strong association between R-loops and SETD2 chromatin binding. We classified genes as R-loop-containing if they had been reported in at least four independent R-loop mapping experiments, according to the R-loopBase repository (6273 R-loop-containing genes, 4552 R-loop-free genes) (Lin et al, 2022). R-loop-containing genes displayed higher SETD2 genic occupancy compared to R-loop-free genes, as demonstrated by metagene analysis (Fig. 5H). Strikingly, PROTAC-mediated degradation of AURORA A led to a more pronounced reduction in genic SETD2 occupancy on R-loop-containing genes (Fig. 5I) and to a stronger shift from gene body to transcription start site-associated SETD2 coverage (Fig. 5J) when compared to R-loop-free genes. The finding that the intact DICER/AURORA A/SETD2 complex most prominently mediated SETD2 binding to R-loop–containing or R-loop–sensitive genes was particularly intriguing, given that DICER has been reported to bind R-loops and suppress their levels (Alagia et al, 2025; Camino et al, 2023). We therefore repeated the SETD2 CUT&RUN experiment following RNase H–mediated R-loop degradation after cell permeabilization and in DICER knock-out cells (Fig. EV5H). Strikingly, under both conditions, SETD2 binding was reduced to near-background levels, as shown by browser tracks and metagene analysis (Figs. 5K and EV5I). Moreover, 32 of the 116 AURORA A-interacting proteins have been identified in at least one systematic study as R-loop–associated factors (Fig. EV5J; Dataset EV4). Taken together, our data indicate that PROTAC-mediated degradation of AURORA A, as well as depletion of DICER and R-loop degradation, led to a profound reduction of SETD2 occupancy at RNA polymerase II–transcribed genes, particularly in genes enriched for R-loops

## Discussion

Consistent with the well-established role of the protein kinase AURORA A in early mitosis, purely catalytic AURORA A inhibitors arrest cells at the G2/M-transition. In contrast, PROTAC-mediated depletion of AURORA A does not induce a pronounced G2/M arrest, possibly because mitotic AURORA A is protected from degradation (Wang et al, 2021). Instead, AURORA A PROTACs cause an S-phase arrest in tumor cells, pointing to a scaffolding function of AURORA A during S-phase. In N-MYC-expressing cells, this role has been linked to the AURORA A/N-MYC complex; however, similar phenotypes are also observed in N-MYC-negative cells, where the molecular basis of this scaffolding function remained entirely unclear. AURORA A PROTACs hold considerable therapeutic potential, as the S-phase function of AURORA A may represent a targetable vulnerability in cancer cells (Nelson et al, 2025; Pogash and Fletcher, 2025; Sflakidou et al, 2024; Tang et al, 2025; Wang et al, 2021). However, PROTACs also serve as versatile tools to dissect the scaffolding functions of cellular proteins. In this study, we used PROTACs in combination with proteomics to investigate the molecular basis of the S-phase function of AURORA A in an AML cell line lacking N-MYC expression. We employed the PROTAC molecules JB170 (Adhikari et al, 2020), derived from the conformational inhibitor Alisertib (Brockmann et al, 2013; Gorgun et al, 2010), and JB301 (Bozilovic et al, 2022), derived from the catalytic inhibitor MK5108 (Shimomura et al, 2010), as both induce potent, specific, and rapid depletion of AURORA A.

**Figure 6:**
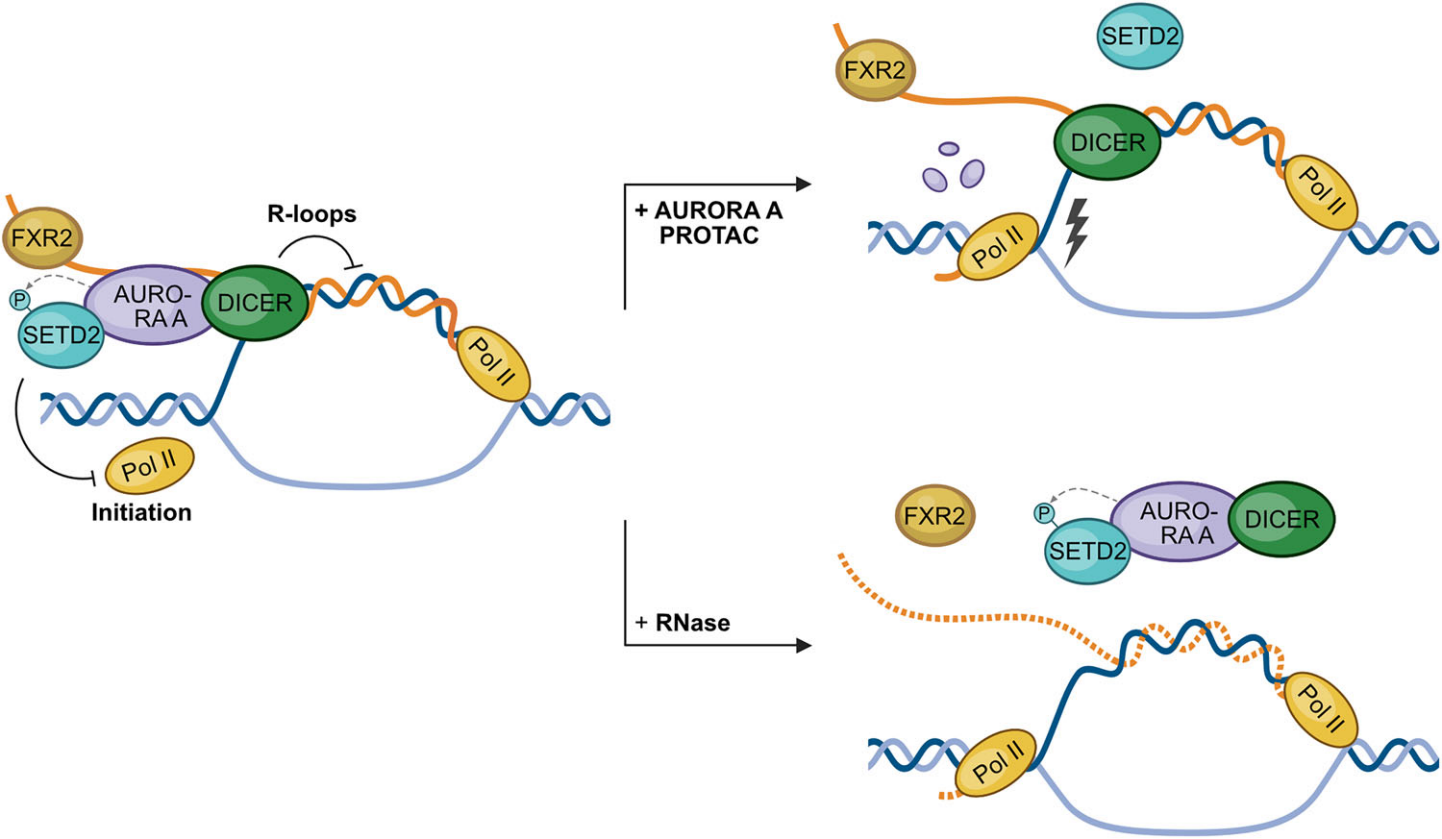
Two-output model of the AURORA A complex. Model summarizing the scaffolding function of AURORA A, DICER and SETD2 in S-phase.

Initially, we hypothesized that the scaffolding function of AURORA A in AML cells might be mediated by c-MYC, given the high structural and functional similarity between c-MYC and N-MYC (Malynn et al, 2000) and the reported interaction between AURORA A and c-MYC in hepatocellular carcinoma cells (Dauch et al, 2016). However, the AURORA A binding motif of N-MYC is not conserved in c-MYC, and we were unable to detect c-MYC in any of the AURORA A interactomes in AML cells. In contrast, we observed that S-phase AURORA A interacts with a broad range of RNA-binding proteins and RNase treatment caused a massive shift of AURORA A and many of its interactors into lighter gradient fractions (see Model in Fig. 6). Contrary to our expectations, however, RNase treatment during immunoprecipitation had little effect on the interaction of AURORA A with most of its partners. How, then, can we explain the dramatic impact of RNase treatment on the migration behavior of the heavy AURORA A complexes, given that their protein composition remains largely intact? This apparent discrepancy indicates that RNA species themselves retain AURORA A complexes within the heavy gradient fractions, or alternatively, that RNA interactions promote chromatin association of the AURORA A complex through nascent transcripts or R-loops (Fig. 6). The latter model appears particularly compelling, since DICER–one of the most enriched proteins in the S-phase interactome of AURORA A–has been increasingly implicated in nuclear functions at chromatin (Ando et al, 2011). DICER interacts with the RNA polymerase II (White et al, 2014), promotes DNA repair following genotoxic stress (Burger and Gullerova, 2018; Burger et al, 2017; Chitale and Richly, 2017; Liu et al, 2024; Tang and Ren, 2012) and contributes to the suppression of R-loops (Alagia et al, 2025; Camino et al, 2023; Cheng et al, 2025), thereby preventing the formation of stable RNA/DNA hybrids and single-stranded DNA. Recently, AURORA B silencing or inhibition was shown to produce synthetic effects in combination with DICER knockout, highlighting the intriguing possibility that the interaction with DICER is conserved across different paralogs of AURORA kinases (Gutbrod et al, 2022).

A shift in the gradient migration behavior of AURORA A after RNase treatment was recently also demonstrated in cells synchronized in mitosis (Rajagopal et al, 2025), although with slight differences, as the migration behavior of the AURORA A binding partner TPX2 is RNase-sensitive in mitosis but not in S-phase. This similarity is particularly intriguing given the pronounced differences in the interaction partners of AURORA A between S-phase and mitosis. Moreover, the same study revealed that AURORA A itself possesses RNA-binding properties, consistent with a preprint reporting similar observations in neuroblastoma cells (Müller et al, 2025). While it is likely that the numerous RNA-binding proteins we identified, such as DICER, promote the association of AURORA A with RNA in S-phase, our data are equally compatible with the notion that AURORA A can bind RNA directly. Taken together, published evidence and our own findings suggest that the association of AURORA A with RNA–whether direct or indirect–represents an evolutionarily conserved function of this kinase in both mitosis and S-phase, despite the near-complete divergence of its effector proteins in these cell cycle stages.

While RNase incubation dramatically affects RNA-binding proteins interacting with AURORA A, PROTAC-mediated depletion of AURORA A did not cause their redistribution into lighter gradient fractions. Instead, loss of AURORA A results in a shift of the E3 ligase RNF219 and, almost completely, the histone methyltransferase SETD2 (Fig. 6). We further demonstrated that chromatin binding of SETD2 was globally altered. AURORA A degradation leads to a modest increase in SETD2 occupancy at transcriptional start sites, whereas its association with transcribed genes was almost completely lost. Interestingly, a recently published study reported that SETD2 is destabilized through phosphorylation by AURORA A (Mancini et al, 2025). In agreement with these data, we observed an increase in total cellular SETD2 levels following AURORA A degradation (Fig. EV1A), which may explain its enhanced recruitment to transcriptional start sites (Fig. 6). However, the complete loss of gene-associated SETD2 upon AURORA A depletion was both unexpected and significant. These findings indicate that SETD2 recruitment to transcribed genes by AURORA A occurs despite of its destabilization. It is possible that the effect of AURORA A on SETD2 is temporally restricted, with recruitment followed by phosphorylation and subsequent destabilization.

SETD2 is the sole mammalian methyltransferase responsible for catalyzing trimethylation of lysine 36 on histone H3 (H3K36me3) and is the ortholog of yeast SET2 (Edmunds et al, 2008). Numerous studies have demonstrated that histone methylation by SETD2 is crucial for maintaining genomic integrity (Fahey and Davis, 2017; Sharda and Humphrey, 2022; Sun et al, 2020). SET2-/SETD2-dependent H3K36me3 contributes to this in several ways: (i) it prevents spurious transcription (Nojima et al, 2018; Venkatesh et al, 2012), (ii) it facilitates DNA damage repair (Kim et al, 2008; Sun et al, 2020), and (iii) it supports pre-mRNA splicing (Bhattacharya et al, 2021). Interestingly, several studies indicate that both SETD2 (Yi et al, 2017; Zhu et al, 2021) and proper levels of H3K36me3 (Pryde et al, 2009), as well as yeast Set2 (Biswas et al, 2008), are essential for the initiation and progression through S-phase. Notably, AURORA A-dependent chromatin association of SETD2 is most pronounced at genes prone to R-loop formation.

Based on this finding and in light of studies describing DICER’s association with R-loops (Alagia et al, 2025; Camino et al, 2023), we propose a dual-output model for the AURORA A S-phase complex. First, RNA-binding proteins–and possibly AURORA A itself–are recruited to R-loop–containing regions, which may arise from transcription-replication conflicts or inefficient splicing. DICER then processes the R-loop, while AURORA A recruits the effector protein SETD2. This coordinated action prevents the stressed region from being disrupted by spurious transcription, thereby facilitating efficient resolution of replicative stress. Since we did not demonstrate ternary complex formation, the proposed dual-output model does not necessarily occur simultaneously.

## Methods

**Table:** Reagents and tools table

Reagent/resource	Reference or source	Identifier or catalog number
**Experimental models**		
HEK293	ATCC	ATCC Number: CRL-1573 ^™^
MV4-11	Eilers lab	ATCC Number: CRL-9591 ^™^
U2OS	ATCC	ATCC Number: HTB-96 ^™^
**Recombinant DNA**		
	This study or Addgene	Dataset EV5
**Antibodies**		
Anti-Aurora A antibody [EPR5026]	Abcam	Cat#ab108353
Anti-BrdU-FITC antibody	BioLegend	Cat#364103
Anti-Dicer antibody [13D6]	Abcam	Cat#ab14601
Anti-FLAG M2	Sigma	Cat#F3165
Aurora A/AIK Antibody	Cell Signaling	Cat#3092S
GAPDH (14C10) Rabbit mAb	Cell Signaling	Cat#2118
HA-Tag (C29F4)	Cell Signaling	Cat#3724S
Monoclonal Anti-human Vinculin, clone*hv	Sigma	Cat#V9131
SETD2 (E4W8Q) Rabbit mAb	Cell Signaling	Cat#80290S
**Oligonucleotides and other sequence-based reagents**		
	This study	Dataset EV5
**Chemicals, enzymes and other reagents**		
Duolink® In Situ Detection Reagents Far-Red	Sigma-Aldrich	Cat#DUO92013
Duolink® In Situ PLA® Anti-Mouse MINUS	Sigma-Aldrich	Cat#DUO92004
Duolink® In Situ PLA® Anti-Rabbit PLUS	Sigma-Aldrich	Cat#DUO92002
Nano-Glo® HiBiT Lytic Detection System	Promega	Cat#N3030
NEBNext Ultra II DNA Library Prep Kit	New England Biolabs	Cat#E7645S
Pierc Anti-HA Magnetic Beads	Thermo Fisher Scientific	Cat#88837
Ambion RNase III	Thermo Fisher Scientific	Cat#AM2290
RNase A	Roth	Cat#7156.1
RNase H	New England Biolabs	Cat#M0297L
RNase I	Thermo Fisher Scientific	Cat#EN0601
RNase T1	Thermo Fisher Scientific	Cat#EN0541
**Software**		
AlphaFold 2.3.1/ 2.3.2/ 3	https://alphafoldserver.com/about, (Abramson et al, 2024; Yang et al, 2023)	
CellProfiler 4.2.8	https://cellprofiler.org/releases	
BioRender	Science Suite Inc.	
FlowJo 10	Becton, Dickinson and Company	
GraphPad Prism 10 for Mac/Windows	GraphPad Software Inc.	
Integrated Genome Browser 10.1.0	https://www.bioviz.org/	
LAS X Office	Leica	
MaxQuant version 1.6.2.2	(Cox and Mann, 2008)	
R v4.5.0	https://www.r-project.org/	
R-loopBase	10.1093/nar/gkab1103	
**Other**		
Optima XPN-80 Ultracentrifuge	Beckman Coulter	Cat#A95765

### Cell culture

Human MV4-11 cells (ATCC, CRL-9591) were cultured in RPMI 1640 medium (Thermo Fisher Scientific) supplemented with 10% FBS (Capricorn Scientific) and 1% penicillin/streptomycin (Sigma). Human HEK293TN (ATCC, Cat#CRT-11268) and U2OS cells (ATCC, HTB-96) were grown in DMEM (Thermo Fisher Scientific) supplemented with 10% FBS (Capricorn Scientific) and 1% penicillin/streptomycin (Sigma). Cells were cultured at 37 °C in 5% CO_2_ and routinely screened for mycoplasma contamination using a PCR-based assay, and the results were negative.

### Cell line generation and HiBit assay

Stable cell lines were generated by lentiviral transduction. HEK293 cells were transfected with plasmids coding for AURORA A-HiBiT, HA-tagged AURORA A, HA-tagged Cereblon or shDICER in combination with the lentiviral packaging plasmids psPAX2 (Addgene #12260) and pMD2.G (Addgene #12259) using polyethylenimine. The virus-containing media was filtered (0.45 µm) and used to infect MV4-11 cells twice every 24 h. Selection was performed 72 h after infection with 2 µg/ml puromycin (InvivoGen) or with 500 µg/ml hygromycin (InvivoGen).

To quantify PROTAC-mediated degradation of AURORA A in MV4-11 cells, HiBiT assays were performed. MV4-11 cells stably expressing C-terminally HiBiT-tagged AURORA A were incubated with the indicated PROTACs for 6 h. Cells were subsequently lysed, and luminescence was measured (Tecan Sparck) following complementation with the LgBiT luciferase fragment (Promega), as described previously (Adhikari et al, 2020).

### Generation of DICER knock-out cells

Single guide RNA (sgRNA; sequence: GCAGTTAGTCCCAAAATGCG) targeting DICER was selected from the Brunello CRISPRko library (Sanson et al, 2018) and cloned into the lentiCRISPR v2 vector (Addgene #52961) following standard protocols. Briefly, the vector was digested with BsmBI restriction enzyme, dephosphorylated, and gel-purified prior to ligation with annealed and phosphorylated sgRNA oligonucleotides. Recombinant plasmids were verified by Sanger sequencing and used for lentiviral transduction of MV4-11 and U2OS cells. Transduced cells were selected with 2 µg/mL puromycin, and DICER knockout was confirmed by Western blot analysis.

### General cloning

All vector backbones, template vectors used for PCR, eBlocks and primers are listed in Dataset EV5. HA-tagged AURORA A, HA-tagged Cerbelon and FLAG-tagged DICER were cloned by PCR amplification and inserted into pRRL using AgeI/SpeI restriction sites. AURORA A mutants were ordered as eBlocks (IDT) and inserted into a pRRL vector using AgeI/NsiI restriction sites and Gibson assembly. Point mutations in FLAG-DICER (L1094A, F1096A, and W1098A) were generated by overlapping PCR followed by Gibson assembly. A deletion in DICER (ΔL1058–R1288) was generated as well, with overlapping PCR followed by Gibson assembly. The deleted region was replaced with a GSSG-linker. shRNAs were selected based on published data (Fellmann et al, 2013; Pelossof et al, 2017) and inserted into a pLT3GEPIR vector backbone (Addgene #111177) using EcoRI and Xho1 restriction sites.

### Synchronization in S-phase

MV4-11 cells were synchronized in S-phase using an excess of thymidine. Cells were treated with 2 mM thymidine for 16 h, released for 8 h by renewing the media, and again treated with 2 mM thymidine for 16 h. MV4-11 cells were released for 1 h, followed by harvesting for immunoprecipitation.

### HA immunoprecipitation

For HA immunoprecipitation (IP) HEK293 cells were transfected with the plasmids of interest. Transfection was performed using in total 20 µg of plasmid. HA-AURORA A and FLAG-DICER WT, as well as mutants, were transfected in a 1:5 ratio (4 µg AURORA A plasmid, 16 µg DICER plasmid). Forty-eight hours post-transfection, cells were washed once with cold PBS and harvested in immunoprecipitation buffer (20 mM HEPES pH 7.9, 200 mM NaCl, 0.5 mM EDTA, 10% glycerol, and 0.2% NP-40) supplemented with phosphatase and protease inhibitors (Sigma). Lysis was performed for 30 min at 4 °C head-over-tail, followed by centrifugation at 16,000×*g* for 10 min at 4 °C. For immunoprecipitation, HA-coupled magnetic beads (Pierce Thermo Fisher Scientific) were washed with IP buffer, mixed with lysate supernatant and incubated for 3 h at 4 °C. Beads were washed four times with IP buffer before elution in 40 μl 1× lithium dodecyl sulfate (LDS) buffer (NuPAGE Thermo Fisher Scientific) by incubating at 37 °C with shaking for 30 min. The eluate was supplemented with 50 mM DTT and heated at 95 °C for 5 min. For further analysis, the eluate was loaded on an immunoblot.

### Immunoblotting

For total cell lysis, cells were lysed in RIPA buffer (50 mM HEPES, pH 7.9, 140 mM NaCl, 1 mM EDTA, 1% Triton X-100, 0.1% SDS, and 0.1% sodium deoxycholate) supplemented with phosphatase and protease inhibitors (Sigma-Aldrich) for 30 min at 4 °C head-over-tail. After centrifugation at 18,000×*g* for 10 min at 4 °C, the protein concentration of the supernatant was determined using a bicinchoninic acid (BCA) assay. Equal amounts of protein were separated by Bis-Tris-PAGE and transferred to PVDF membranes (Millipore). The membranes were blocked with 5% (w/v) non-fat dry milk in TBS-T (20 mM Tris-HCl, pH 7.5, 150 mM NaCl, 0.1% (v/v) Tween 20) for 1 h at room temperature and washed with TBS-T before incubation with primary antibodies overnight at 4 °C. The following primary antibodies were used: AURORA A: Cell Signaling, Cat# 3092S; Vinculin: RRID:AB_477629; N-MYC: RRID:AB_831602; c-MYC: RRID:AB_731658; DICER: RRID:AB_443067; GAPDH: RRID:AB_561053; HA: Cell Signaling, Cat#3724S; FLAG: RRID:AB_259529; SETD2: Cell Signaling, Cat# 80290S, and ACTB: RRID:AB_476744. For visualization, horseradish peroxidase (HRP)-labeled secondary antibodies were used and detected with chemiluminescent HRP substrate (Millipore) in the FUSION FX (Vilber) or the ImageQuant 800 (Cytiva). The signal was quantified with Image Studio Lite (LI-COR Biosciences, v6.0.0.28).

### Flow cytometry

To analyze the cell cycle distribution, BrdU-PI flow cytometry was performed. First, MV4-11 cells were labeled with BrdU (Sigma-Aldrich) for 1 h, followed by pelleting. The cell pellet was washed with ice-cold PBS, and cells were fixed in 80% ethanol overnight at −20 °C. After washing again with cold PBS, the cell pellet was resuspended in 2 M HCl, 0.5% Triton X-100 for 30 min at room temperature. For neutralization, 0.1 M Na_2_B_4_O_7_ (pH 8.5) was used, and cell pellets were resuspended in 100 µl 1% BSA in PBS-T (0.5% Tween 20 in PBS) containing anti-BrdU-FITC antibody (RRID:AB_2564480). After incubation for 30 min at room temperature in the dark, cells were washed with 1% BSA in PBS-T and resuspended in PBS with RNase A (24 μg ml^−1^) and PI (54 μM) and incubated for 30 min at 37 °C. Flow cytometry was performed on a BD Symphony or MACSQuant Analyzer 16 flow cytometer, and data were analyzed using FlowJo 10. Sample sizes for FACS and all subsequent experiments were determined in accordance with established standards in the field. The sample identity was not blinded.

### Cell growth assay

To assess cell growth, alamarBlue assays were performed. MV4-11 cells were seeded (9000 cells/well) in 96-well plates and treated with JB170, JB301 or DMSO as a control for 72 h. The alamarBlue assay (Thermo Fisher Scientific) was conducted using HS Cell Viability reagent. The fluorescence was measured on a Tecan Spark plate reader with an excitation wavelength of 550 nm and an emission wavelength of 600 nm.

### Immunoprecipitation mass spectrometry

MV4-11 cells stably expressing either an empty vector or HA-tagged AURORA A (C- and N-tag) were washed twice with cold PBS and resuspended in 4 ml MS IP lysis buffer (20 mM HEPES, pH 7.9, 180 mM NaCl, 1.5 mM MgCl_2_, 10% glycerol, 0.2% NP-40) supplemented with protease and kinase inhibitors. Per condition, 192,000,000 cells were used. Lysis was performed by homogenizing the cells with a glass homogenizer in addition to sonification (20% amplitude, 40 s time, 10 s pulse, 45 s pause). To analyze the RNA-dependency of AURORA A interactions, cell lysates were treated with an RNase mix (RNase treatment: RNase A (Roth), RNase 1, RNase T1, RNase 3 (Thermo Fisher Scientific): 4.5 µl each; RNase H (New England Biolabs): 8 µl) or buffer as a control and incubated for 40 min in a rotating wheel at 4 °C. Insolubilized chromatin was pelleted by centrifugation at 26,515×*g* for 30 min at 4 °C. The supernatant was used for immunoprecipitation with 80 µl HA-coupled magnetic beads, which were prewashed three times with IP lysis buffer. The immunoprecipitation was carried out for 3 h at 4 °C. The beads were washed 4x with MS IP lysis buffer supplemented with 0.1% Triton X-100 and twice with MS IP lysis buffer only. After washing, the proteins were eluted by incubation at 37 °C for 30 min in 100 µL 1x LDS Sample buffer (NuPAGE Thermo Fisher Scientific). The eluted proteins were reduced with 50 mM DTT and denatured by heating for 5 min at 95 °C. Afterward, proteins were precipitated overnight at −20 °C with fourfold volume of acetone. Pellets were washed three times with acetone at −20 °C. Precipitated proteins were dissolved in NuPAGE® LDS sample buffer (Life Technologies), reduced with 50 mM DTT at 70 °C for 10 min and alkylated with 120 mM Iodoacetamide at room temperature for 20 min. Separation was performed on NuPAGE® Novex® 4-12% Bis-Tris gels (Life Technologies) with MOPS buffer according to the manufacturer’s instructions. Gels were washed three times for 5 min with water and stained for 60 min with Simply Blue™ Safe Stain (Life Technologies). After washing with water for 1 h, each gel lane was cut into 15 slices. The excised gel bands were destained with 30% acetonitrile in 0.1 M NH4HCO3 (pH 8), shrunk with 100% acetonitrile, and dried in a vacuum concentrator (Concentrator 5301, Eppendorf, Germany). Digests were performed with 0.1 µg trypsin per gel band overnight at 37 °C in 0.1 M NH4HCO3 (pH 8). After removing the supernatant, peptides were extracted from the gel slices with 5% formic acid, and the extracted peptides were pooled with the supernatant. NanoLC-MS/MS analyses were performed on an Orbitrap Fusion (Thermo Scientific) equipped with a PicoView Ion Source (New Objective) and coupled to an EASY-nLC 1000 (Thermo Scientific). Peptides were loaded on a trapping column (2 cm × 150 µm ID, PepSep) and separated on a capillary column (30 cm × 150 µm ID, PepSep), both packed with 1.9-µm C18 ReproSil and separated with a 30-min linear gradient from 3 to 30% acetonitrile and 0.1% formic acid and a flow rate of 500 nl/min. Both MS and MS/MS scans were acquired in the Orbitrap analyzer with a resolution of 60,000 for MS scans and 30,000 for MS/MS scans. HCD fragmentation with 35% normalized collision energy was applied. A Top Speed data-dependent MS/MS method with a fixed cycle time of 3 s was used. Dynamic exclusion was applied with a repeat count of 1 and an exclusion duration of 90 s; singly charged precursors were excluded from selection. The minimum signal threshold for precursor selection was set to 50,000. Predictive AGC was used with AGC as a target value of 4 × 105 for MS scans and 5 × 104 for MS/MS scans. EASY-IC was used for internal calibration. Raw MS data files were analyzed with MaxQuant version 1.6.2.2 (Cox and Mann, 2008). Database search was performed with Andromeda, which is integrated in the utilized version of MaxQuant. The search was performed against the UniProt Human Reference Proteome database (Release 2023_05, UP000005640, 82685 entries). Additionally, a database containing common contaminants was used. The search was performed with tryptic cleavage specificity with three allowed miscleavages. Protein identification was under control of the false discovery rate (FDR; <1% FDR on protein and peptide-spectrum match (PSM) level). In addition to MaxQuant default settings, the search was performed against the following variable modifications: Protein N-terminal acetylation, Gln to pyro-Glu formation (N-term. Gln) and oxidation (Met). Carbamidomethyl (Cys) was set as a fixed modification. Further data analysis was performed using R scripts developed in-house. LFQ intensities were used for protein quantitation (Cox et al, 2014). Proteins with less than two razor/unique peptides were removed. Missing LFQ intensities were imputed with values close to the baseline. Data imputation was performed with values from a standard normal distribution with a mean of the 5% quantile of the combined log10-transformed LFQ intensities and a standard deviation of 0.1. The enrichment was calculated based on LFQ intensities, and *p* values were calculated in the limma package in R using the linear method.

### AlphaFold modeling

AlphaFold predictions were performed using AlphaFold 2.31, AlphaFold 2.3.2 or AlphaFold 3 (Abramson et al, 2024; Yang et al, 2023). Predictions with AlphaFold 2 were performed with a reduced_dbs dataset due to a bug in the database. As input for AURORA A, the catalytic subunit was used (122–403 aa) due to difficulties in full-length AURORA A structure prediction. The quality of prediction was assessed using the per-residue measure of local confidence (pLDDT) score. Only amino acids with a pLDDT above 50 were taken into account. The region with possible high interaction (defined as amino acids from DICER and AURORA A in close proximity (≤5 Å)) was further screened by different truncated sequences. For DICER mutants, a deletion mutant was designed (Δ L1058–R1288) where the predicted AURORA A interaction region was replaced by a GSSG-linker. For AURORA A mutants, the amino acids in the F, Y and W-pocket were switched from hydrophobic to polar or vice versa. It was taken care that the total charge of the pocket did not change (F-pocket: W128E, I209R, Y197R, F157E, Y-pocket: L169R, L178R, V182E, W-pocket: I184R, H187E, V252E, and L188R).

### Immunofluorescence

For immunofluorescence, U2OS cells were seeded in 96-well plates and fixed with 4% paraformaldehyde in PBS, followed by permeabilization with 0.2% Triton X-100 for 15 min at room temperature. For cell cycle-resolved immunofluorescence, cells were labeled with 20 μM EdU for 2 h prior to fixation. Blocking was performed with 3% BSA in PBS for 30 min at room temperature, and fixed cells were incubated with primary antibody (AURORA A: RRID:AB_10865712; DICER: RRID:AB_443067) diluted in 3% BSA in PBS overnight at 4 °C. After washing with PBS, cells were incubated with fluorescent-labeled secondary antibody (RRID:AB_143165 and RRID:AB_2534069) in 3% BSA in PBS for 1 h at room temperature in the dark. Nuclei were counterstained using 2.5 µg/ml Hoechst 33342 solution (Thermo Fisher Scientific) for 5 min. The remaining counterstain solution was removed by washing twice with PBS. Fluorescence image acquisition was performed with the Mica microscope (Leica) and a 10x or 20x objective.

### PLA assay and quantification

U2OS cells were seeded in 96-well plates and fixed with 4% paraformaldehyde in PBS, followed by permeabilization with 0.1% Triton X-100 for 15 min at room temperature. For cell cycle-resolved PLA, cells were labeled with 20 μM EdU for 2 h prior to fixation. Blocking was performed in 1% BSA in PBS-T (PBS + 0.1% Tween 20) for 30 min at room temperature. Primary antibodies (AURORA A: RRID:AB_10865712; DICER: RRID:AB_443067) were diluted in blocking buffer and incubated overnight at 4 °C. The PLA was conducted using the Duolink® In Situ Kit (Sigma-Aldrich) according to the manufacturer´s protocol. Briefly, fixed cells were incubated with PLA probe-labeled anti-rabbit and anti-mouse IgG for 1 h at 37 °C. Next, ligation was performed for 30 min at 37 °C, followed by PCR amplification using Far-red-conjugated oligonucleotides for 1 h at 37 °C. Nuclei were stained with Hoechst 33342 (Thermo Fisher Scientific), and cytoplasm was stained using Alexa 568-conjugated phalloidin (Thermo Fisher Scientific, Cat#A12379). Fluorescence image acquisition was performed with the Mica microscope (Leica) and a 10x or 20x objective. Data analysis was performed with CellProfiler 4.2.8. The integrated intensity of all PLA spots was measured and summarized for every whole cell, only the cytoplasm or only the nucleus of a cell. Only signals with even or higher summarized integrated intensity of 25 were accepted as positive. The signal was normalized to the total number of whole cells / cytoplasm or nuclei of every field of view (FoV) to get the percentage of PLA-positive cells. Three independent experiments and three to four different FoVs were analyzed. Statistical analysis of the PLA assay was performed by Graphpad Prism 10.5.0. For the cell cycle-resolved PLA analysis, images were analyzed with CellProfiler to obtain per-nucleus values for PLA foci count, EdU intensity and Hoechst integrated intensity. The distributions of EdU intensity and Hoechst integrated intensity were used for the readout of DNA synthesis and DNA content, respectively and used for the determination of cell cycle phases.

### Cleavage under targets and release using nuclease sequencing (CUT&RUN-seq)

Cleavage under targets and release using nuclease (CUT&RUN) followed by sequencing was conducted as previously described (Meers et al, 2019). MV4-11 cells (4 × 10^6^ per condition) were treated with 150 nM JB301 or DMSO for 6 h, followed by 3x washing with wash buffer (20 mM HEPES, pH 7.5, 150 mM NaCl, 0.5 mM Spermidine). Concanavalin A-coated magnetic beads (Polysciences Europe) were prewashed with binding buffer (20 mM HEPES, pH 7.5, 10 mM KCl, 1 mM CaCl_2_, 1 mM MnCl_2_) and 20 µl beads per condition were incubated with cell suspension on a rotating wheel for 10 min at room temperature. For permeabilization, the supernatant was removed, and 150 µl Dig-wash buffer (wash buffer supplemented with 0.05% Digitonin and containing 2 mM EDTA) was added to the beads. For R-loop removal, the samples were supplemented with 5 µl RNase H (NEB) each and incubated for 30 min at 37 °C. The beads were then incubated with SETD2 antibody (Cell Signaling, Cat# 80290S) overnight at 4 °C while shaking (800 rpm). Next, cells were washed 2x with Dig-wash buffer and incubated with Protein A/G-MNase fusion protein (1 μg/ml in 150 μl Dig-Wash buffer) for 1 h at 4 °C while shaking (800 rpm). Beads were washed twice with Dig-wash buffer and once with low salt buffer (20 mM HEPES (pH 7.5), 0.05% Digitonin, and 0.5 mM Spermidine). MNase was activated by the addition of 200 µl ice-cold incubation buffer (3.5 mM HEPES (pH 7.5), 10 mM CaCl_2_, and 0.05% Digitonin), and incubation was performed for 30 min at 0 °C. The reaction was stopped by the addition of 200 µl STOP buffer (170 mM NaCl, 20 mM EGTA, 0.05% Digitonin, 50 μg/ml RNase A, and 25 μg/ml Glycogen) followed by incubation at 37 °C for 30 min to release the MNase-cleaved DNA fragments from the insoluble nuclear chromatin. Protein digestion was carried out by adding 0.1% SDS and 250 µg/ml proteinase K and incubating at 50 °C for 1 h. DNA was isolated with phenol-chloroform extraction and quantified using PicoGreen and the TapeStation 4150 (Agilent Technologies). The input material was equalized, and libraries were prepared using the NEBNext Ultra II DNA Library Prep Kit for Illumina Sequencing with PCR amplification of 16–18 cycles.

The sequencing was performed by BGI Genomics on a DNBSEQ G400 instrument (100 + 100, paired-end). For analysis, the Illumina adapters were trimmed using CutAdapt V1.18 (Kechin et al, 2017), and the FASTQ files were aligned against the GRCh38 genome using Bowtie2 V2.2.45 (Langmead and Salzberg, 2012). BAM files were sorted and indexed using Samtools 1.6 (Danecek et al, 2021), bedgraph files were generated and scaled to equal sequencing depth using bedtools V2.26.0 (Quinlan, 2014). Replicate 3 of the control- treated MV4-11 cells was excluded from all analyses since it behaved like the IgG control and was significantly different from replicates one and two. Metagene plots were created with deeptools 3.5.4. (Ramirez et al, 2016) using bamCoverage, computeMatrix and plotProfile. To analyze the read coverage in the gene body or in the promoter, the gene body was defined as transcription start site (TSS) + 1 kb until the transcription end site (TES) and the promoter was defined as TSS ± 1 kb. Reads in promoter and gene body regions were counted using FeatureCounts v2.0.6 (Liao et al, 2014). For the volcano plot of SETD2 gene body binding, log_2_ fold-changes and *p* values were calculated using DESeq2 using sizeFactors to normalize to sequencing depth (Love et al, 2014) and plotted with ggplot2 in R. Human R-loop positions were downloaded from the R-loopBase (Lin et al, 2022). Human genes expressed in MV4-11 cells were intersected with level 4 R-loop regions and classified as genes with R-loop (≥1) or without R-loop (=0). For boxplots and scatter plots, SETD2 read counts were normalized to region length. Reads in the IgG control in these areas were subtracted, followed by flooring to 0. Finally, the mean coverage of replicates was calculated and plotted for genes with or without R-loop using ggplot2 in R. The promoter ratio was calculated by dividing the mean normalized read count in the promoter of a gene by its gene body read count.

### RNA extraction and analysis

For total RNA extraction 10 µl MV4-11 cell lysate was mixed properly with 0.5 ml trizol (Invitrogen) by pipetting up and down 10x. The cell lysate was triturated 6x with a 0.6 mm syringe to dislodge the RNA polymerase and incubated for 5 min at room temperature. About 100 µl chloroform was mixed with the cell lysate and centrifuged at 12,000×*g* for 10 min at 4 °C. The upper clear phase was collected, and 1 µl glycoblue (Invitrogen) was added to ensure the visibility of the pellet after centrifugation. For precipitation, 250 µL ice-cold isopropanol was added, mixed and incubated for 5 min at –20 °C. The RNA was pelleted by centrifugation at 20,000×*g* for 10 min at 4 °C and washed 2x with cold 75% ethanol. Remaining ethanol was removed by air-drying, and the RNA pellet was resuspended in 8 µl of nuclease-free water. The concentration of the RNA was determined by UV/VIS spectrophotometry, and 200 ng RNA were further analyzed using the fragment analyzer (Agilent).

### Gradient centrifugation

Gradient centrifugation (GC) was adapted from previously described protocols (Caudron-Herger et al, 2020). MV4-11 cells were counted using a Neubauer chamber. Based on the determined cell number, GC lysis buffer (25 mM Tris-HCl, pH 7.4, 150 mM KCl, 0.5% (v/v) NP-40, 2 mM EDTA, 1 mM NaF, 0.5 mM DTT) supplemented with protease and kinase inhibitors was added (100 µl lysis buffer per 2 × 10^7^ cells). The cell suspension was incubated on ice for 30 min with resuspension by vortexing every 5 min. To completely lyse the cells, the cell suspension was 2x frozen in liquid nitrogen and homogenized using a thin needle (0.6 × 30 mm). The suspension was centrifuged at 16,000×*g* for 10 min at 4 °C, and the protein concentration of the supernatant was determined using a BCA assay. The cell lysates were diluted to 24 mg/ml with GC lysis buffer and treated optionally with a RNase cocktail (RNase treatment: RNase A (Roth), RNase 1, RNase T1, and RNase 3 (Thermo Fisher Scientific): 2.5 µl each; RNase H (New England Biolabs): 5 µl) for 1 h in a rotating wheel at 4 °C. Next, 6 mg protein was loaded on a 5–50% sucrose gradient, which was prepared using the gradient master 108 (Biocomp). Ultracentrifugation was performed at 30,000 rpm (SW41 rotor) for 18 h at 4 °C followed by fractionation in 24 fractions (500 µl each) from top to bottom. For protein precipitation, 50 µl of 0.15% DOC was added to each fraction and incubated for 10 min at room temperature. About 25 µl of 100% TCA was added, followed by incubation for 30 min on ice. Proteins were pelleted by centrifugation at 16000×*g* for 15 min at 4 °C. The pellet was washed once with 500 µl ice-cold acetone and dried at room temperature. Protein pellets were either analyzed by mass spectroscopy or resuspended in 40 µl laemmli buffer (4% (w/v) SDS, 0.02% (w/v) bromophenol blue, 13.3% (v/v) glycerol, 20 mM Tris-HCl, pH 6.8, 3.1% (w/v) DTT) and loaded on an SDS gel.

For mass spectroscopy analysis, the protein precipitates were solubilized in 2% SDS buffer (2% SDS in 40 mM Tris/HCl, pH 8), and the protein concentration was determined using the Pierce^TM^ BCA Protein Assay Kit (Thermo Scientific) according to the manufacturer’s protocol. SP3 clean-up was performed with 10 µg protein following the protocol first described in (Hughes et al, 2019), adapted to a BRACO Agilent liquid handling platform. In short, protein was mixed with 50 μg SP3 beads (50:50 mixture of Sera-Mag carboxylate-modified magnetic bead types A and B (Cytiva Europe)) in a 96-deep-well plate, and proteins were precipitated onto the beads in 70% ethanol in ddH2O (double distilled water). The beads were washed three times with 80% ethanol in ddH2O and once with 100% acetonitrile (ACN). Disulfide bonds were reduced with 10 mM DTT (Dithiothreitol) for 45 min at 37 °C, followed by alkylation of cysteines with 55 mM CAA (2-chloroacetamide) for 30 min at RT in 100 μl of digestion buffer (2 mM CaCl_2_ in 40 mM Tris-HCl, pH 7.8). Trypsin (1:50 (wt/wt) enzyme-to-protein ratio) was added, and proteins were digested off the beads at 37 °C and 1200 rpm overnight. For peptide recovery, the bead supernatant was transferred to a new 96-well plate. Beads were washed with 100 μl 2% formic acid in ddH₂O, and the wash was pooled with the supernatant.

Desalting was performed with self-packed StageTips (Rappsilber et al, 2007) using SCX material (Empore^TM^ Cation Extraction Disks, Supelco). Tips were equilibrated with 0.1% formic acid in 30% acetonitrile. After sample loading, tips were washed two times with 200 µL 0.1% formic acid in 30% acetonitrile. Peptides were eluted with 100 µL 4% ammonium formate (pH 6.5) in 50% acetonitrile. Finally, all StageTip eluates were lyophilized in a SpeedVac for LC-MS/MS analysis.

Data from gradient fractions of AURKA-depleted lysates and controls (PROTAC/DMSO/RNase) were acquired by microflow LC coupled via a VIP HESI source to a timsTOF HT mass spectrometer (Bruker) as described previously (Tushaus et al, 2023). Separation was performed on a Vanquish Neo UHPLC system (Thermo Fisher Scientific) using a PepMap C18 column (1 mm ID, 15 cm, 2 µm) at 50 µl/min. Samples (1 µg) were reconstituted in 0.1% FA/2% ACN and separated with a 15 min gradient (3.5–35% B; A: 0.1% FA in H₂O, B: 80% ACN/0.1% FA, +3% DMSO) at 60 °C.

The mass spectrometer ran in data-dependent PASEF mode (mobility range 0.85–1.3, 100 ms ramp time, 100% duty cycle) with collision energies of 59 eV at 1/K0 of 1.6 Vs/cm^2^ to 29 at 1/K0 of 0.6 Vs/cm^2^, target intensity 12,000, and threshold 1600. MS2 acquisition was accelerated (quadrupole switching 1.2 ms; MS2 acquisition time 2 ms).

Gradient fractions from AURKA-inhibited samples and controls were analyzed using a Vanquish Neo UHPLC system (Thermo Fisher Scientific) coupled via a Nanospray Flex source to an Orbitrap Astral mass spectrometer (Thermo Fisher Scientific). Samples were reconstituted in 0.1% FA/2% ACN, and 250 ng of peptide was analyzed. Peptides were loaded onto a PepMap Neo Trap Cartridge (300 µm × 5 mm) using trap-and-elute mode and separated on an Ionopticks Aurora Elite column (75 µm ×  15 cm) at 50 °C using a 22-min gradient (2–32% B; A: 0.1% FA in H₂O, B: 0.1% FA in ACN) at 300–750 nL/min, followed by washing (80% B) and re-equilibration. MS analysis was performed in positive mode using DDA. MS1 scans were acquired in the Orbitrap (240,000 resolution, 360–1300 m/z, 0.6 s cycle, AGC 300%, 3 ms IT), while MS/MS spectra were recorded in the Astral analyzer (NCE 26%, 1.2 Th isolation, AGC 300%, 5 ms IT, 150–2000 m/z). Precursors (charge 2–6 and unknown) were selected with MIPS enabled, and dynamic exclusion was set to 5 s (5 ppm mass tolerance).

For gradient fractions measured on the timsTOF HT mass spectrometer, protein and peptide identification and quantification was performed using MaxQuant (Cox and Mann, 2008) (version 2.4.2.0) by searching the MS2 data against all canonical protein sequences, including isoforms as annotated in the UniProt reference database (human proteins only, downloaded 24.08.2020) using the search engine Andromeda (Cox et al, 2011). Carbamidomethylated cysteine was set as a fixed modification; oxidation of methionine and N-terminal protein acetylation were set as variable modifications. Trypsin/P was specified as a proteolytic enzyme, and up to two missed cleavage sites were allowed. All data were adjusted to 1% peptide-spectrum match (PSM) and 1% protein false discovery rate (FDR). LFQ-based quantification was enabled.

For gradient fractions measured on the Orbitrap Astral mass spectrometer, as well as for the re-analysis of samples from AURKA-depleted lysates, Fragpipe (v23.0 and v24.0) with its integrated search engine MSFragger (Kong et al, 2017) was used. Data were searched against all canonical protein sequences as annotated in the UniProt reference database (human proteins only, downloaded 30.07.2025 and extended by a list of common contaminants (Frankenfield et al, 2022). The “LFQ-MBR” workflow was used, the data type was set to “DDA + ” (Yu et al, 2025) and trypsin was selected as the enzyme. In the IonQuant (Yu et al, 2021) tab, normalization was disabled. All other options were kept as the default.

Data analysis were performed using R (version 3.6.3/4.5.0) in RStudio on identified and quantified protein groups as provided in the proteinGroups.txt or combined_protein.tsv file, respectively. Proteingroups.txt was filtered for contaminants and reverse hits, and median centric normalization and log2 transformation were performed. combined_protein.tsv was filtered for contaminants, and median centric normalization and log2 transformation were performed.

Data analysis were performed using R v4.5.0 and RStudio 2024.12.1 + 563 (Caudron-Herger et al, 2019). LFQ intensities were normalized between triplicates by calculating a normalization factor as the mean of the two most similar replicates. Next, a sliding window/moving average of three points was applied, and the average of three replicates was calculated (except for the PROTAC condition, where cells were treated with three different PROTAC concentrations before cell lysis). Every condition was normalized to 100 to account for different detection levels in different conditions. The data were plotted using GraphPad Prism 10.

For RNA-dependency analysis, a DMSO and an RNase score was calculated by dividing the sum of higher fractions (fractions 12–24) by the sum of lower fractions (fractions 1–11). The RNA-dependency score is defined by log2 of the quotient of the DMSO and the RNase score. The PROTAC-dependency score was calculated similarly. However, since no triplicates of the PROTAC condition were performed, the two highest PROTAC concentrations (0.6 and 150 nM JB301) were taken into account.

### shRNA-mediated silencing of DICER

For the generation of stable cell lines with a doxycycline-inducible system, MV4-11 and U2OS cells were infected with lentivirus according to the protocol described below. To acquire five different shRNA-silenced cell lines, the five sequences of shRNA were cloned into the plasmid pLT3GEPIR-GFP R96H.

All shRNA sequences were used from a prediction of potent shRNAs with a sequential classification algorithm (Pelossof et al, 2017). After selection with 2 µg/ml puromycin and recovery, cells were seeded in six-well plates and treated with doxycycline (1 µg/ml) for 3 days. Afterward, they were harvested and Western blots for efficacy of Doxycyline-inducible DICER depletion was tested. For further flow cytometry experiments, MV4-11 cells containing shDICER-4 were used and also treated with Doxycycline (1 µg/ml) for 3 days.

## Supplementary information

Peer Review File

Dataset EV1

Dataset EV2

Dataset EV3

Dataset EV4

Dataset EV5

Source data Fig. 1

Source data Fig. 2

Source data Fig. 3

Source data Fig. 4

Source data Fig. 5

Expanded View Figures

## Data Availability

The mass spectrometry proteomics data, including MaxQuant/Fragpipe search results, have been deposited to the PRoteomeXchange Consortium via the PRIDE partner repository with the dataset identifiers PXD076528 and PXD069843. Next-generation sequencing–based CUT&RUN data have been deposited in the European Nucleotide Archive (ENA) under the accession number PRJEB101043. The source data of this paper are collected in the following database record: biostudies:S-SCDT-10_1038-S44319-026-00850-0.
