# Supplementary material for: AURORA A interacts with DICER and SETD2 to promote S-phase progression
Source: EMBO Rep. 2026 Jun 30;27(15):4493–523. doi: 10.1038/s44319-026-00850-0 (PMC13458143; doi:10.1038/s44319-026-00850-0)
Supplement: Supplementary file 1 [file 44319_2026_850_MOESM12_ESM.pdf]

Expanded View Figures

Figure EV1. AURORA A interacts with RNA-binding proteins during S-phase.

(**A**) Immunoblots showing AURORA A and SETD2 levels after AURORA A degradation with the PROTACs JB170 and JB301. MV4-11 cells were treated for 6 h with either JB170 (600 nM) or JB301 (150 nM), and cell lysates were blotted. (**B**) Flow cytometry gating strategy for BrdU-PI-based cell cycle analysis. Representative gating from an exemplary experiment is shown, illustrating the sequential selection of cells used to determine cell cycle distribution. A similar gating strategy was used for all the BrdU-PI flow cytometry experiments. (**C**) Top: BrdU-PI cell cycle profile of unsynchronized MV4-11 cells without BrdU incorporation, serving as a control for Low-BrdU S-phase cells in Fig. 1D. Bottom: Quantification of BrdU-PI cell cycle distribution from three independent biological replicates. One representative replicate is displayed in Fig. 1D. (**D**) Top: Model of AURORA A and potential interactors. Below: Immunoblots showing AURORA A expression levels in MV4-11 empty vector (EV) and HA-tagged AURORA A overexpressing cells (N- and C-tag). (**E**) Enrichment plot after AURORA A PROTAC treatment. MV4-11 cells overexpressing HA-CRBN were treated with 150 nM JB301, followed by cell lysis and label-free quantification by mass spectroscopy. S-phase AURORA A interactors (Fig. 1E) are labeled in red. (**F**) RNA profile in MV4-11 HA-CRBN cell lysates treated with or without an RNase cocktail (RNase A, RNase III, RNase H, RNase I, and RNase T1). RNA was extracted from cell lysates using Trizol and loaded on a fragment analyzer. (**G**) Top: UV trace of gradient fractions. MV4-11 HA-CRBN cells were lysed and treated with or without an RNase mixture. The lysate was loaded on a 5–50% sucrose gradient, centrifuged and fractionated. Absorbance at 245 nm was measured for all precipitated fractions. Below: Gradient fractions were analyzed by label-free mass spectroscopy and normalized LFQ intensities for ribosome subunits RPL9 and RPS17 are shown. (**H**) Model: AURORA A together with direct and indirect RNA interactors.

 © The Author(s)

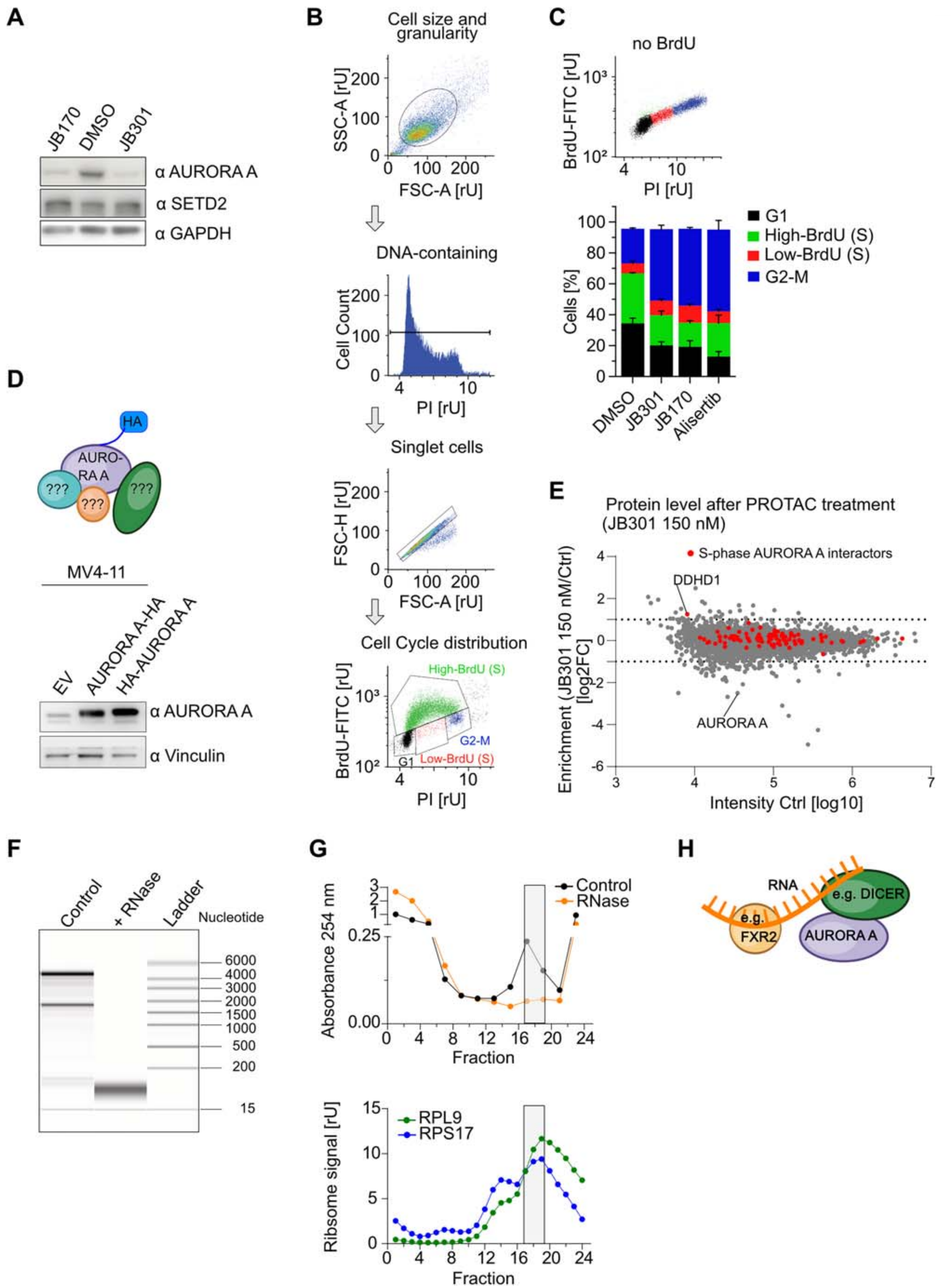
A
JB170
DMSO
JB301
α AURORA A
α SETD2
α GAPDH
B
Cell size and granularity
SSC-A [rU]
FSC-A [rU]
DNA-containing
Cell Count
PI [rU]
Singlet cells
FSC-H [rU]
FSC-A [rU]
Cell Cycle distribution
High-BrdU (S)
G2-M
G1
Low-BrdU (S)
BrdU-FITC [rU]
PI [rU]
C
no BrdU
BrdU-FITC [rU]
PI [rU]
G1
High-BrdU (S)
Low-BrdU (S)
G2-M
Cells [%]
DMSO
JB301
JB170
Alisertib
D
HA
AURORA A
???
MV4-11
EV
AURORA A-HA
HA-AURORA A
α AURORA A
α Vinculin
E
Protein level after PROTAC treatment (JB301 150 nM)
S-phase AURORA A interactors
DDHD1
AURORA A
Enrichment (JB301 150 nM/Ctrl) [log2FC]
Intensity Ctrl [log10]
F
Control
+ RNase
Ladder
Nucleotide
6000
4000
3000
2000
1500
1000
500
200
15
G
Control
RNase
Absorbance 254 nm
Fraction
RPL9
RPS17
Ribsome signal [rU]
Fraction
H
RNA
e.g. DICER
e.g. FXR2
AURORA A

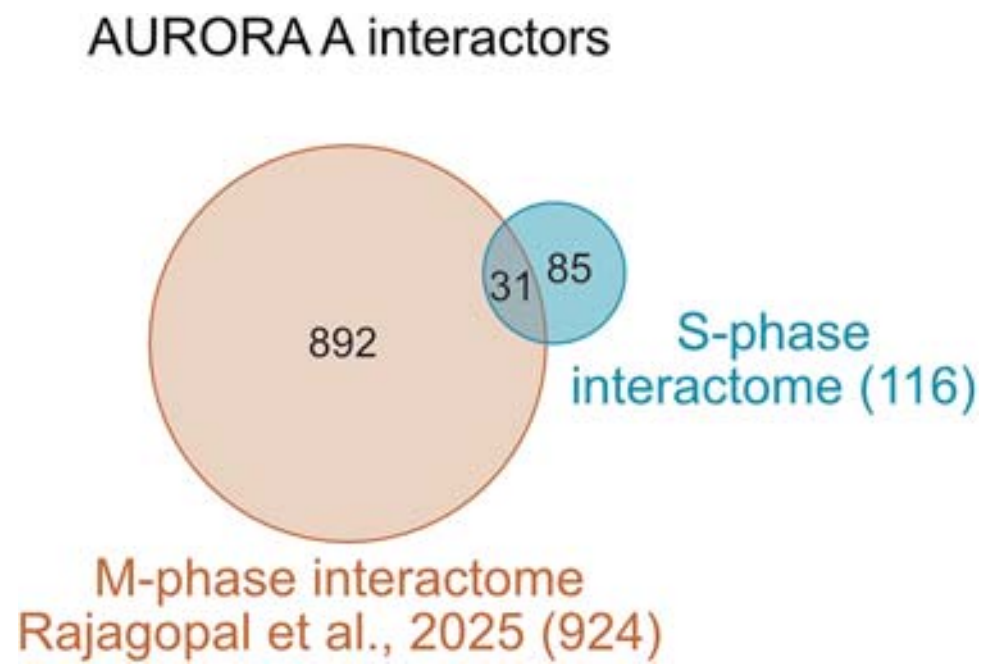

Figure EV2. Integrity of AURORA A protein complexes depends on RNA.

Overlay of the AURORA A S-phase interactome of this study with the published mitotic interactome (Rajagopal et al, 2025). Only interactors with the following cut-off are shown: enrichment HA-AURORA A versus control log2FC >3.

 © The Author(s)

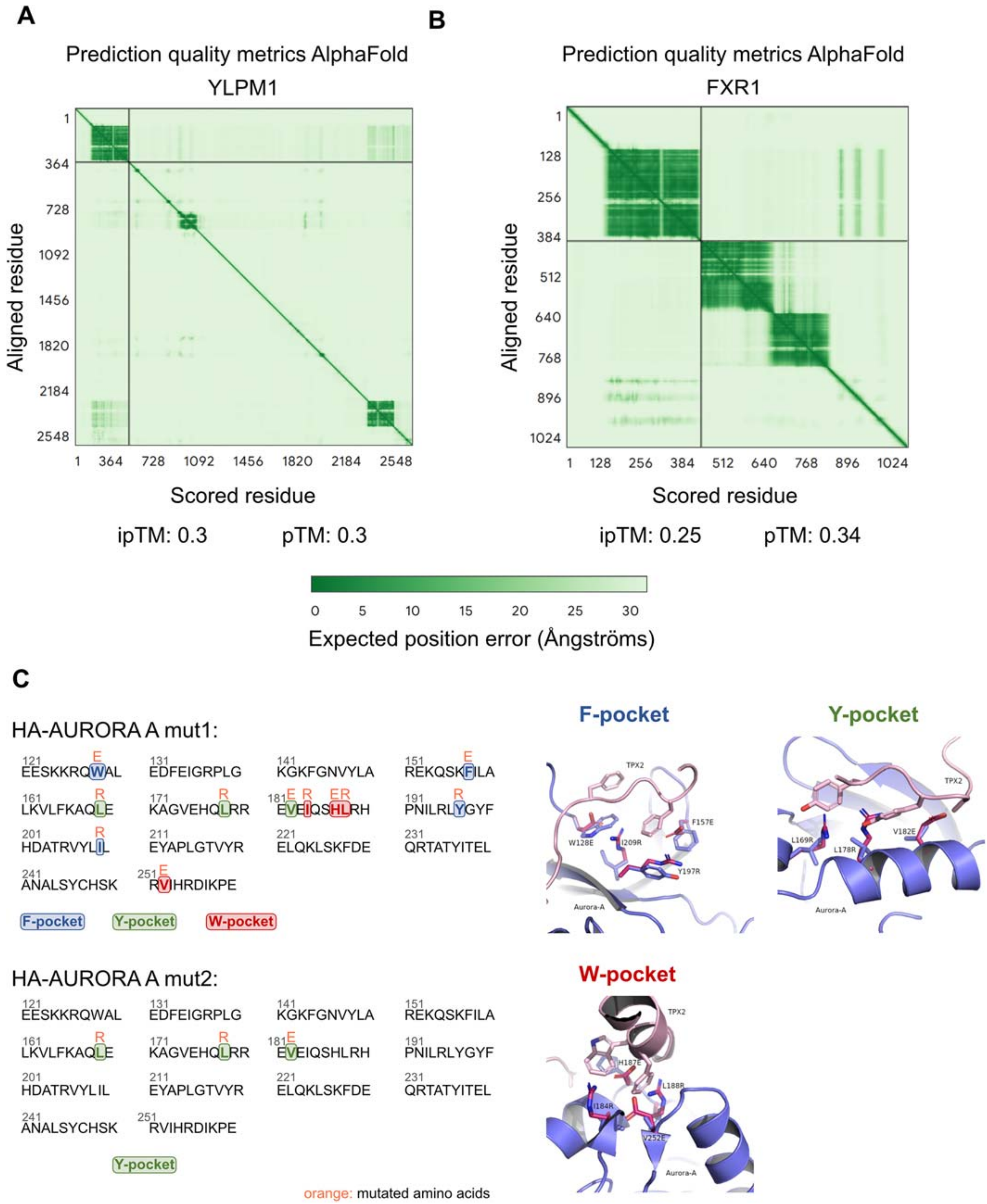

A
Prediction quality metrics AlphaFold
YLPM1
Aligned residue
Scored residue
1 364 728 1092 1456 1820 2184 2548
ipTM: 0.3 pTM: 0.3
B
Prediction quality metrics AlphaFold
FXR1
Aligned residue
Scored residue
1 128 256 384 512 640 768 896 1024
ipTM: 0.25 pTM: 0.34
0 5 10 15 20 25 30
Expected position error (Ångströms)
C
HA-AURORA A mut1:
121 EESKKRQWAL 131 EDFEIGRPLG 141 KGKFGNVYLA 151 REKQSKFILA
161 LKVLFKAQLE 171 KAGVEHQLRR 181 EVEIQSHLRH 191 PNILRLYGYF
201 HDATRVYLIL 211 EYAPLGTVYR 221 ELQKLSKFDE 231 QRTATYITEL
241 ANALSYCHSK 251 RVIHRDIKPE
F-pocket Y-pocket W-pocket
HA-AURORA A mut2:
121 EESKKRQWAL 131 EDFEIGRPLG 141 KGKFGNVYLA 151 REKQSKFILA
161 LKVLFKAQLE 171 KAGVEHQLRR 181 EVEIQSHLRH 191 PNILRLYGYF
201 HDATRVYLIL 211 EYAPLGTVYR 221 ELQKLSKFDE 231 QRTATYITEL
241 ANALSYCHSK 251 RVIHRDIKPE
Y-pocket
orange: mutated amino acids
F-pocket
TPX2
W128E
I209R
F157E
Y197R
Aurora-A
Y-pocket
TPX2
L169R
L178R
V182E
Aurora-A
W-pocket
TPX2
H187E
L188R
I184R
V252E
Aurora-A

◀ **Figure EV3. AURORA A interacts with the C-linker domain of DICER.**

(**A**) Predicted alignment error plot for the interaction of full-length AURORA A and full-length YLPM1. For predictions, AlphaFold 3 was used, and the best hit is shown here. ipTM interface predicted template modeling score, pTM predicted template modeling score. (**B**) As in (**A**), but here the prediction of the interaction of full-length AURORA A and full-length FXR1 is depicted. (**C**) Mutated amino acids in AURORA A F-, Y-, and W-pocket. Left: The WT AURORA A sequence is shown here (from amino acid 121 to 260). All amino acids which were mutated in this study are colored according to the TPX2 binding pockets. Exchanged amino acids are colored in orange. Right: Crystal structure of AURORA A with TPX2 (PDB: 1ol5) (Bayliss et al, 2003). TPX2 binding pockets of AURORA A are shown. Mutated amino acids of AURORA A are annotated.

 © The Author(s)

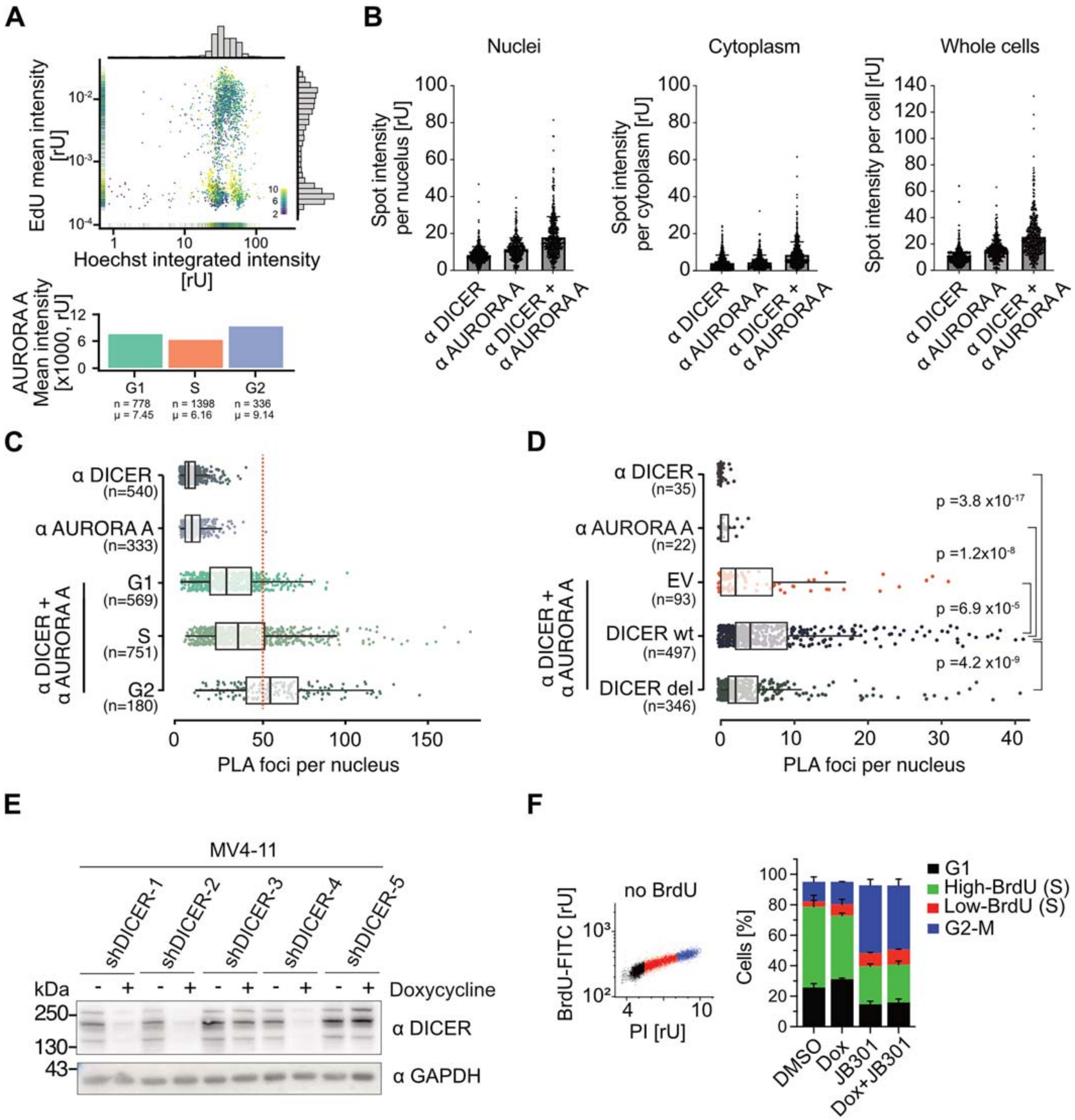
A
EdU mean intensity [rU]
Hoechst integrated intensity [rU]
AURORA A Mean intensity [x1000, rU]
G1
S
G2
n = 778
μ = 7.45
n = 1398
μ = 6.16
n = 336
μ = 9.14
B
Nuclei
Cytoplasm
Whole cells
Spot intensity per nucleus [rU]
Spot intensity per cytoplasm [rU]
Spot intensity per cell [rU]
α DICER
α AURORA A
α DICER + α AURORA A
C
α DICER (n=540)
α AURORA A (n=333)
G1 (n=569)
S (n=751)
G2 (n=180)
α DICER + α AURORA A
PLA foci per nucleus
D
α DICER (n=35)
α AURORA A (n=22)
EV (n=93)
DICER wt (n=497)
DICER del (n=346)
p =3.8 x10-17
p =1.2x10-8
p =6.9 x10-5
p =4.2 x10-9
PLA foci per nucleus
E
MV4-11
shDICER-1
shDICER-2
shDICER-3
shDICER-4
shDICER-5
kDa
Doxycycline
α DICER
α GAPDH
F
no BrdU
BrdU-FITC [rU]
PI [rU]
Cells [%]
G1
High-BrdU (S)
Low-BrdU (S)
G2-M
DMSO
Dox
JB301
Dox+JB301

Figure EV4. AURORA A and DICER are in close cellular proximity.

(**A**) Cell cycle-resolved analysis of AURORA A immunofluorescence in U2OS cells. Scatter plot (top panel) shows individual nuclei ($n = 2512$) as dots plotted according to DNA content (Hoechst integrated intensity, log scale) and DNA synthesis (EdU mean intensity, log scale) on the x- and y-axis, respectively, enabling cell cycle assignment. Dots were colored according to the AURORA A immunofluorescence intensity per nucleus (x 1000 a.U.). The color scale of AURORA A mean intensity (x 1000 a.U.) was limited from 2 to 10, with values outside this range squished to the nearest limit. Marginal histograms represent the distribution of Hoechst and EdU intensities. Bar plot (bottom panel) shows the mean AURORA A immunofluorescence (x 1000 a.U.) signal quantified per cell cycle phase (*n* number of nuclei, µ mean, AU arbitrary units). (**B**) Quantification of spot intensity. The PLA spot intensity was analyzed and summarized per nucleus (left), cytoplasm (middle), or whole cell (right). For each condition (DICER, AURORA A, or DICER + AURORA A) three biological replicate experiments were conducted and in each case three FoVs were analyzed (total analyzed cells, DICER: 500, AURORA A: 429, DICER + AURORA A: 498). Each bar represents the mean ± SD. (**C**) Quantification of DICER/AURORA A PLA in cell cycle phases of U2OS cells. Jittered dot plot showing the number of PLA foci per nucleus for the cell cycle phases identified in U2OS cells from Fig. 4D. Overlaid boxplots show the median (center line), interquartile range (box, Q1–Q3), and whiskers extending to the most extreme values within 1.5 x interquartile range. The number of nuclei analyzed per condition is indicated as *n* ($n \geq 333$). The dotted orange line marks the threshold of 50 PLA foci per nucleus used to classify PLA-positive cells in Fig. 4D. (**D**) Quantification of DICER/AURORA A PLA in DICER knock-out cells. Jittered dot plot showing the number of PLA foci per nucleus for the combined PLA condition in DICER knock-out cells treated transiently overexpressing of wildtype (DICER wt) and mutant DICER (DICER del) and for the single-antibody controls (DICER only and AURORA A only). Overlaid boxplots show the median (center line), interquartile range (box, Q1–Q3), and whiskers extending to the most extreme values within 1.5 x interquartile range. The number of nuclei analyzed per condition (*n*) is indicated below the corresponding condition. *p* values were calculated with the Wilcoxon test. (**E**) Western blot of 5 different doxycycline-inducible shDICER MV4-11 cell lines. Cells were treated with doxycycline (1 µg/ml) or EtOH as a control for 3 days. GAPDH was used as a loading control. (**F**) Left: BrdU-PI cell cycle profile of unsynchronized MV4-11 cells stably expressing doxycycline-inducible shDICER-4 without BrdU incorporation, serving as a control for Low-BrdU S-phase cells in Fig. 4E. Right: Quantification of BrdU-PI cell cycle distribution from three independent biological replicates shown as average ± SEM. One representative replicate is displayed in Fig. 4E.

© The Author(s)

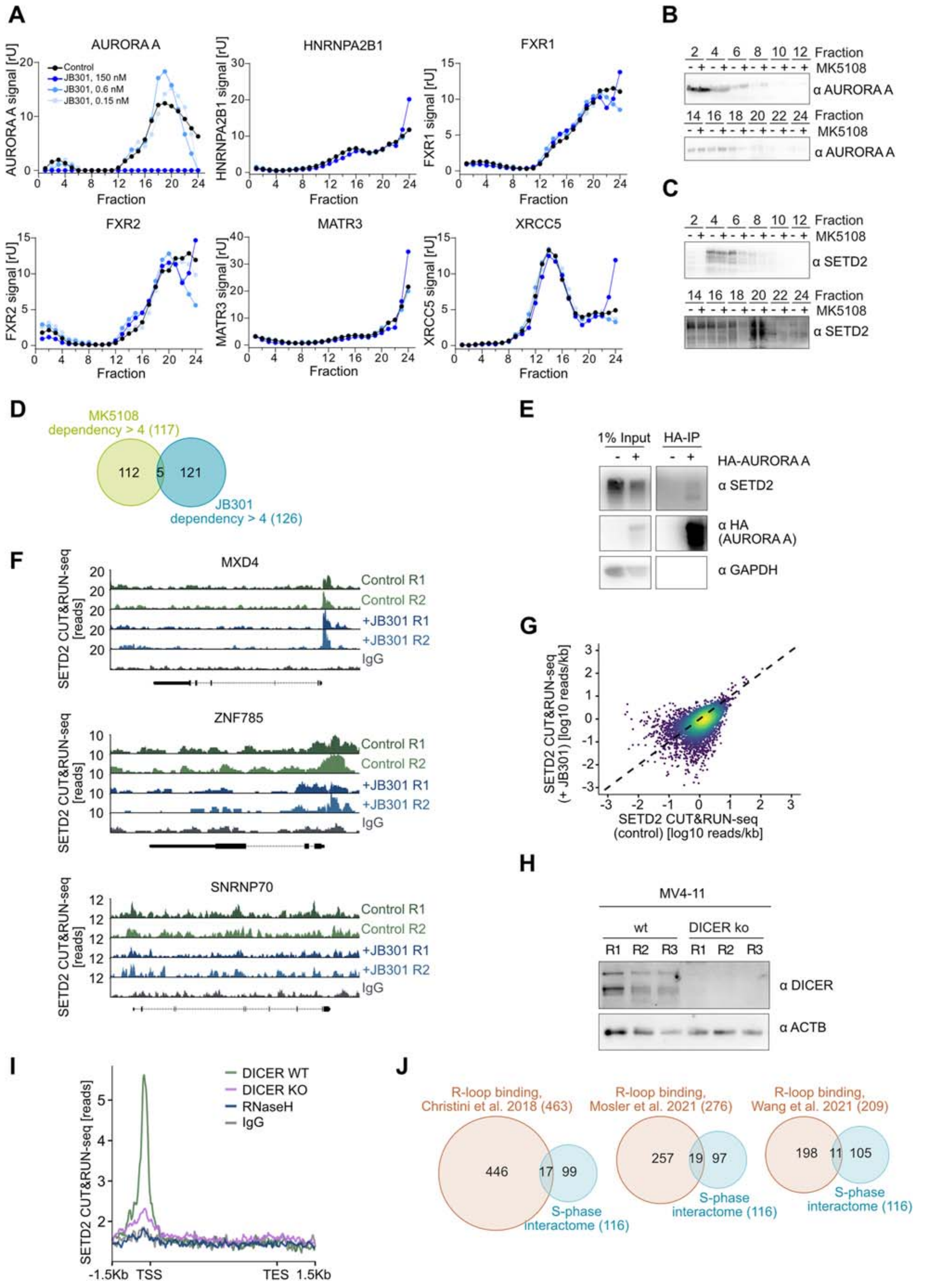
A
AURORA A
HNRNPA2B1
FXR1
FXR2
MATR3
XRCC5
Control
JB301, 150 nM
JB301, 0.6 nM
JB301, 0.15 nM
Fraction
B
C
MK5108
α AURORA A
α SETD2
D
MK5108 dependency > 4 (117)
112
5
121
JB301 dependency > 4 (126)
E
1% Input
HA-IP
HA-AURORA A
α HA (AURORA A)
α GAPDH
F
MXD4
ZNF785
SNRNP70
Control R1
Control R2
+JB301 R1
+JB301 R2
IgG
SETD2 CUT&RUN-seq [reads]
G
SETD2 CUT&RUN-seq (+ JB301) [log10 reads/kb]
SETD2 CUT&RUN-seq (control) [log10 reads/kb]
H
MV4-11
wt
DICER ko
α DICER
α ACTB
I
DICER WT
DICER KO
RNaseH
IgG
-1.5Kb TSS TES 1.5Kb
J
R-loop binding, Christini et al. 2018 (463)
446
17
99
R-loop binding, Mosler et al. 2021 (276)
257
19
97
R-loop binding, Wang et al. 2021 (209)
198
11
105
S-phase interactome (116)

Figure EV5. AURORA A supports the association of SETD2 with high-molecular-weight complexes and chromatin.

(**A**) Sedimentation profiles of RNA-dependent AURORA A interactors in a 5–50% sucrose gradient analyzed by mass spectroscopy. MV4-11 overexpressing HA-CRBN were treated ±JB301 for 6 h, lysed and loaded on a 5–50% sucrose gradient. Individual fractions were analyzed by label-free mass spectroscopy. LFQ intensities were normalized to 100 and are shown for every fraction. (**B**) Immunoblots of gradient fractions. MV4-11 cells were treated with or without an MK5108 (150 nM) for 6 h. The lysate was loaded on a 5–50% sucrose gradient, centrifuged and fractionated. The precipitated fractions were loaded on an immunoblot, and AURORA A was detected by specific antibodies. (**C**) Immunoblots of gradient fractions. MV4-11 cells were treated with or without an MK5108 (150 nM) for 6 h. The lysate was loaded on a 5–50% sucrose gradient, centrifuged and fractionated. The precipitated fractions were loaded on an immunoblot, and SETD2 was detected by specific antibodies. Please note that the lower membrane was exposed for a longer duration to better visualize the differential gradient migration behavior of SETD2 induced by MK5108. (**D**) Global analysis of AURORA A PROTAC and inhibitor dependency. MV4-11 HA-CRBN cells were treated with either JB301 (150 and 0.6 nM) or with MK5108 (150 nM) for 6 h. The lysate was loaded on a 5–50% sucrose gradient, centrifuged, fractionated, and the proteins were analyzed by label-free mass spectroscopy. The PROTAC/ inhibitor-dependency was calculated by the division of heavy (F12–F24) and light fractions (F1–F11) for DMSO and PROTAC or inhibitor conditions, respectively. The quotient of DMSO and PROTAC/inhibitor score was defined as PROTAC-/inhibitor-dependency, respectively. For the Venn diagram, only proteins which were detected in both conditions were taken into consideration. (**E**) Immunoblot of exogenous HA-AURORA A IP showing binding of AURORA A to SETD2 WT. HEK293 cells were transfected with HA-AURORA A WT, and co-IP was performed using magnetic beads. (**F**) Genome browser track of the SETD2 CUT&RUN signal in MV4-11 cells treated with DMSO or 150 nM JB301 for 6 h. The binding of the MXD4, SNRNP70, and ZNF785 genes are shown and compared to IgG as a control. (**G**) Scatter plot of IgG control-subtracted SETD2 CUT&RUN-seq signals in the gene body (TSS + 1 kb until TES) of MV4-11 cells treated with DMSO or 150 nM JB301 for 6 h. (**H**) Immunoblot of DICER levels in wt and Dicer KO MV4-11 cells. ß-Actin was used as a loading control (ACTB). (**I**) Metagene plots of mean SETD2 CUT&RUN-seq signals of DICER wildtype, DICER knock-out MV4-11 cells and DICER wildtype MV4-11 cells treated with RNase H after cell permeabilization in expressed genes ($n = 10,821$) compared to IgG as a negative control. (**J**) Identification of R-loop binding AURORA A interactors. Various R-loop databases were screened for AURORA A interactors. The databases are from three different publications (Cristini et al, 2018; Mosler et al, 2021; Wang et al, 2018), each of which employs a distinct approach to identifying R-loop-binding proteins.

 © The Author(s)
