# Supplementary material for: AURORA A interacts with DICER and SETD2 to promote S-phase progression
Source: EMBO Rep. 2026 Jun 30;27(15):4493–523. doi: 10.1038/s44319-026-00850-0 (PMC13458143; doi:10.1038/s44319-026-00850-0)
Supplement: Supplementary file 2 [file 44319_2026_850_MOESM1_ESM.pdf]

EMBO reports

AURORA A interacts with DICER and SETD2 to promote S-phase progression

Juliane Müller, Tina Daunke, Nicola Berner, Pranjali Bhandare, Anneli Gebhardt, Lasse Hoffmann, Mathias Diebold, Markus Vogt, Julia Hofstetter, Yiliam Cruz-Garcia, Nevenka Dudvarski-Stankovic, Katharina Schneider, Bikash Adhikari, Stephanie Lamer, Andreas Schlosser, Gabriele Büchel, Martin Sos, Bernhard Küster, Stefan Knapp, Stephanie Wilhelm, and Elmar Wolf

Corresponding author(s): Elmar Wolf (elmar.wolf@biochem.uni-kiel.de) , Stephanie Wilhelm (stephanie.wilhelm@tum.de)

Review Timeline:

Submission Date:	29th Oct 25
Editorial Decision:	17th Dec 25
Revision Received:	30th Apr 26
Editorial Decision:	2nd Jun 26
Revision Received:	9th Jun 26
Accepted:	16th Jun 26

Editor: Achim Breiling

Transaction Report:

(Note: With the exception of the correction of typographical or spelling errors that could be a source of ambiguity, letters and reports are not edited. Depending on transfer agreements, referee reports obtained elsewhere may or may not be included in this compilation. Referee reports are anonymous unless the Referee chooses to sign their reports.)

1st Editorial Decision **17th Dec 2025**

Dear Prof. Wolf,

Thank you for the submission of your manuscript to EMBO reports. I have now received reports from the three referees that were asked to evaluate your study, which can be found at the end of this email. As you will see, the referees think that these findings are of high interest. However, they have several comments, concerns, and suggestions, indicating that a major revision of the manuscript is necessary to allow publication of the study in EMBO reports. As the reports are below, I will not detail them here.

Given the constructive referee comments, I would thus like to invite you to revise your manuscript with the understanding that the concerns of the referees must be addressed in the revised manuscript and/or in a detailed point-by-point response. Acceptance of your manuscript will depend on a positive outcome of a second round of review. It is EMBO reports policy to allow a single round of revision only and acceptance of the manuscript will therefore depend on the completeness of your responses included in the next, final version of the manuscript.

Revised manuscripts should be submitted within three months of a request for revision. Please contact me to discuss the revision (also by video chat) if you have questions or comments regarding the revision, or should you need additional time.

When submitting your revised manuscript, please also carefully review the instructions that follow below.

PLEASE NOTE THAT upon resubmission revised manuscripts are subjected to an initial quality control prior to exposition to re-review. Upon failure in the initial quality control, the manuscripts are sent back to the authors, which may lead to delays. Frequent reasons for such a failure are the lack of the data availability section (please see below) and the presence of statistics based on n=2 (the authors are then asked to present scatter plots or provide more data points).

When submitting your revised manuscript, we will require:

1) a .docx formatted version of the final manuscript text (including legends for main figures, EV figures and tables), but without the figures included. Figure legends should be compiled at the end of the manuscript text.

2) individual production quality figure files as .eps, .tif, .jpg (one file per figure), of main figures and EV figures. Please upload these as separate, individual files upon re-submission.

The Expanded View format, which will be displayed in the main HTML of the paper in a collapsible format, has replaced the Supplementary information. You can submit up to 6 images as Expanded View. Please follow the nomenclature Figure EV1, Figure EV2 etc. The figure legend for these should be included in the main manuscript document file in a section called Expanded View Figure Legends after the main Figure Legends section. Additional Supplementary material should be supplied as a single pdf file labeled Appendix. The Appendix should have page numbers and needs to include a table of content on the first page (with page numbers) and legends for all content. Please follow the nomenclature Appendix Figure Sx, Appendix Table Sx etc. throughout the text, and also label the figures and tables according to this nomenclature.

For more details, please refer to our guide to authors:
https://link.springer.com/journal/44319/submission-guidelines#cms-Revised-submissions

Please consult our guide for figure preparation:
https://media.springernature.com/original/springer-cms/rest/v1/content/27825798/data/v1

3) a .docx formatted letter INCLUDING the reviewers' reports and your detailed point-by-point responses to their comments. As part of the EMBO Press transparent editorial process, the point-by-point response is part of the Review Process File (RPF), which will be published alongside your paper.

4) a complete author checklist, which you can download from our author guidelines (https://link.springer.com/journal/44319/submission-guidelines#cms-Revised-submissions). Please insert page numbers in the checklist to indicate where the requested information can be found in the manuscript. The completed author checklist will also be part of the RPF.

Please also follow our guidelines for the use of living organisms, and the respective reporting guidelines:
https://link.springer.com/partners/embo-press/editorial-policies#Animal%20and%20human%20studies

5) that primary datasets produced in this study (e.g. RNA-seq, ChIP-seq, structural and array data) are deposited in an appropriate public database. If no primary datasets have been deposited, please also state this in a dedicated section (e.g. 'No primary datasets have been generated and deposited'), see below.

See also: https://link.springer.com/partners/embo-press/editorial-policies#Data%20deposition

Please remember to provide a reviewer password if the datasets are not yet public.

The accession numbers and database should be listed in a formal "Data Availability" section that follows the model below. This is now mandatory (like the COI statement). Please note that the Data Availability Section is restricted to new primary data that are part of this study. This section is mandatory. As indicated above, if no primary datasets have been deposited, please state this in this section

Data availability

The datasets produced in this study are available in the following databases:

- RNA-Seq data: Gene Expression Omnibus GSE46843 (https://www.ncbi.nlm.nih.gov/geo/query/acc.cgi?acc=GSE46843)
- [data type]: [name of the resource] [accession number/identifier/doi] ([URL or identifiers.org/DATABASE:ACCESSION])

*** Note - All links should resolve to a page where the data can be accessed. ***

See also: https://link.springer.com/partners/embo-press/editorial-policies#Data%20availability%20statement

Moreover, I have these editorial requests:

6) We now request the publication of original source data with the aim of making primary data more accessible and transparent to the reader. You will receive a separate email with instructions for providing source data with your revised manuscript, including information how to upload and organize the files.

7) Our journal encourages inclusion of *data citations in the reference list* to directly cite datasets that were re-used and obtained from public databases. Data citations in the article text are distinct from normal bibliographical citations and should directly link to the database records from which the data can be accessed. In the main text, data citations are formatted as follows: "Data ref: Smith et al, 2001" or "Data ref: NCBI Sequence Read Archive PRJNA342805, 2017". In the Reference list, data citations must be labeled with "[DATASET]". A data reference must provide the database name, accession number/identifiers and a resolvable link to the landing page from which the data can be accessed at the end of the reference. Further instructions are available at: https://link.springer.com/journal/44319/submission-guidelines#cms-Reference-guidelines

8) Regarding data quantification and statistics, please make sure that the number "n" for how many independent experiments were performed, their nature (biological versus technical replicates), the bars and error bars (e.g. SEM, SD) and the test used to calculate p-values is indicated in the respective figure legends (also for EV and Appendix figures). Please also check that all the p-values are explained in the legend, and that these fit to those shown in the figure. Please provide statistical testing where applicable. Please avoid the phrase 'independent experiment' but clearly state if these were biological or technical replicates. Please also indicate (e.g. with n.s.) if testing was performed, but the differences are not significant. In case n=2, please show the data as separate datapoints without error bars and statistics. See also:
https://link.springer.com/journal/44319/submission-guidelines#cms-Figure-and-data-presentation

If n<5, please show single datapoints for diagrams.

9) Please add scale bars of similar style and thickness to all microscopic images, using clearly visible black or white bars (depending on the background). Please place these in the lower right corner of the images themselves. Please do not write on or near the bars in the image but define the size in the respective figure legend.

10) Please also note our reference format:
https://link.springer.com/journal/44319/submission-guidelines#cms-Reference-guidelines

11) We updated our journal's competing interests policy in January 2022 and request authors to consider both actual and perceived competing interests. Please review the policy https://www.embopress.org/competing-interests and update your competing interests if necessary. Please name this section 'Disclosure and Competing Interests Statement' and put it after the Acknowledgements section.
See: https://link.springer.com/partners/embo-press/editorial-policies#Competing%20interest%20disclosures

12) We now use CRediT to specify the contributions of each author in the journal submission system. CRediT replaces the author contribution section. Please use the free text box to provide more detailed descriptions and do NOT provide your final manuscript text file with an author contributions section. See also our guide to authors (section 'Author contributions'):
https://link.springer.com/journal/44319/submission-guidelines#cms-Revised-submissions

13) All Materials and Methods need to be described in the main text using our 'Structured Methods' format, which is required for

all research articles. According to this format, the Methods section should include a Reagents and Tools Table (listing key reagents, experimental models, software, and relevant equipment and including their sources and relevant identifiers), uploaded as separate file, and a Methods section in which we encourage the authors to describe their methods using a step-by-step protocol format with bullet points, to facilitate the adoption of the methodologies across labs. More information on how to adhere to this format as well as downloadable templates (.doc) for the Reagents and Tools Table can be found in our author guidelines (section 'Structured Methods'):

https://link.springer.com/journal/44319/submission-guidelines#cms-Manuscript-organisation-and-formatting

14) Please order the manuscript sections like this, using only these names:
Title page - Abstract (max. 175 words) - Keywords - Introduction - Results - Discussion - Methods - Data availability section - Acknowledgements (please include here all the funding information) - Disclosure and Competing Interests Statement - References - Figure legends - Expanded View Figure legends

15) Please make sure that all the funding information is also entered into the online submission system and that it is complete and similar to the one in the acknowledgement section of the manuscript text file.

We would also welcome the submission of cover suggestions, or motifs to be used by our Graphics Illustrator in designing a cover.

As part of the EMBO publication's Transparent Editorial Process, EMBO reports publishes online a Review Process File (RPF) to accompany accepted manuscripts. This File will be published in conjunction with your paper and will include the referee reports, your point-by-point response and all pertinent correspondence relating to the manuscript.

You are able to opt out of this by letting the editorial office know (emboreports@embo.org). If you do opt out, the Review Process File link will point to the following statement: "No Review Process File is available with this article, as the authors have chosen not to make the review process public in this case."

I look forward to seeing a revised form of your manuscript when it is ready.

Yours sincerely,

Achim Breiling
Senior Editor
EMBO Reports

Referee #1:

The paper by Müller et al. investigates the non-catalytic functions of Aurora A kinase during the S-phase of the cell cycle. Previous studies have highlighted the importance of AurA's scaffolding roles beyond its kinase activity, particularly in the context of PROTAC-mediated degradation, which induces S-phase arrest in cells lacking N-MYC. This study extends those findings by profiling the AurA S-phase interactome in AML cells deficient in N-MYC, revealing a significant association with RNA-binding proteins, including DICER and SETD2-both of which had not previously been characterized as AurA substrates. The data presented support a model in which AurA, DICER, and SETD2 collaboratively manage replicative stress, thereby enhancing our understanding of the molecular mechanisms underlying S-phase regulation in cancer cells.

The paper is clearly written, and the data are robust. However, before publication, the authors should address the following issues:

Figure 1B: The AurA-HiBIT construct/method is not described anywhere in the Materials and Methods section or in the Results or figure legend.

Figure 1D: The figure legend mentions the use of the inhibitor MK5109, but no corresponding data are shown in the panel.

Figure 1I: There is an inconsistency regarding the sucrose gradient: the figure legend refers to a 10-50% gradient, whereas the Materials and Methods, figure legends, and Results sections describe a 5-50% gradient. Which is correct? Additionally, please clarify how the gradient fractionation was performed-was it collected from top to bottom or from bottom to top? Based on the numbering of the fractions, it appears to have been done from top to bottom; is this correct?

Figures 1D and 4D: There is a discrepancy between the percentage of S-phase cells shown in these two figure panels. The proportion of S-phase cells in all conditions in Figure 4D is much higher than in Figure 1D. It seems likely that the cells in Figure

4D were synchronized by double thymidine block (as described in the Materials and Methods) but this is not mentioned in the Results section or in the Figure 4 legend, whereas the cells in Figure 1D may not have been synchronized. Is this the case? If not, please explain the difference.

Figure 2E: Why are HNRNPA2B1, FXR2, and XRCC5 not shown in the bar graph?

Figure 3G and EV3C: The nomenclature of the AurA mutants differs between Figure 3G, Figure EV3C, and the Results section, which creates confusion. Please standardize the naming across all sections.

Page 9, last paragraph: The authors state "partial colocalization of endogenous Aurora A and DICER." This seems overstated, as the colocalization appears only within the general nuclear region, with AurA showing diffuse staining and DICER appearing in small speckles. Based on this staining alone, one cannot conclude colocalization. However, the presented PLA data indeed support such colocalization.

Given the data suggesting that DICER, together with AurA and SETD2, may localize to R-loops, it would be worthwhile to test whether the observed DICER nuclear speckles indeed colocalizes with R-loops using the R-loop-specific S9.6 antibody. Such an experiment would strengthen the authors' conclusions.

Please edit in page 8 end of second paragraph 'leucin' to 'leucine'.

Referee #2:

The manuscript by Muller and colleagues describes an interesting study combining MS interactomics and proteomics with bioinformatic modelling and cell biology, to investigate the scaffolding roles of Aurora-A in S-phase. By generating the S-phase interactome of AurkA authors identify two partners, Dicer and SETD2, which may be highly relevant in mediating these roles. The experimental plan is well conceived and executed, results are clearly exposed, and conclusions are relevant and timely in the field.

MS experiments provide a valuable resource for the analysis of AurkA S-phase interactors and their dependency on RNA presence. The methodological approach, the use of AlphaFold-based structural modeling and the results presented in the modeling section are rigorous and compelling. The strategy for dissecting DICER, given its considerable size, into four well-defined regions based on domain architecture is logically sound. The structural analogy to the TPX2 interaction, including conservation of key hydrophobic and aromatic contact points, adds further plausibility to the model. The biochemical validation is well performed. The observed loss of interaction upon mutation of the Y-pocket or the combined Y/F/W pockets strongly corroborates the AlphaFold predictions and demonstrates functional relevance of the identified interface.

Overall, the integration of structural modeling with experimental validation is a strength of this study. The data support the conclusion that DICER interacts directly with AurkA via a TPX2-like mode of binding centered on the Y-pocket, providing an insightful mechanistic basis for the interaction. On the other hand, the validation and investigation of the functional relevance of proposed interactions in cell biology experiments requires additional experiments to support the drawn conclusions. I list below major and minor points to be addressed before publication.

Major points:

- I am not sure about the FACS analyses (Figs 1D, 4D). If I understand correctly, data are from a pulse BrdU (1 hour) before fixation, so that only cells passing through S-phase during the 1- hour interval should be positive. All cells above the BrdU threshold should be quantified as incorporating ones, while it seems to me that only the "green" window has been quantified. The "red" region would represent cells with an S-phase DNA content by PI, but not incorporating and thus not in active synthesis. This is interesting and it is evident from the profiles that there are changes occurring upon treatments. A complete analysis of all cell cycle windows (including G1 and G2/M phases) with quantifications and SDs is required to better depict cell cycle progression upon the different investigated conditions. Related to this, the title of the manuscript "S-phase AURORA A interacts with DICER and SETD2 to maintain cell cycle progression" is in my opinion overstated with respect to results as described in the actual form.

- Interaction between AurkA and Dicer by PLA is interesting. Are positive cells in S-phase? Is the interaction increased upon replication stress? Is it impaired by using the mutants described in Figure 3, thus supporting the identified regions of interaction? Can these mutants be used, in parallel to AurkA PROTACs, in functional assays to show the relevance of the Aurora-A/Dicer interaction in resolving R-loops (e.g. R-loop detection using specific antibodies to see their increase)?

- The SETD2/AurkA interaction and the possibility of a ternary complex SETD2/AurkA/Dicer are interesting but have not been pursued. Therefore, additional experimental evidence is required in my opinion to validate some of the assumptions of the proposed model: can authors demonstrate an AurkA/SETD2 interaction in cells (e.g. by PLA)? And can they demonstrate

Dicer/SETD2 interaction? If so, then the model would predict that AurkA PROTAC, and/or AurkA and Dicer mutants described before, would impair the interaction, which should be quite feasible to demonstrate. And, if the complex mediates SETD2 recruitment, do authors observe defective recruitment by silencing DICER, since they have the experimental setting in place?
- Although the scaffolding role is intriguing, using AurkA inhibitors in some of the key experiments of the manuscript would strengthen the conclusion that kinase activity is not required. It is also possible that both scaffolding and phosphorylation-based effects exist, which is an interesting possibility. Authors also mention this case for SETD2, for which a phosphorylation by AurkA has been described with destabilizing effect. Since authors build a possible scenario in the Discussion for this dual effect, it would be interesting to insert this as a possible mechanism in the Model (Figure 6).

Minor points:

- p. 5. If from 117 interactors 10 non-substrates were removed, why the remaining ones are 106? Maybe AurkA itself makes the difference, but I find the text as it is slightly confusing.
- Legend 1G reads "as in 1G", I guess it should be "as in 1F".
- From data shown in Fig. 1H TPX2 appears as a non RNA-mediated interaction, which is different from what was observed by Rajagopal et al., 2025. It would be interesting to comment on this cell cycle difference in the Discussion.
- p. 6. "These findings indicated that AURORA A assembles into extensive protein- protein complexes with RNA-binding proteins during S-phase, many of which have not been previously identified as substrates of AURORA A in phosphoproteomic analyses". I feel this is overstated before showing results coming in the following figures. At this stage I do not find that interacting with RBPs and existing in large complexes necessarily means that the large complexes include RBPs.
- Figure 4. AurkA is cell cycle regulated. Why are all cells in the IF panels positive and nuclear? This would be expected only in a G2-enriched culture. Is this the case?
- Figure 4C: add number of analysed cells per condition.
- "Of all proteins identified in the S-phase interactome, 12 showed a significant shift toward lighter fractions upon RNase treatment (Fig. 2E). Among these were 8 RNA- binding proteins, including DICER, HNRNPA2B1, FXR1, FXR2 YLPM1 and XRCC5 (Fig. 2F)." It is not clear to me the correspondence between this sentence and Figure panels: if I look at the 12 significant factors in Figure 2E, RBPs are more than 8. On the other hand, not all proteins listed in the second sentence are included in Figure 2E. Can authors better explain this point?
- p.11. We next investigated whether distinct gene subsets differing in expression strength, length, transcription factor binding sites or chromatin status were particularly affected by SETD2 loss. Strikingly, this analysis revealed a strong association between R-loops and SETD2 chromatin binding." Are the analyses indicated in the first sentence "not shown"?

Referee #3:

Müller et al. seek to characterize the scaffolding properties of AURORA A kinase in S-phase, a key aspect of its oncogenic function independent of its catalytic activity. One key gap in AURORA A's biological role is its operation independent of N-MYC. Interestingly, the degradation of AURORA A results in S-phase arrest, but catalytic inhibition results in G2/M arrest. The authors therefore assay AURORA A's binding partners in a human acute myeloid leukemia (AML) cell line, where N-MYC is not expressed. Through extensive proteomics analysis and biochemical characterization, the authors find two populations of AURORA A complexes and investigate several interactors. Interestingly, only a subset of these complexes depends on RNA, despite a prevalence of RNA binding proteins. The authors then go on to demonstrate that these binders likely result from protein-protein interactions, poising AURORA A as a key scaffold in addition to its catalytic activity. Key proteins include DICER and SETD2, suggesting a novel role in alleviating transcription-replication conflict.

The manuscript substantiates the importance of these interactions by finding a redistribution of SETD2 under AURORA A depletion, by identifying a direct binding site between AURORA A and DICER, and by finding significant correspondence to R-loops. Considering these results together, this work stands to significantly advance the field by extensive characterization of AURORA A outside of its catalytic activity, with potentially far-reaching consequences for pathologies. Overall, the manuscript is well-prepared and offers appropriate interpretations of the presented data. Below, we suggest minor revisions.

- Because the proposed mechanism appears R-loop associated, a deeper analysis of the existing dataset with RNase H treatment may be warranted.
- Does degrading AURORA A with PROTAC cause its interacting partners to be destabilized? This could be addressed by comparing mass spectrometry datasets in AURORA A positive versus depleted cells.
- The sucrose gradient fractionation in Figure 1J and Figure 2B should be accompanied by the UV trace of these experiments to demonstrate the statement on p6 "corresponding to approximately 80S" - perhaps as a separate plot in the supplement. Additionally, Figure 1J's plot is stretched horizontally, distorting the points and error bars.
- p6 lines 3-5: Expressing C-terminally and N-terminally HA-tagged AURORA A may not constitute four biological replicates if a population of AURORA A is proteolytically cleaved before analysis, or if the tags interfere with different interactors. Thus, analyzing both tags makes the conclusions more robust, but an additional sentence or a change in wording may be warranted.
- The authors should consider citing [PMID:41161313] and [PMID:35194019] which indicate a role for dicer in R-loop management and AURORA B function, respectively.

- It is interesting that fractions 8-12 lack AURORA A complexes, forming two regimes that agree with the authors' later "light" versus "heavy" analyses. Can the authors speculate why the variations in complexes skip the middle fractions, such as association with a specific larger element?

While this manuscript is well-prepared, there are a few minor grammatical inconsistencies:

- p3 line 12: "well-characterized" is hyphenated elsewhere, but not here.
- p4 lines 4-5: this clause should use two em dashes-for example, like this-rather than hyphens (also: p10 line 8, third to last line of p12, p13 line 14, and p14 line 10).
- p7 second to last line: "recruit" rather than "recruited".
- p10 third to last line: "high-molecular-weight" is not hyphenated previously.
- p10 last line and later on p11: CUT&RUN is an acronym and should be capitalized.
- p30 under (G), "RNAse" should be "RNase".
- p31 under (J) "the highest signal was set to "1" should reflect the y-axis significant figures with "1.0".
- p31 under (C) (and later for (Figure 3A)) high-molecular-weight should be hyphenated for consistency.
- p31 under (Figure 3A) "overlayed" should be "overlaid".
- p32 under (E), missing a space between "AURORAA".
- p33 under (C) "3 independent experiments" - "three" is spelled out in previous captions when referring to replicates (again on p36 under Figure EV4 A).
- p35 under (Figure EV1 C) "Triazole" should be "trizol" or (branded) "TRIzol".

Response to the reviewers

First of all, we would like to thank the reviewers for their very constructive comments, which helped us to improve the manuscript! In response to the raised issues, we have performed new experiments and analyses and made the following changes:

Reviewer #1

> ***General comments**: The data presented support a model in which AurA, DICER, and SETD2 collaboratively manage replicative stress, thereby enhancing our understanding of the molecular mechanisms underlying S-phase regulation in cancer cells. The paper is clearly written, and the data are robust.*

Response: We thank the reviewer for the positive evaluation of our manuscript and for the exceptionally careful and constructive comments.

> ***#1-1:** Figure 1B: The AurA-HiBIT construct/method is not described anywhere in the Materials and Methods section or in the Results or figure legend.*

Response: We apologize for the insufficient description of the HiBiT assay. As in our previous studies, we used lentivirally transduced MV4-11 cells stably expressing HiBiT-tagged AURORA A under the control of a weak promoter (PGK). The assays quantify luciferase activity following 6 hours of PROTAC treatment and subsequent cell lysis, compared to untreated control cells. We have now included a description of the HiBiT system in the Methods section of the revised manuscript and have listed the primers used in the revised **Dataset EV5**.

> ***#1- 2:** Figure 1D: The figure legend mentions the use of the inhibitor MK5109, but no corresponding data are shown in the panel.*

Response: We corrected the figure legend.

> ***#1-3:** Figure 1I: There is an inconsistency regarding the sucrose gradient: the figure legend refers to a 10-50% gradient, whereas the Materials and Methods, figure legends, and Results sections describe a 5-50% gradient. Which is correct? Additionally, please clarify how the gradient fractionation was performed-was it collected from top to bottom or from bottom to top? Based on the numbering of the fractions, it appears to have been done from top to bottom; is this correct?*

Response: Thank you for pointing out this inconsistency. We used a 5–50% gradient and have corrected the corresponding information in the Figures. new **Figure 1I**. We have also added that the gradient was collected from top to bottom in the methods section.

> ***#1-4:** Figures 1D and 4D: There is a discrepancy between the percentage of S-phase cells shown in these two figure panels. The proportion of S-phase cells in all conditions in Figure 4D is much higher than in Figure 1D. It seems likely that the cells in Figure 4D were synchronized by double thymidine block (as described in the Materials and Methods) but this is not mentioned in the Results section or in the Figure 4 legend, whereas the cells in Figure 1D may not have been synchronized. Is this the case? If not, please explain the difference.*

Response: The cells analyzed for cell-cycle distribution in Figures 1D and 4D were not synchronized but derived from asynchronous cultures. Indeed, the proportion of cells in S phase differed between the two experiments in the original manuscript for three reasons. First, the experiment shown in Figure 4D contained a somewhat higher fraction of cells in S phase than that shown in Figure 1D. Although we strive to maintain highly comparable culture

conditions, we routinely observe minor variations in cell-cycle distribution at the time of analysis. Furthermore, the cells in Figure 4D were stably transduced with a doxycycline-inducible shDICER construct, whereas Figure 1D analyzed naïve cells, which may lead to slight differences in cell cycle distribution under unperturbed conditions. Second, labeling efficiency in the experiment presented in Figure 4D was slightly higher than in Figure 1D, as reflected by a stronger separation between BrdU-negative and BrdU-positive populations. Third, slightly different gating strategies were initially applied.

We have now harmonized the gating procedure across experiments, resulting in a more comparable fraction of S-phase cells under control conditions. Importantly, the effects of AURORA A inhibition and degradation on cell-cycle distribution were consistent across both experiments (and all additional replicates): catalytic inhibition led to pronounced G2/M accumulation and a reduction in S-phase cells, whereas PROTAC-induced degradation of AURORA A resulted in an increased fraction of S-phase cells that failed to incorporate BrdU.

In the revised manuscript, **Figures 1D** and the new **Figure 4E (originally 4D)** display the results obtained using the unified gating strategy. Additional analysis and the respective gating schemes are provided in **Figure EV1B, C** and **EV4F**, and the figure legends now specify that the analyses were performed in asynchronous cell populations.

> ***#1-5:*** *Figure 2E: Why are HNRNPA2B1, FXR2, and XRCC5 not shown in the bar graph?*

Response: We thank the reviewer for pointing out this inconsistency. In Figure 2E, we showed only the AURORA A interactors identified in the first proteomics experiment, in which AURORA A was immunoprecipitated from S-phase–synchronized cells without additional treatment. HNRNPA2B, FXR2, and XRCC5 were not among the 116 AURORA A interactors when applying stringent cut-off criteria in this experiment, but they were detected as interactors in the experiment directly comparing the effects of RNase treatment on AURORA A interactions. Although the proteomics results across all experiments are highly consistent, minor statistical differences can result in the inclusion or exclusion of a few proteins between independent experiments.

To avoid confusion for the reader, we have replaced the three candidates with SMNDC1, EXOSC2 and ELAVL1 in **Figure 2F** in the revised version, and we continue to focus on the initially described set of 116 interactors in the Results section. Furthermore, we have now made the data from the independent proteomics experiments accessible by including supplementary tables for each mass spectrometry experiment (**Dataset EV1, EV2, EV3**), in addition to uploading the raw data to the PRIDE repository (PXD076528: reviewer_pxd076528@ebi.ac.uk, password: tfnVpLbO2yqX and PXD069843: reviewer_pxd069843@ebi.ac.uk, password: ObDsbg25Pr0h).

> ***#1-6:*** *Figure 3G and EV3C: The nomenclature of the AurA mutants differs between Figure 3G, Figure EV3C, and the Results section, which creates confusion. Please standardize the naming across all sections.*

Response: We have harmonized the nomenclature of the AURORA A mutants throughout the manuscript ("HA–AURORA A mut1" and "HA–AURORA A mut2").

> ***#1-7:*** *Page 9, last paragraph: The authors state "partial colocalization of endogenous Aurora A and DICER." This seems overstated, as the colocalization appears only within the general nuclear region, with AurA showing diffuse staining and DICER appearing in small speckles. Based on this staining alone, one cannot conclude colocalization. However, the presented PLA data indeed support such colocalization.*

Response: We agree and changed the statement from "partial colocalization" to "partial nuclear localization".

> ***#1-8:** Given the data suggesting that DICER, together with AurA and SETD2, may localize to R-loops, it would be worthwhile to test whether the observed DICER nuclear speckles indeed colocalizes with R-loops using the R-loop-specific S9.6 antibody. Such an experiment would strengthen the authors' conclusions.*

Response: We agree with the reviewer and have performed the suggested experiment. First, we encountered the technical limitation that both the DICER antibody used in this study and the R-loop–detecting antibody S9.6 are of murine origin. To overcome this, we employed a modified version of the S9.6 antibody that can be detected with an anti-rabbit secondary antibody (Ab01137-23.0, Absolute Antibody). In co-immunofluorescence experiments, we indeed observed that cells displaying nuclear DICER speckles also showed co-localization with the S9.6 signal (**Reviewer Figure R1A**). However, it should be noted that the rabbit S9.6 antibody also prominently stains cytoplasmic structures, both after methanol and PFA fixation (**Reviewer Figure R1B**). Therefore, we refrained from performing further quantitative analyses and kindly ask the reviewer to accept a qualitative description of S9.6-positive DICER speckles. While we have not included these images in the manuscript due to these technical limitations, we would be happy to incorporate them into the manuscript if the reviewer considers it essential.

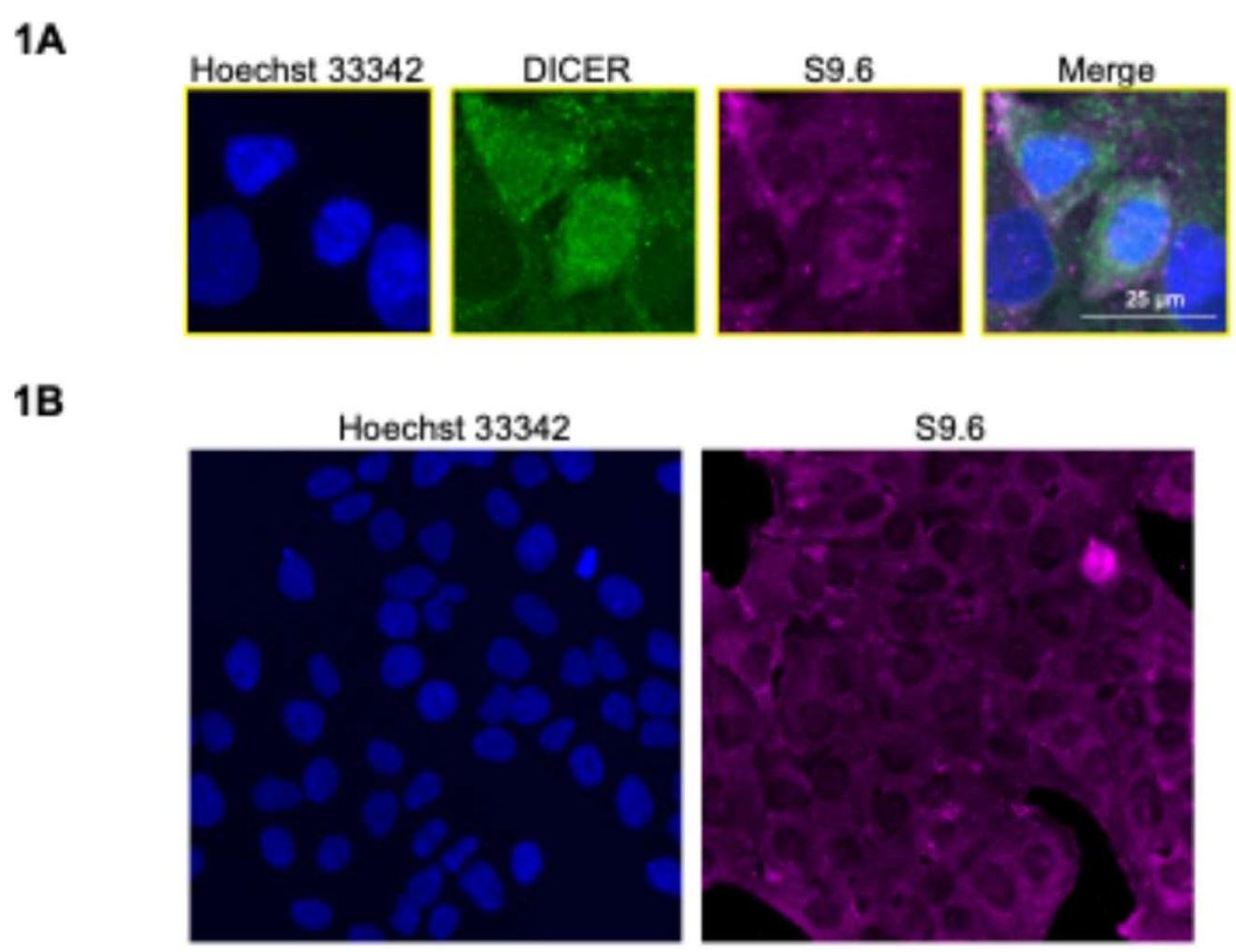

Figure R1. Immunofluorescence of DICER and R-loops in U2OS cells. (**A**) Partial co-localization of R-loops (S9.6, magenta, λ647) and DICER (green, λ488) in nuclear speckles of HOECHST-stained (blue, λ405) cells. Images were acquired with 20x objective and a 2.5x magnified cropped section is shown. Scale bar 25 µm. (**B**) Overview of immunofluorescence with the rabbit-S9.6 antibody in HOECHST stained cells.

We would like to emphasize that we have performed additional experiments and analyses that further support a functional connection between the AURORA A/DICER/SETD2 axis and R-loops, in response to this question and a question raised by Reviewer 3 (**#3-1**). We provide our response here for your convenience:

"We agree with the reviewer that the role of R-loops in the function of the S-phase AURORA A complex is an interesting question and warrants further investigation, e.g. by using R-loop–specific RNase H. Accordingly, we have performed additional analyses as well as an additional experiment. First, we analyzed published datasets for R-loop–interacting proteins, including those identified by Mosler et al. using the R-loop–binding domain of RNase H (Mosler *et al*, 2021), by Cristini et al. using the S9.6 R-loop antibody (Cristini *et al*, 2018), and by Wang et al.

using a synthetic hybrid R-loop–binding protein (Wang *et al*, 2018). Interestingly, many AURORA A–binding proteins were present in these datasets. These results have been incorporated into the revised manuscript as Venn diagrams (**Figure EV5I**) and a table (**Dataset EV4**).

Second, we investigated whether SETD2 binding to specific chromatin regions is dependent on R-loops. As already shown in the original version of the manuscript by CUT&RUN experiments, PROTAC-mediated degradation of AURORA A reduced SETD2 binding to genic regions. We have now repeated the CUT&RUN experiment in cells pretreated with recombinant RNase H. Remarkably, we observed a complete loss of SETD2 binding to chromatin, as demonstrated across a large genomic region (new **Figure 5K**) and by metagene analysis (new **Figure EV5I**) of highly SETD2-bound genes. We thank the reviewer for suggesting this experiment and believe that this strong global correlation between DICER, R-loops and SETD2 is intriguing and may open up new avenues for future studies."

#1-9: *Please edit in page 8 end of second paragraph 'leucin' to 'leucine'.*

Response: We have corrected this error. We would like to thank Reviewer 1 again for the careful reading and the very constructive comments.

Reviewer #2

> ***General comments**: The manuscript by Muller and colleagues describes an interesting study … The experimental plan is well conceived and executed, results are clearly exposed, and conclusions are relevant and timely in the field. … The methodological approach, the use of AlphaFold-based structural modeling and the results presented in the modeling section are rigorous and compelling. … The biochemical validation is well performed. … On the other hand, the validation and investigation of the functional relevance of proposed interactions in cell biology experiments requires additional experiments to support the drawn conclusions.*

Response: We thank the reviewer for their positive and very careful evaluation. We agree that the manuscript could be further strengthened by the inclusion of additional functional data. Unfortunately, since submission of the original version, the PhD student who performed the majority of the work (Juliane Müller) has left the lab. In light of this, I discussed with the editorial team at EMBO, who agreed that the current level of functional insight is in principle sufficient for EMBO Reports but not for The EMBO Journal (Editor: "*That said, given the importance of the subject, the relevant approaches, and the variety of interesting observations, we feel that the study may nevertheless be suited for our sister journal EMBO Reports, which aims at publishing relevant key results even without full mechanistic understanding.*").

However, we appreciate the reviewer's constructive suggestions and have followed them wherever possible for us. As a result, we were able to gain additional functional insights during the revision process. We have incorporated these new data (and further authors) into the revised manuscript and have toned down our conclusions where they were previously overstated. We hope that, in its revised form, the manuscript will now meet the reviewer's expectations.

> ***#2-1:** I am not sure about the FACS analyses (Figs 1D, 4D). If I understand correctly, data are from a pulse BrdU (1 hour) before fixation, so that only cells passing through S-phase during the 1- hour interval should be positive. All cells above the BrdU threshold should be quantified as incorporating ones, while it seems to me that only the "green" window has been quantified. The "red" region would represent cells with an S-phase DNA content by PI, but not incorporating and thus not in active synthesis. This is interesting and it is evident from the profiles that there are changes occurring upon treatments. A complete analysis of all cell cycle windows (including G1 and G2/M phases) with quantifications and SDs is required to better depict cell cycle progression upon the different investigated conditions. Related to this, the title of the manuscript "S-phase AURORA A interacts with DICER and SETD2 to maintain cell cycle progression" is in my opinion overstated with respect to results as described in the actual form.*

Response: In response to this comment, we have made several changes to the gating strategy and presentation of the FACS experiments in **Figure 1D** and **Figure 4E** (previous Figure 4D). First, we have gated the BrdU-incorporating cells as suggested by the reviewer, considering all cells with a signal above the BrdU-negative control as positive (green population in the revised Figures). Second, the changes in the populations of G1, S-phase/BrdU-positive, S-phase/BrdU-negative, and G2/M are now shown as bar graphs with standard deviations from three biologically independent replicates in additional Figures (**Figure EV1C** and **Figure EV4F**). Moreover, the gating strategy for both experiments is explicitly illustrated (new **Figure EV1B**).

We also agree that the original title slightly overstated the conclusions. Therefore, we have revised the title from "S-phase AURORA A interacts with DICER and SETD2 to maintain cell

cycle progression" to "AURORA A interacts with DICER and SETD2 and promotes S-phase progression" in the revised manuscript."

> ***#2-2:*** *Interaction between AurkA and Dicer by PLA is interesting. Are positive cells in S-phase? Is the interaction increased upon replication stress? Is it impaired by using the mutants described in Figure 3, thus supporting the identified regions of interaction? Can these mutants be used, in parallel to AurkA PROTACs, in functional assays to show the relevance of the Aurora-A/Dicer interaction in resolving R-loops (e.g. R-loop detection using specific antibodies to see their increase)?*

Response: While technically challenging, we agree with the reviewer that it is important to assess whether proximity between DICER and AURORA A can be detected in S-phase cells. First, we began by analyzing the cell cycle distribution of U2OS cells using an immunofluorescence-based approach. To this end, we labelled DNA synthesis with a 2-hour EdU pulse, followed by click chemistry-based fluorophore conjugation to detect the incorporated EdU. We successfully combined the PLA procedure with DNA synthesis (EdU) and DNA content (Hoechst) staining. Cells were then plotted in an EdU versus Hoechst dot plot and colored based on their PLA signal. We observed that many cells in S-phase, but not in G1, are PLA-positive (new **Figures 4D** and **EV4D**; see also Reviewer **Figure R2A**). We also detected PLA-positive cells in G2/M, even at higher frequencies than in S-phase. However, AURORA A expression levels are considerably higher in G2 compared to S-phase, as addressed in response to comment #2-9 (new **Figure EV4A**). Based on these results, we conclude that AURORA A and DICER are indeed in close proximity in S-phase cells, and we have incorporated these new data into the revised manuscript.

Second, we repeated this experiment upon treatment with etoposide (20 µM, 4 hours), as suggested by the reviewer. As expected, etoposide treatment drastically reduced EdU incorporation but did not significantly increase the proximity between DICER and AURORA A (see **Reviewer Figure R2B**).

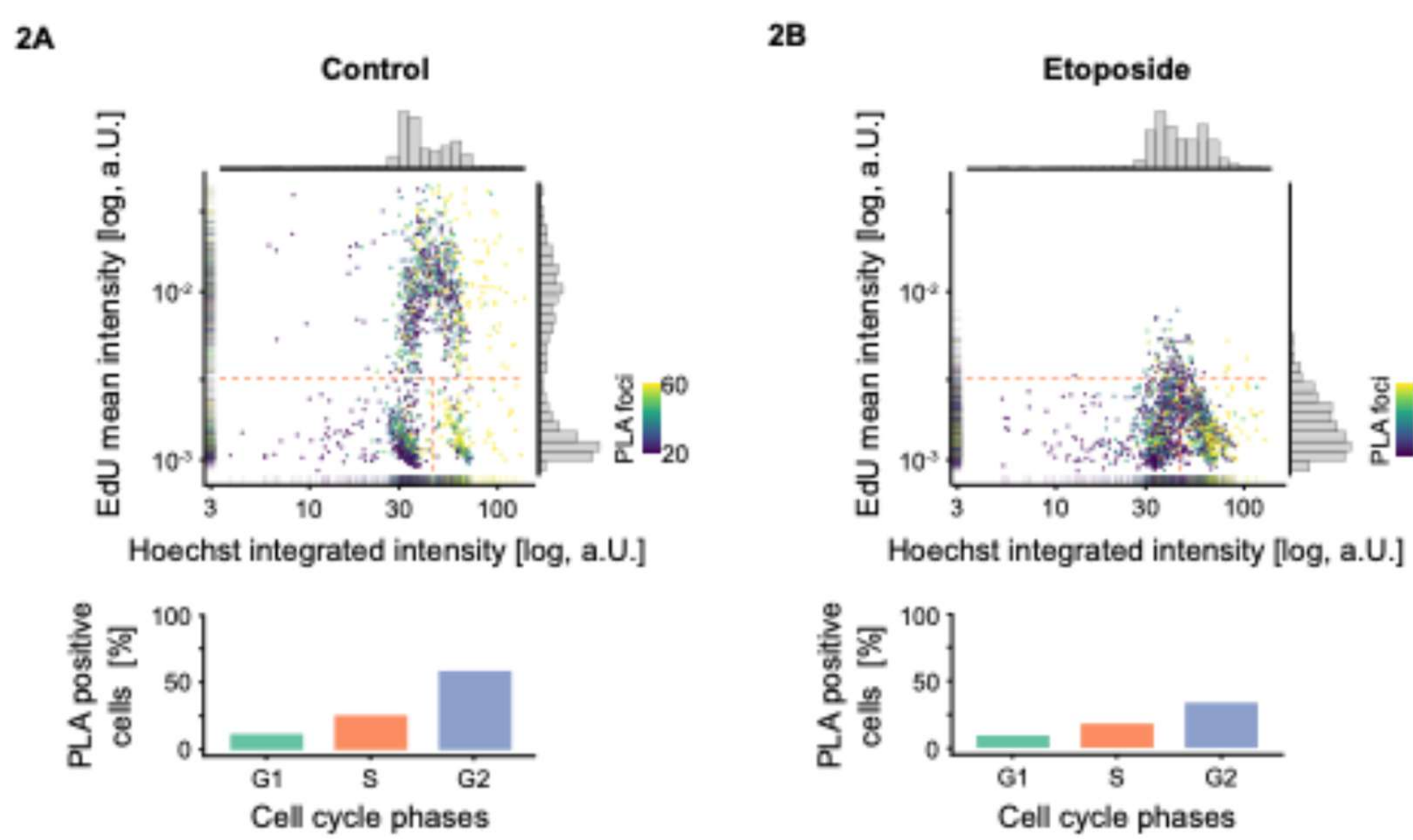

Figure R2. Cell cycle resolved analysis of DICER/AURORA A PLA in U2OS cells treated ± Etoposide. Scatter plot (top panel) shows individual nuclei as dots plotted according to DNA content (Hoechst integrated intensity, log scale) and DNA synthesis (EdU mean intensity, log scale) on the x- and y-axis, respectively, enabling cell cycle assignment. Dots were colored according to the number of PLA foci per nucleus. The color scale was limited from 20 to 60 PLA foci, with values outside this range squished to the nearest limit. Marginal histograms represent the distribution of Hoechst and EdU intensity. Bar plot (bottom panel) shows the percentage of PLA-positive nuclei (defined as nuclei with > 50 PLA dots) quantified per cell cycle phase. (**A**) and (**B**) panels show Control and

Etoposide-treated (20 µM, 4h) cells, respectively. An equal number of nuclei were subsampled per condition (n=1500).

We also appreciated the reviewer's suggestion to test the cellular proximity between AURORA A and the interaction-deficient DICER mutant identified in our study. Accordingly, we performed PLA experiments between AURORA A and an HA-tagged DICER mutant. Initially, we attempted to use an HA antibody for the PLA; however, this resulted in high background signals even in the absence of a second PLA antibody. As the PLA setup worked reliably with a DICER-specific antibody, we therefore generated a DICER knockout cell line (new **Figure EV5H**) and re-expressed either wild-type or mutant DICER (Δ L1058-R1288) via transient transfection. Subsequent PLA experiments using AURORA A and DICER antibodies again showed a strong increase in PLA signal compared to single-antibody control conditions (new **Figure EV4D**). Notably, the PLA signal was markedly reduced when the interaction-deficient DICER mutant was expressed instead of wild-type DICER. These results are described and discussed in the revised manuscript (new **Figure EV4D**).

We also aimed to analyze the functional consequences of an impaired AURORA A/DICER interaction and therefore performed immunofluorescence experiments using the R-loop–detecting antibody S9.6. However, we observed that the S9.6 antibody prominently stains cytoplasmic structures in U2OS cells, both after methanol and PFA fixation (Reviewer **Figure R3**). Therefore, we refrained from conducting further quantitative analyses using this antibody.

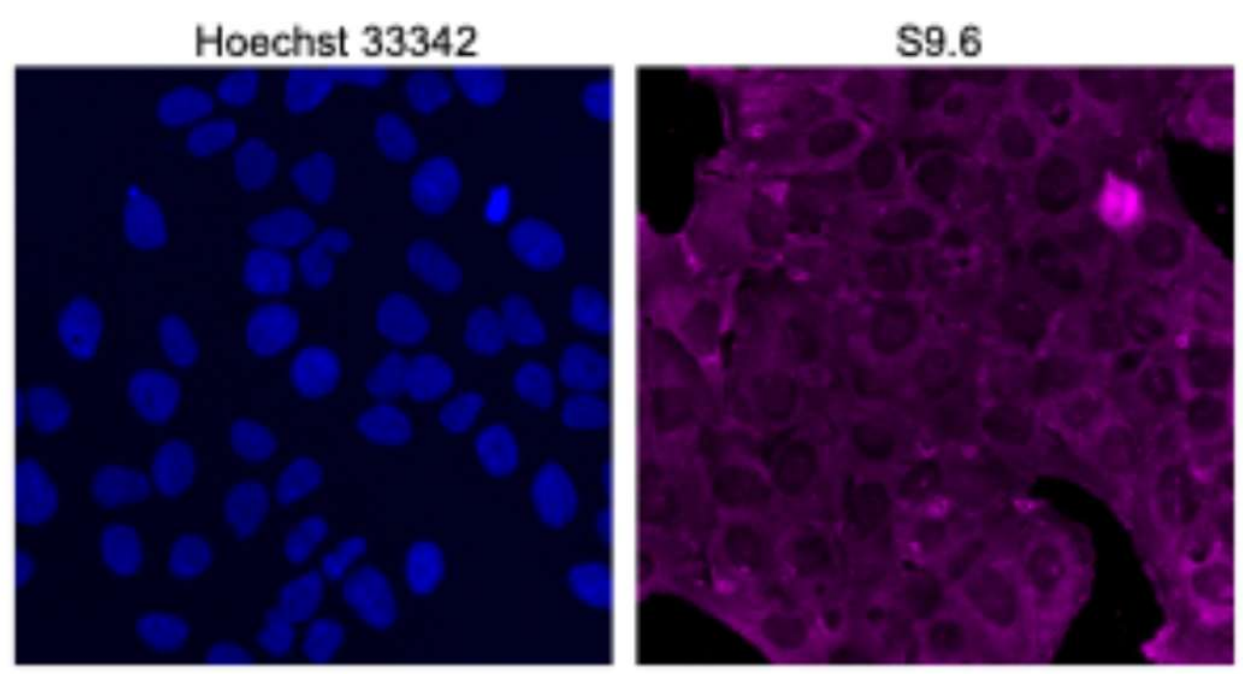

Figure R3. Immunofluorescence of DICER and R-loops in U2OS cells. Overview of immunofluorescence with the rabbit-S9.6 antibody (magenta, λ647) in HOECHST-stained cells (blue, λ405).

> ***#2-3:*** *The SETD2/AurkA interaction and the possibility of a ternary complex SETD2/AurkA/Dicer are interesting but have not been pursued. Therefore, additional experimental evidence is required in my opinion to validate some of the assumptions of the proposed model: can authors demonstrate an AurkA/SETD2 interaction in cells (e.g. by PLA)? And can they demonstrate Dicer/SETD2 interaction? If so, then the model would predict that AurkA PROTAC, and/or AurkA and Dicer mutants described before, would impair the interaction, which should be quite feasible to demonstrate. And, if the complex mediates SETD2 recruitment, do authors observe defective recruitment by silencing DICER, since they have the experimental setting in place?*

Response: We appreciated the reviewer's suggestion to perform PLA experiments between DICER and SETD2, and in particular to test the impact of PROTAC-mediated AURORA A depletion on this interaction. We made extensive efforts to establish a robust PLA for DICER and SETD2 across multiple independent experiments. However, the PLA signal remained only marginally above background levels, with the SETD2 antibody in particular contributing to elevated background. Therefore, we prefer not to include these data in the manuscript (**Reviewer Figure R4**). Importantly, in our experience, negative PLA results do not exclude

cellular proximity, as the assay is highly sensitive to experimental conditions (e.g., blocking), which can markedly influence signal detection. We would also like to point out that we did not directly propose the formation of a ternary complex between AURORA A, DICER, and SETD2 in the original version of the manuscript. Instead, we explicitly acknowledged the possibility of direct RNA binding and recruitment of AURORA A. To further clarify this point, we have added the following statement to the Discussion: "Since we did not demonstrate ternary complex formation, the proposed dual-output model does not necessarily occur simultaneously." In addition, we have adjusted the model to position AURORA A as directly interacting with RNA.

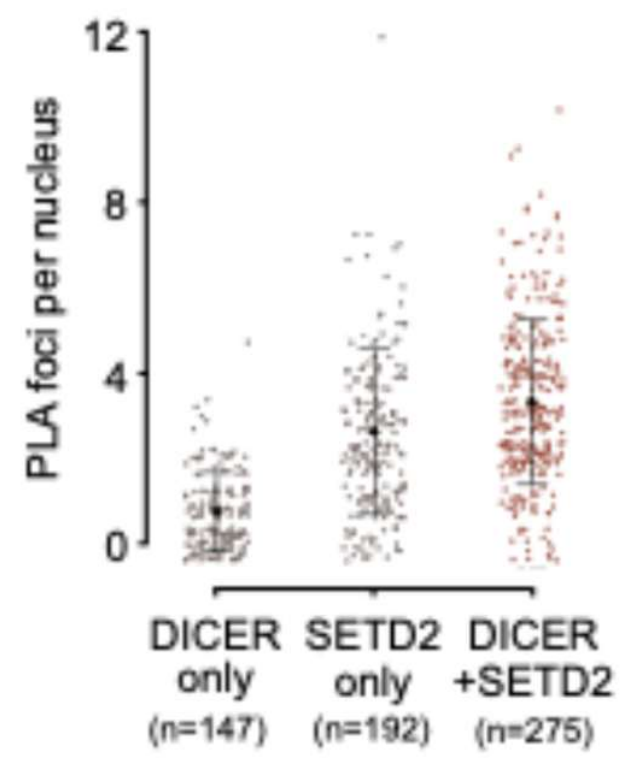

Figure R4. DICER/SETD2 PLA in U2OS cells. Jittered dot plot showing the number of PLA foci per nucleus for the combined PLA condition in U2OS cells and single-antibody controls (DICER only and SETD2 only). The number of nuclei analyzed per condition (n) is indicated below the corresponding condition.

To yet address the functional relevance of the interactions between AURORA A, DICER, and SETD2 further, we investigated whether depletion of DICER affects chromatin association of SETD2. To this end, we repeated the SETD2 CUT&RUN experiment in the DICER knockout cells generated in response to reviewer comment #2-2, performing the analysis in parallel with parental cells in biological triplicates. Strikingly, depletion of DICER nearly abolished chromatin association of SETD2. We have included these data in the revised manuscript, showing a broad genomic region (new **Figure 5K**) and a metagene analysis (new **Figure EV5H**).

Lastly, we are confident in the interaction between AURORA A and SETD2, as it has been consistently identified in multiple mass spectrometry experiments. To further support the interaction between AURORA A and SETD2, we have included a co-immunoprecipitation experiment in the revised manuscript (**new Figure EV5E**).

> ***#2-4:*** *Although the scaffolding role is intriguing, using AurkA inhibitors in some of the key experiments of the manuscript would strengthen the conclusion that kinase activity is not required. It is also possible that both scaffolding and phosphorylation-based effects exist, which is an interesting possibility. Authors also mention this case for SETD2, for which a phosphorylation by AurkA has been described with destabilizing effect. Since authors build a possible scenario in the Discussion for this dual effect, it would be interesting to insert this as a possible mechanism in the Model (Figure 6).*

Response: We like this comment! Degradation and inhibition of AURORA A cause distinct cell cycle phenotypes, demonstrating that AURORA A has scaffolding functions and that this function is required for S-phase progression. However, the reviewer is correct in pointing out that the scaffolding function of AURORA A might not be sufficient for S-phase progression, since PROTAC-mediated depletion of AURORA A abolishes both its scaffolding and catalytic

functions. One of the key mechanistic observations of this study is that PROTAC-mediated depletion of AURORA A causes specific proteins, such as SETD2, to shift towards lighter gradient fractions, indicating that AURORA A's scaffolding function could be to maintain these proteins in high-molecular-weight complexes. As suggested by the reviewer, we tested whether inhibition of AURORA A has similar effects on the migration behavior of these proteins.

We treated MV4-11 cells with the catalytic inhibitor MK-5108 (150 nM, 6 h), performed gradient centrifugation, and analyzed protein composition in each gradient fraction by mass spectrometry, as done previously for the PROTAC treatment. Of the 126 proteins that strongly shift towards lighter fractions upon PROTAC treatment (log2FC > 4), only 5 show a comparable shift upon MK-5108 treatment (new **Figure EV5D**). We then specifically analyzed the distribution of AURORA A and SETD2 across gradient fractions by immunoblotting and observed no substantial shift of either protein upon MK-5108 treatment compared to control conditions (new **Figure EV5B, C**). We have included and discussed these new data, which highlight the distinction between catalytic inhibition and degradation of AURORA A.

Finally, we have added the possibility of SETD2 phosphorylation to the model, as suggested by the reviewer.

> ***#2-5:*** *p. 5. If from 117 interactors 10 non-substrates were removed, why the remaining ones are 106? Maybe AurkA itself makes the difference, but I find the text as it is slightly confusing.*

Response: You are right, we detected 117 proteins in the AUROA A pull down and one of them is AURORA A itself. We rephrased this part now hopefully avoiding confusion.

> ***#2-6:*** *Legend 1G reads "as in 1G", I guess it should be "as in 1F".*

Response: We corrected the cross reference.

> ***#2-7:*** *From data shown in Fig. 1H TPX2 appears as a non RNA-mediated interaction, which is different from what was observed by Rajagopal et al., 2025. It would be interesting to comment on this cell cycle difference in the Discussion.*

Response: We agree and included a comment in the discussion ("A shift in the gradient migration behavior of AURORA A after RNase treatment was recently also demonstrated in cells synchronized in mitosis (Rajagopal et al., 2025), although with slight differences, as the migration behavior of AURORA A binding partner TPX2 is RNase sensitive in mitosis but not in S-phase.").

> ***#2-8:*** *p. 6. "These findings indicated that AURORA A assembles into extensive protein-protein complexes with RNA-binding proteins during S-phase, many of which have not been previously identified as substrates of AURORA A in phosphoproteomic analyses". I feel this is overstated before showing results coming in the following figures. At this stage I do not find that interacting with RBPs and existing in large complexes necessarily means that the large complexes include RBPs.*

Response: We removed "with RNA-binding proteins" in the sentence.

> ***#2-9:*** *Figure 4. AurkA is cell cycle regulated. Why are all cells in the IF panels positive and nuclear? This would be expected only in a G2-enriched culture. Is this the case?*

Response: As the reviewer points out, AURORA A is a protein whose levels are regulated throughout the cell cycle, with almost exclusive expression in G2/M in untransformed cells and significant expression throughout the entire cell cycle, albeit with peak levels in G2/M, in

many cancer cells (Nikonova *et al*, 2013) . To analyze AURORA A expression during the cell cycle in MV4-11 cells, we repeated the immunofluorescence experiment, this time in cells that were pulsed for one hour with the nucleoside analogue EdU, followed by staining of EdU-positive cells via click-chemistry-mediated fluorophore attachment. This setup allows the classification of each cell into distinct cell cycle phases. As expected, AURORA A expression is highest in G2, but it is also detectable in G1 and, to a yet lower degree, in S-phase (new **Figure EV4A**). We would like to point out that, despite the low expression levels of AURORA A in S-phase, we were able to detect a substantial number of proximity dots between AURORA A and DICER in this cell cycle phase, suggesting that this interaction may peak in S-phase. We have included this experiment in the revised manuscript.

> ***#2-10:*** *Figure 4C: add number of analysed cells per condition.*

Response: We added the number of analyzed cells to the figure legend.

> ***#2-11:*** *"Of all proteins identified in the S-phase interactome, 12 showed a significant shift toward lighter fractions upon RNase treatment (Fig. 2E). Among these were 8 RNA- binding proteins, including DICER, HNRNPA2B1, FXR1, FXR2 YLPM1 and XRCC5 (Fig. 2F)." It is not clear to me the correspondence between this sentence and Figure panels: if I look at the 12 significant factors in Figure 2E, RBPs are more than 8. On the other hand, not all proteins listed in the second sentence are included in Figure 2E. Can authors better explain this point?*

Response: We thank the reviewer for pointing this out. As Reviewer 1 (comment #1–5) raised a similar point, we provide here the same response: "In Figure 2E, we showed only the AURORA A interactors identified in the first proteomics experiment, in which AURORA A was immunoprecipitated from S-phase–synchronized cells without additional treatment. HNRNPA2B, FXR2, and XRCC5 were not among the 116 AURORA A interactors when applying stringent cut-off criteria in this experiment, but they were detected as interactors in the experiment directly comparing the effects of RNase treatment on AURORA A interactions. Although the proteomics results across all experiments are highly consistent, minor statistical differences can result in the inclusion or exclusion of a few proteins between independent experiments.

To avoid confusion for the reader, we have replaced the three candidates with SMNDC1, EXOSC2 and ELAVL1 in **Figure 2F** in the revised version, and we continue to focus on the initially described set of 116 interactors in the Results section. Furthermore, we have now made the data from the independent proteomics experiments accessible by including supplementary tables for each mass spectrometry experiment (**Dataset EV1, EV2, EV3**), in addition to uploading the raw data to the PRIDE repository (PXD076528: reviewer_pxd076528@ebi.ac.uk, password: tfnVpLbO2yqX and PXD069843: reviewer_pxd069843@ebi.ac.uk, password: ObDsbg25Pr0h)".

> ***#2-12:*** *p.11. We next investigated whether distinct gene subsets differing in expression strength, length, transcription factor binding sites or chromatin status were particularly affected by SETD2 loss. Strikingly, this analysis revealed a strong association between R-loops and SETD2 chromatin binding." Are the analyses indicated in the first sentence "not shown"?*

Response: We analyzed these parameters but did not observe a notable correlation with SETD2 loss. We chose not to present these negative data, as they could be distracting. Therefore, we have removed the phrase "differing in expression strength, length,

transcription factor binding sites, or chromatin status” from the sentence. Thank you once again for your careful review of our manuscript and the very constructive comments!

Reviewer #3

> ***General comments**: Considering these results together, this work stands to significantly advance the field by extensive characterization of AURORA A outside of its catalytic activity, with potentially far-reaching consequences for pathologies. Overall, the manuscript is well-prepared and offers appropriate interpretations of the presented data. Below, we suggest minor revisions.*

Response: We thank the reviewer for their positive assessment of the significance of our work and for their highly constructive and helpful comments.

> ***#3-1:** Because the proposed mechanism appears R-loop associated, a deeper analysis of the existing dataset with RNase H treatment may be warranted.*

Response: We agree with the reviewer that the role of R-loops in the function of the S-phase AURORA A complex is an interesting question and warrants further investigation, e.g. by using R-loop–specific RNase H. Accordingly, we have performed additional analyses as well as an additional experiment. First, we analyzed published datasets for R-loop–interacting proteins, including those identified by Mosler et al. using the R-loop–binding domain of RNase H (Mosler *et al.*, 2021), by Cristini et al. using the S9.6 R-loop antibody (Cristini *et al.*, 2018), and by Wang et al. using a synthetic hybrid R-loop–binding protein (Wang *et al.*, 2018). Interestingly, many AURORA A–binding proteins were present in these datasets. These results have been incorporated into the revised manuscript as Venn diagrams (**Figure EV5I**) and a table (**Dataset EV4**).

Second, we investigated whether SETD2 binding to specific chromatin regions is dependent on R-loops. As already shown in the original version of the manuscript by CUT&RUN experiments, PROTAC-mediated degradation of AURORA A reduced SETD2 binding to genic regions. We have now repeated the CUT&RUN experiment in cells pretreated with recombinant RNase H. Remarkably, we observed a complete loss of SETD2 binding to chromatin, as demonstrated across a large genomic region (new **Figure 5K**) and by metagene analysis (new **Figure EV5I**) of highly SETD2-bound genes. We thank the reviewer for suggesting this experiment and believe that this strong global correlation between DICER, R-loops and SETD2 is intriguing and may open up new avenues for future studies.

> ***#3-2:** Does degrading AURORA A with PROTAC cause its interacting partners to be destabilized? This could be addressed by comparing mass spectrometry datasets in AURORA A positive versus depleted cells.*

Response: We analyzed the levels of AURORA A interaction partners following PROTAC-mediated degradation, as suggested by the reviewer. Of the 116 identified interactors, 94 were detected in the lysates of MV4-11 cells, and of these, none was destabilized, 93 remained unchanged, and 1 was increased. These data are presented in the new **Figure EV1E** in the revised manuscript.

> ***#3-3:** The sucrose gradient fractionation in Figure 1J and Figure 2B should be accompanied by the UV trace of these experiments to demonstrate the statement on p6 "corresponding to approximately 80S" - perhaps as a separate plot in the supplement. Additionally, Figure 1J's plot is stretched horizontally, distorting the points and error bars.*

Response: We measured absorbance at 260 nm and observed an RNase-sensitive peak at fraction 18, which coincides with the AURORA A peak. To further support that this fraction contains monosomes, we plotted RPL9 and RPS7 from the gradient-MS experiment and

observed peaks at similar fractions, with a pre-peak at fraction 14 for RPS17 (likely reflecting the small ribosomal subunit) and a shoulder around fraction 15 for RPL9 (likely reflecting the large ribosomal subunit). We did not detect polysomes, as our centrifugation conditions led to their precipitation; we centrifuged for 18 hours, whereas polysome profiling typically requires 2–3 hours under otherwise similar conditions (Han *et al*, 2022; Pearl *et al*, 2020). We have included the new analyses in **Figure EV1G** and refer to this figure when describing the migration behavior of AURORA A in the revised manuscript.

> ***#3-4:*** *p6 lines 3-5: Expressing C-terminally and N-terminally HA-tagged AURORA A may not constitute four biological replicates if a population of AURORA A is proteolytically cleaved before analysis, or if the tags interfere with different interactors. Thus, analyzing both tags makes the conclusions more robust, but an additional sentence or a change in wording may be warranted.*

Response: We fully agree and have removed the phrase "four biological replicates."

> ***#3-5:*** *The authors should consider citing [PMID:41161313] and [PMID:35194019] which indicate a role for dicer in R-loop management and AURORA B function, respectively.*

Response: Thank you for making us aware of these interesting papers! We quote the Cheng et al paper now when mentioning Dicers nuclear functions first (page 5), when describing the involvement of Dicer in R-loop resolution (page 9) and in the discussion (page 14) (Cheng *et al*, 2025). We also mention the synthetic effects of DICER knock out and overexpressing and silencing Aurora B as found by Gutbrod et al in the discussion (Gutbrod *et al*, 2022) (page 14).

> ***#3-6:*** *It is interesting that fractions 8-12 lack AURORA A complexes, forming two regimes that agree with the authors' later "light" versus "heavy" analyses. Can the authors speculate why the variations in complexes skip the middle fractions, such as association with a specific larger element?*

Response: While further analyses, such as immunoprecipitations from gradient fractions, are required to fully characterize the nature of the two AURORA A fractions, we consider it likely that the second fraction sediments in the heavier gradient fractions due to its RNA-dependent association with chromatin. We have included this possibility in the Discussion.

> ***#3-7:*** *While this manuscript is well-prepared, there are a few minor grammatical inconsistencies:*
>
> *- p3 line 12: "well-characterized" is hyphenated elsewhere, but not here.*
>
> *- p4 lines 4-5: this clause should use two em dashes-for example, like this-rather than hyphens (also: p10 line 8, third to last line of p12, p13 line 14, and p14 line 10).*
>
> *- p7 second to last line: "recruit" rather than "recruited".*
>
> *- p10 third to last line: "high-molecular-weight" is not hyphenated previously.*
>
> *- p10 last line and later on p11: CUT&RUN is an acronym and should be capitalized.*
>
> *- p30 under (G), "RNAse" should be "RNase".*
>
> *- p31 under (J) "the highest signal was set to "1" should reflect the y-axis significant figures with "1.0".*
>
> *- p31 under (C) (and later for (Figure 3A)) high-molecular-weight should be hyphenated for consistency.*
>
> *- p31 under (Figure 3A) "overlayed" should be "overlaid".*

- p32 under (E), missing a space between "AURORAA".

- p33 under (C) "3 independent experiments" - "three" is spelled out in previous captions when referring to replicates (again on p36 under Figure EV4 A).

- p35 under (Figure EV1 C) "Triazole" should be "trizol" or (branded) "TRIzol".

Response: We have corrected these errors and thank the reviewer again for the excellent comments and help!

References

Cheng T, Roche B, Abderahmane F, Touat-Todeschini L, Freon K, Lakhani AA, Bhattacharjee S, Spielmann LG, Jenen E, Choi C *et al* (2025) Transcription-replication conflict resolution by nuclear RNA interference. *Molecular cell* 85: 3930–3946 e3935

Cristini A, Groh M, Kristiansen MS, Gromak N (2018) RNA/DNA Hybrid Interactome Identifies DXH9 as a Molecular Player in Transcriptional Termination and R-Loop-Associated DNA Damage. *Cell Rep* 23: 1891–1905

Gutbrod MJ, Roche B, Steinberg JI, Lakhani AA, Chang K, Schorn AJ, Martienssen RA (2022) Dicer promotes genome stability via the bromodomain transcriptional co-activator BRD4. *Nature communications* 13: 1001

Han C, Sun L, Pan Q, Sun Y, Wang W, Chen Y (2022) Polysome profiling followed by quantitative PCR for identifying potential micropeptide encoding long non-coding RNAs in suspension cell lines. *STAR Protoc* 3: 101037

Mosler T, Conte F, Longo GMC, Mikicic I, Kreim N, Mockel MM, Petrosino G, Flach J, Barau J, Luke B *et al* (2021) R-loop proximity proteomics identifies a role of DDX41 in transcription-associated genomic instability. *Nature communications* 12: 7314

Nikonova AS, Astsaturov I, Serebriiskii IG, Dunbrack RL, Jr., Golemis EA (2013) Aurora A kinase (AURKA) in normal and pathological cell division. *Cell Mol Life Sci* 70: 661–687

Pearl D, Katsumura S, Amiri M, Tabatabaei N, Zhang X, Vinette V, Pang X, Beug ST, Kim SH, Jones LM *et al* (2020) 4E-BP-Dependent Translational Control of Irf8 Mediates Adipose Tissue Macrophage Inflammatory Response. *J Immunol* 204: 2392–2400

Wang IX, Grunseich C, Fox J, Burdick J, Zhu Z, Ravazian N, Hafner M, Cheung VG (2018) Human proteins that interact with RNA/DNA hybrids. *Genome research* 28: 1405–1414

1st Revision - Editorial Decision 2nd Jun 2026

Dear Prof. Wolf,

Thank you for the submission of your revised manuscript to our editorial offices. I have now received the reports from the three referees that were asked to re-evaluate the study, you will find below. As you will see, the referees now support the publication of the study in EMBO reports. Referee #2 has remaining minor concerns and suggestions to improve the manuscript I ask you to address in a final revised version. Please also provide a final p-b-p-response to these points and the editorial requests below.

Editorial requests:

- Please provide the abstract written in present tense throughout.

- Please order the manuscript sections like this, using only these names:
Title page - Abstract - Keywords - Introduction - Results - Discussion - Methods - Data availability section - Acknowledgements - Disclosure and Competing Interests Statement - References - Figure legends - Expanded View Figure legends

- Please remove the 'Expanded View Dataset Legends' from the main manuscript text file. Please make sure that their title and legend are provided on the first TAB of each dataset excel file.

- Please remove now the referee access information from the Data Availability section, provide direct links to all datasets and make sure the datasets are public latest upon online publication of the study. Moreover, please remove any text from this section not related to externally deposited datasets.

- Please add the journal name, the manuscript ID# and the corr. author's name to the author checklist.

- Please check again that the number "n" for how many independent experiments were performed, their nature (biological versus technical replicates), the bars and error bars (e.g. SEM, SD) and the test used to calculate p-values is indicated in the respective figure legends (main and EV figures). Please also check that all the p-values are explained in the legend, and that these fit to those shown in the figure. Please provide statistical testing where applicable. Please avoid the phrase 'independent experiment' but clearly state if these were biological or technical replicates. Please also indicate (e.g. with n.s.) if testing was performed, but the differences are not significant. In case n=2, please show the data as separate datapoints without error bars and statistics. See also:

https://link.springer.com/journal/44319/submission-guidelines#cms-Figure-and-data-presentation

If n<5, please show single datapoints for diagrams. Moreover:
- Please note that the exact p values are not provided in the legends of 1D; 4E.
- Please indicate the statistical test used for data analysis in the legends of figure 4C.
- Please note that the box plots need to be defined in terms of minima, maxima, centre, bounds of box and whiskers, and percentile in the legends of figures 5I,J; EV-4D.
- Please note that the box plots need to be defined in terms of minima, maxima, and whiskers in the legends of figure EV-4C.
- Please note that information related to n is missing in the legends of 1B.

- All Materials and Methods need to be described in the main text using our 'Structured Methods' format, which is required for all research articles. According to this format, the Methods section should include a Reagents and Tools Table (listing key reagents, experimental models, software, and relevant equipment and including their sources and relevant identifiers), uploaded as separate file, and a Methods section in which we encourage the authors to describe their methods using a step-by-step protocol format with bullet points, to facilitate the adoption of the methodologies across labs. More information on how to adhere to this format as well as downloadable templates (.doc) for the Reagents and Tools Table can be found in our author guidelines (section 'Structured Methods'):

https://link.springer.com/journal/44319/submission-guidelines#cms-Manuscript-organisation-and-formatting

- Please check the right GAPDH panels in Figs. 3G, 3H and EV5E. Was there any signal detected; at least background? Currently, what is shown seems blank. Could you mark in the provided source data the film and the region that is shown in the figure panels (see below)?

- Please indicate in the source data for the Western blots with a square the region that is shown in the final figure panels. Moreover, please confirm that for all Western blot panels shown the loading control was run on the same gel as the other proteins detected. Please note that we discourage comparisons between samples on different gels/blots, even if the samples derive from one experiment, as confounding factors reduce comparability. If unavoidable, the figure legend must state that the samples derive from the same experiment and that gels/blots were processed in parallel. If a 'representative' loading control is shown for multiple gels/blots, the intra-gel controls should be shown in the source data files, and the figure legends should

describe the data displayed accurately. See our author guidelines:

https://link.springer.com/journal/44319/submission-guidelines#cms-Figure-and-data-presentation (section 'Electrophoretic gels and blots').

In addition, I would need from you uploaded separately:
- a short, two-sentence summary of the manuscript (not more than 35 words).
- two to four short (!) bullet points highlighting the key findings of your study (two lines each).
- a schematic summary figure as separate file that provides a sketch of the major findings (not a data image) in jpeg or tiff format (with the exact width of 550 pixels and the exact height of 300 pixels) that can be used as a visual synopsis on our website. Please upload this separately and remove the graphical abstract from the manuscript text file.

I look forward to seeing a new revised version of your manuscript as soon as possible.

Best,

Achim Breiling
Senior Editor
EMBO Reports

Referee #1:

The authors have successfully addressed all of the concerns raised in my original review. In the revised manuscript, they have incorporated additional data and made appropriate revisions that further support their conclusions. I agree with the authors that immunostaining with the S9.6 antibody can, in many cases, be problematic due to cytoplasmic staining, which may lead to ambiguous interpretation. Therefore, omitting these data to avoid confusion is a reasonable decision. Overall, the revised manuscript has been significantly improved and is now suitable for publication.

Referee #2:

In the revised version of the manuscript authors have answered most of raised comments, performing new experiments and adding interesting novel data. I find this significantly improves the quality of the work, it strengthens the results and widens the significance of the conclusions. I list below only a couple of minor points related to comment 2.1 that in my opinion should be clarified before publication.

Related to previous comment 2.1.
p.5 "MV4-11 cells undergo S-phase arrest upon AURORA A degradation, whereas catalytic inhibition of AURORA A resulted in a
more pronounced G2/M arrest....(Fig. 1D, EV1B, C). "

Although it is clear from histograms and statistical analysis in Fig. 1D that S-phase progression, monitored by EdU incorporation, is impaired with PROTACs and not with Alisertib, it is less clear from data in EV1C that catalytic inhibition results in a more pronounced G2/M arrest. Indeed, G2/M cells upon the 3 treatments appear quite similar and no statistical analysis is reported. I would modify the text accordingly.

p.10 "Subsequent BrdU-PI FACS experiments demonstrated that the combined depletion of both proteins induced a rampant S-phase arrest, even slightly more pronounced than that caused by JB301 treatment alone or the mild arrest observed upon DICER depletion only (Fig. 4E, EV4F). "

Again, it does not appear to be a significant difference between JB301 and dox+JB301, particularly in EV4F. If the minor difference in 4E is significant this should be shown by direct statistical comparison, otherwise I would rephrase the text.

Referee #3:

The authors have comprehensively addressed all of the reviewers' comments.

Response to the reviewers

First of all, we would like to thank the reviewers for their support and careful review of our revised manuscript, and the editor for their guidance, advice, and valuable comments. Please find below our responses to each point raised, together with the corresponding changes made to the manuscript.

Reviewer #1

> ***General comment:*** *The authors have successfully addressed all of the concerns raised in my original review. In the revised manuscript, they have incorporated additional data and made appropriate revisions that further support their conclusions. I agree with the authors that immunostaining with the S9.6 antibody can, in many cases, be problematic due to cytoplasmic staining, which may lead to ambiguous interpretation. Therefore, omitting these data to avoid confusion is a reasonable decision. Overall, the revised manuscript has been significantly improved and is now suitable for publication.*

Response: Thank you for your time and support!

Reviewer #2

> ***General comment:*** *In the revised version of the manuscript authors have answered most of raised comments, performing new experiments and adding interesting novel data. I find this significantly improves the quality of the work, it strengthens the results and widens the significance of the conclusions. I list below only a couple of minor points related to comment 2.1 that in my opinion should be clarified before publication.*

Response: We agree with your suggested text edits and have revised the sentences accordingly. Thank you for your time and support.

> ***#2-1:*** *Related to previous comment 2.1.p.5 "MV4-11 cells undergo S-phase arrest upon AURORA A degradation, whereas catalytic inhibition of AURORA A resulted in a more pronounced G2/M arrest....(Fig. 1D, EV1B, C). " Although it is clear from histograms and statistical analysis in Fig. 1D that S-phase progression, monitored by EdU incorporation, is impaired with PROTACs and not with Alisertib, it is less clear from data in EV1C that catalytic inhibition results in a more pronounced G2/M arrest. Indeed, G2/M cells upon the 3 treatments appear quite similar and no statistical analysis is reported. I would modify the text accordingly.*

Response: We agree and have revised the sentence by removing the phrase “more pronounced”.

> ***#2-1:*** *p.10 "Subsequent BrdU-PI FACS experiments demonstrated that the combined depletion of both proteins induced a rampant S-phase arrest, even slightly more pronounced than that caused by JB301 treatment alone or the mild arrest observed upon DICER depletion only (Fig. 4E, EV4F)." Again, it does not appear to be a significant difference between JB301 and dox+JB301, particularly in EV4F. If the minor difference in 4E is significant this should be shown by direct statistical comparison, otherwise I would rephrase the text.*

Response: We agree and have revised the sentence by removing the part “, even slightly more pronounced than that caused by JB301 treatment alone or the mild arrest observed upon DICER depletion only”.

Reviewer #3

> ***General comment:*** *The authors have comprehensively addressed all of the reviewers' comments.*

Response: Thank you for your time and support!

Editor

> ***Editor-1:*** *Please provide the abstract written in present tense throughout.*

Response: We have changed the verbs in the abstract to the present tense.

> ***Editor-2:*** *Please order the manuscript sections like this, using only these names: Title page - Abstract - Keywords - Introduction - Results - Discussion - Methods - Data availability section - Acknowledgements - Disclosure and Competing Interests Statement - References - Figure legends - Expanded View Figure legends*

Response: We ordered the manuscript accordingly.

> ***Editor-3:*** *Please remove the 'Expanded View Dataset Legends' from the main manuscript text file. Please make sure that their title and legend are provided on the first TAB of each dataset excel file.*

Response: We removed this section.

> ***Editor-4:*** *Please remove now the referee access information from the Data Availability section, provide direct links to all datasets and make sure the datasets are public latest upon online publication of the study. Moreover, please remove any text from this section not related to externally deposited datasets.*

Response: We removed the referee access information.

> ***Editor-5:*** *Please add the journal name, the manuscript ID# and the corr. author's name to the author checklist.*

Response: We revised the author check list accordingly.

> ***Editor-6:*** *Please check again that the number "n" for how many independent experiments were performed, their nature (biological versus technical replicates), the bars and error bars (e.g. SEM, SD) and the test used to calculate p-values is indicated in the respective figure legends (main and EV figures). Please also check that all the p-values are explained in the legend, and that these fit to those shown in the figure. Please provide statistical testing where applicable. Please avoid the phrase 'independent experiment' but clearly state if these were biological or technical replicates. Please also indicate (e.g. with n.s.) if testing was performed, but the differences are not significant. In case n=2, please show the data as separate datapoints without error bars and statistics. See also: https://link.springer.com/journal/44319/submission-guidelines#cms-Figure-and-data-presentation If n<5, please show single datapoints for diagrams.*

Response: We went through the manuscript once more and added the requested missing statistical descriptions.

> ***Editor-7:*** *Please note that the exact p values are not provided in the legends of 1D; 4E.*

Response: We added the exact p-values to the figure.

> ***Editor-8:*** *Please indicate the statistical test used for data analysis in the legends of figure 4C.*

Response: We used the Tukey's multiple comparison test and this information was already part of the previous figure legend.

> ***Editor-9:*** *Please note that the box plots need to be defined in terms of minima, maxima, centre, bounds of box and whiskers, and percentile in the legends of figures 5I,J; EV-4D.*

Response: We added a description of the box plots.

Editor-10: *Please note that the box plots need to be defined in terms of minima, maxima, and whiskers in the legends of figure EV-4C.*

Response: We added a description of the box plots.

Editor-11: *Please note that information related to n is missing in the legends of 1B.*

Response: We added the number and type of replicates.

Editor-12: *All Materials and Methods need to be described in the main text using our 'Structured Methods' format, which is required for all research articles. According to this format, the Methods section should include a Reagents and Tools Table (listing key reagents, experimental models, software, and relevant equipment and including their sources and relevant identifiers), uploaded as separate file, and a Methods section in which we encourage the authors to describe their methods using a step-by-step protocol format with bullet points, to facilitate the adoption of the methodologies across labs. More information on how to adhere to this format as well as downloadable templates (.doc) for the Reagents and Tools Table can be found in our author guidelines (section 'Structured Methods'): https://link.springer.com/journal/44319/submission-guidelines#cms-Manuscript-organisation-and-formatting*

Response: We added and uploaded a Reagents and Tools Table.

Editor-13: *Please check the right GAPDH panels in Figs. 3G, 3H and EV5E. Was there any signal detected; at least background? Currently, what is shown seems blank. Could you mark in the provided source data the film and the region that is shown in the figure panels (see below)?*

Response: The GAPDH-panel in the pulldown were indeed taken from the respective Westernblots which did not show a signal (and very little background). We indicated the respective area in the raw data.

Editor-14: *Please indicate in the source data for the Western blots with a square the region that is shown in the final figure panels. Moreover, please confirm that for all Western blot panels shown the loading control was run on the same gel as the other proteins detected. Please note that we discourage comparisons between samples on different gels/blots, even if the samples derive from one experiment, as confounding factors reduce comparability. If unavoidable, the figure legend must state that the samples derive from the same experiment and that gels/blots were processed in parallel. If a 'representative' loading control is shown for multiple gels/blots, the intra-gel controls should be shown in the source data files, and the figure legends should describe the data displayed accurately. See our author guidelines: https://link.springer.com/journal/44319/submission-guidelines#cms-Figure-and-data-presentation (section 'Electrophoretic gels and blots').*

Response: For each immunoblot, the source data directory contains a PDF file indicating, with highlighted boxes, the regions of the raw images that were selected for inclusion in the corresponding panel of the main figure. For example, for Figure 1A, this file is named "1A_WB_overview.pdf".

Editor-15: *In addition, I would need from you uploaded separately: - a short, two-sentence summary of the manuscript (not more than 35 words).*

Response: I uploaded a two-sentence summary.

Editor-16: *two to four short (!) bullet points highlighting the key findings of your study (two lines each).*

Response: I uploaded the requested bullet point summary.

Editor-17: *a schematic summary figure as separate file that provides a sketch of the major findings (not a data image) in jpeg or tiff format (with the exact width of 550 pixels and the exact height of 300 pixels) that can be used as a visual synopsis on our website. Please upload this separately and remove the graphical abstract from the manuscript text file.*

Response: We created and uploaded a graphical abstract. Thank you for your editorial guidance.

Prof. Elmar Wolf
University of Kiel
Institute of Biochemistry
Rudolf-Höber-Str. 1
Kiel 24118
Germany

Dear Prof. Wolf,

Thank you for the submission of your final revised manuscript to our editorial offices. I now went through this and your final p-b-p-response and consider the remaining points of referee #2 and the editorial requests as adequately addressed.

I am thus very pleased to accept your manuscript for publication in the next available issue of EMBO reports. Thank you for your contribution to our journal.

Your manuscript will be processed for publication by EMBO Press. It will be copy edited and you will receive page proofs prior to publication. Please note that you will be contacted by Springer Nature Author Services to complete licensing and payment information.

You may qualify for financial assistance for your publication charges - either via a Springer Nature fully open access agreement or an EMBO initiative. Check your eligibility: https://link.springer.com/journal/44319/how-to-publish-with-us

Should you be planning a Press Release on your article, please notify the production team via this form in order to coordinate publication and release dates: https://forms.cloud.microsoft/e/eH4A8JVP6g

If you have any questions, please do not hesitate to contact the Editorial Office. Thank you for your contribution to EMBO Reports.

Yours sincerely,

Achim Breiling
Senior Editor
EMBO Reports

>>> Please note that it is EMBO Reports policy for the transcript of the editorial process (containing referee reports and your response letter) to be published as an online supplement to each paper. If you do NOT want this, you will need to inform the Editorial Office via email immediately. More information is available here: https://link.springer.com/partners/embo-press/editorial-policies#Peer%20review

EMBO Press Author Checklist

Corresponding Author Name: Elmar Wolf
Journal Submitted to: EMBO Reports
Manuscript Number: EMBOR-2025-63072V2

USEFUL LINKS FOR COMPLETING THIS FORM
The EMBO Journal - Author Guidelines
EMBO Reports - Author Guidelines
Molecular Systems Biology - Author Guidelines
EMBO Molecular Medicine - Author Guidelines

Reporting Checklist for Life Science Articles (updated January
This checklist is adapted from Materials Design Analysis Reporting (MDAR) Checklist for Authors. MDAR establishes a minimum set of requirements in transparent reporting in the life sciences (see Statement of Task: 10.31222/osf.io/9sm4x). Please follow the journal's guidelines in preparing your
Please note that a copy of this checklist will be published alongside your article.

Abridged guidelines for figures
1. Data
The data shown in figures should satisfy the following conditions:
- the data were obtained and processed according to the field's best practice and are presented to reflect the results of the experiments in an accurate and unbiased manner.
- ideally, figure panels should include only measurements that are directly comparable to each other and obtained with the same assay.
- plots include clearly labeled error bars for independent experiments and sample sizes. Unless justified, error bars should not be shown for technical
- if n<5, the individual data points from each experiment should be plotted. Any statistical test employed should be justified.
- Source Data should be included to report the data underlying figures according to the guidelines set out in the authorship guidelines on Data

2. Captions
Each figure caption should contain the following information, for each panel where they are relevant:
- a specification of the experimental system investigated (eg cell line, species name).
- the assay(s) and method(s) used to carry out the reported observations and measurements.
- an explicit mention of the biological and chemical entity(ies) that are being measured.
- an explicit mention of the biological and chemical entity(ies) that are altered/varied/perturbed in a controlled manner.
- the exact sample size (n) for each experimental group/condition, given as a number, not a range;
- a description of the sample collection allowing the reader to understand whether the samples represent technical or biological replicates (including how many animals, litters, cultures, etc.).
- a statement of how many times the experiment shown was independently replicated in the laboratory.
- definitions of statistical methods and measures:
  - common tests, such as t-test (please specify whether paired vs. unpaired), simple χ2 tests, Wilcoxon and Mann-Whitney tests, can be unambiguously identified by name only, but more complex techniques should be described in the methods section;
  - are tests one-sided or two-sided?
  - are there adjustments for multiple comparisons?
  - exact statistical test results, e.g., P values = x but not P values < x;
  - definition of 'center values' as median or average;
  - definition of error bars as s.d. or s.e.m.

Please complete ALL of the questions below.
Select "Not Applicable" only when the requested information is not relevant for your study.

Materials

Newly Created Materials	Information included in the manuscript?	In which section is the information available? (Reagents and Tools Table, Materials and Methods, Figures, Data Availability Section)
New materials and reagents need to be available; do any restrictions apply?	Yes	Data availability section (page 26)

Antibodies	Information included in the manuscript?	In which section is the information available? (Reagents and Tools Table, Materials and Methods, Figures, Data Availability Section)
For **antibodies** provide the following information:<br>- Commercial antibodies: RRID (if possible) or supplier name, catalogue number and or/clone number<br>- Non-commercial: RRID or citation	Yes	Materials and Methods

DNA and RNA sequences	Information included in the manuscript?	In which section is the information available? (Reagents and Tools Table, Materials and Methods, Figures, Data Availability Section)
**Short novel DNA or RNA including primers, probes**: provide the sequences.	Yes	Table EV5

Cell materials	Information included in the manuscript?	In which section is the information available? (Reagents and Tools Table, Materials and Methods, Figures, Data Availability Section)
**Cell lines:** Provide species information, strain. Provide accession number in repository **OR** supplier name, catalog number, clone number, and/**OR** RRID.	Yes	Materials and Methods
**Primary cultures:** Provide species, strain, sex of origin, genetic modification status.	Not Applicable	
Report if the cell lines were recently **authenticated** (e.g., by STR profiling) and tested for mycoplasma contamination.	Yes	Materials and Methods, page 16

Experimental animals	Information included in the manuscript?	In which section is the information available? (Reagents and Tools Table, Materials and Methods, Figures, Data Availability Section)
**Laboratory animals or Model organisms:** Provide species, strain, sex, age, genetic modification status. Provide accession number in repository **OR** supplier name, catalog number, clone number, **OR** RRID.	Not Applicable	
**Animal observed in or captured from the field:** Provide species, sex, and age where possible.	Not Applicable	
Please detail **housing and husbandry conditions**.	Not Applicable	

Plants and microbes	Information included in the manuscript?	In which section is the information available? (Reagents and Tools Table, Materials and Methods, Figures, Data Availability Section)
**Plants:** provide species and strain, ecotype and cultivar where relevant, unique accession number if available, and source (including location for collected wild specimens).	Not Applicable	
**Microbes:** provide species and strain, unique accession number if available, and source.	Not Applicable	

Human research participants	Information included in the manuscript?	In which section is the information available? (Reagents and Tools Table, Materials and Methods, Figures, Data Availability Section)
If collected and within the bounds of privacy constraints report on age, sex and gender or ethnicity for all study participants.	Not Applicable	

Core facilities	Information included in the manuscript?	In which section is the information available? (Reagents and Tools Table, Materials and Methods, Figures, Data Availability Section)
If your work benefited from core facilities, was their service mentioned in the acknowledgments section?	Not Applicable	

Design

Study protocol	Information included in the manuscript?	In which section is the information available? (Reagents and Tools Table, Materials and Methods, Figures, Data Availability Section)
If study protocol has been **pre-registered, provide DOI in the manuscript**. For clinical trials, provide the trial registration number **OR** cite DOI.	Not Applicable	
Report the **clinical trial registration number** (at ClinicalTrials.gov or equivalent), where applicable.	Not Applicable	

Laboratory protocol	Information included in the manuscript?	In which section is the information available? (Reagents and Tools Table, Materials and Methods, Figures, Data Availability Section)
Provide DOI OR other citation details if **external detailed step-by-step protocols** are available.	Not Applicable	

Experimental study design and statistics	Information included in the manuscript?	In which section is the information available? (Reagents and Tools Table, Materials and Methods, Figures, Data Availability Section)
Include a statement about **sample size** estimate even if no statistical methods were used.	Yes	Materials and Methods, page 18
Were any steps taken to minimize the effects of subjective bias when allocating animals/samples to treatment (e.g. **randomization procedure**)? If yes, have they been described?	Not Applicable	
Include a statement about **blinding** even if no blinding was done.	Yes	Materials and Methods, page 18
Describe **inclusion/exclusion criteria** if samples or animals were excluded from the analysis. Were the criteria pre-established?<br><br>If sample or data points were omitted from analysis, report if this was due to attrition or intentional exclusion and provide justification.	Yes	Materials and Methods, page 22
For every figure, are **statistical tests** justified as appropriate? Do the data meet the assumptions of the tests (e.g., normal distribution)? Describe any methods used to assess it. Is there an estimate of variation within each group of data? Is the variance similar between the groups that are being statistically compared?	Yes	We chose justified statistical tests and describe the test performed in the respective figure legend

Sample definition and in-laboratory replication	Information included in the manuscript?	In which section is the information available? (Reagents and Tools Table, Materials and Methods, Figures, Data Availability Section)
In the figure legends: state number of times the experiment was **replicated** in laboratory.	Yes	Figure legends
In the figure legends: define whether data describe **technical or biological replicates**.	Yes	Figure legends

Ethics

Ethics	Information included in the manuscript?	In which section is the information available? (Reagents and Tools Table, Materials and Methods, Figures, Data Availability Section)
Studies involving **human participants**: State details of **authority granting ethics approval** (IRB or equivalent committee(s), provide reference number for approval.	Not Applicable	
Studies involving **human participants**: Include a statement confirming that **informed consent** was obtained from all subjects and that the experiments conformed to the principles set out in the WMA Declaration of Helsinki and the Department of Health and Human Services Belmont Report.	Not Applicable	
Studies involving **human participants:** For publication of **patient photos**, include a statement confirming that consent to publish was obtained.	Not Applicable	
Studies involving experimental **animals**: State details of **authority granting ethics approval** (IRB or equivalent committee(s), provide reference number for approval. Include a statement of compliance with ethical regulations.	Not Applicable	
Studies involving **specimen and field samples:** State if relevant **permits** obtained, provide details of authority approving study; if none were required, explain why.	Not Applicable	

Dual Use Research of Concern (DURC)	Information included in the manuscript?	In which section is the information available? (Reagents and Tools Table, Materials and Methods, Figures, Data Availability Section)
Could your study fall under dual use research restrictions? Please check biosecurity documents and list of **select agents and toxins** (CDC): https://www.selectagents.gov/sat/list.htm	Not Applicable	No
If you used a select agent, is the security level of the lab appropriate and reported in the manuscript?	Not Applicable	
If a study is subject to dual use research of concern regulations, is the name of the **authority granting approval and reference number** for the regulatory approval provided in the manuscript?	Not Applicable	

Reporting

The MDAR framework recommends adoption of discipline-specific guidelines, established and endorsed through community initiatives. Journals have their own policy about requiring specific guidelines and recommendations to complement MDAR.

Adherence to community standards	Information included in the manuscript?	In which section is the information available? (Reagents and Tools Table, Materials and Methods, Figures, Data Availability Section)
State if relevant guidelines or checklists (e.g., **ICMJE, MIBBI, ARRIVE, PRISMA**) have been followed or provided.	Not Applicable	
For **tumor marker prognostic studies,** we recommend that you follow the **REMARK** reporting guidelines (see link list at top right). See author guidelines, under 'Reporting Guidelines'. Please confirm you have followed these guidelines.	Not Applicable	
For **phase II and III randomized controlled trials**, please refer to the **CONSORT** flow diagram (see link list at top right) and submit the CONSORT checklist (see link list at top right) with your submission. See author guidelines, under 'Reporting Guidelines'. Please confirm you have submitted this list.	Not Applicable	

Data Availability

Data availability	Information included in the manuscript?	In which section is the information available? (Reagents and Tools Table, Materials and Methods, Figures, Data Availability Section)
Have **primary datasets** been deposited according to the journal's guidelines (see 'Data Deposition' section) and the respective accession numbers provided in the Data Availability Section?	Yes	Data Availability Section
Were **human clinical and genomic datasets** deposited in a public access-controlled repository in accordance to ethical obligations to the patients and to the applicable consent agreement?	Not Applicable	No
Are **computational models** that are central and integral to a study available without restrictions in a machine-readable form? Were the relevant accession numbers or links provided?	Not Applicable	No
If publicly available data were reused, provide the respective **data citations in the reference list**.	Yes	Figure legends
