# Supplementary material for: AURORA A interacts with DICER and SETD2 to promote S-phase progression
Source: EMBO Rep. 2026 Jun 30;27(15):4493–523. doi: 10.1038/s44319-026-00850-0 (PMC13458143; doi:10.1038/s44319-026-00850-0)
Supplement: Supplementary file 3 [file 44319_2026_850_MOESM10_ESM.zip › Figure 4/4D/4D_PB222_4_R4_Seq003moreImages_CellCyclePlot_PLApositive_Thr50_Control.pdf]

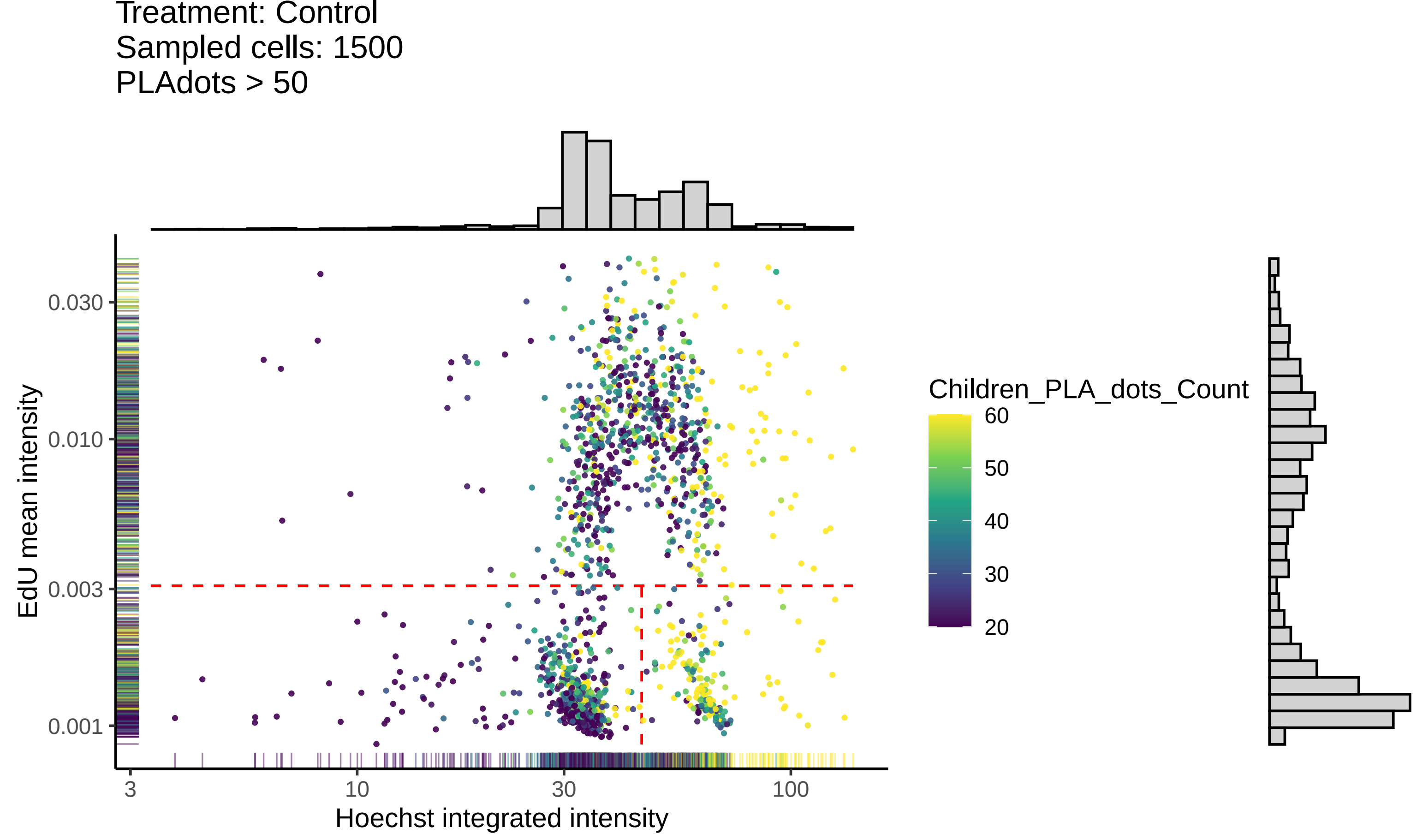
Treatment: Control
Sampled cells: 1500
PLAdots > 50
EdU mean intensity
0.030
0.010
0.003
0.001
3
10
30
100
Hoechst integrated intensity
Children_PLA_dots_Count
60
50
40
30
20

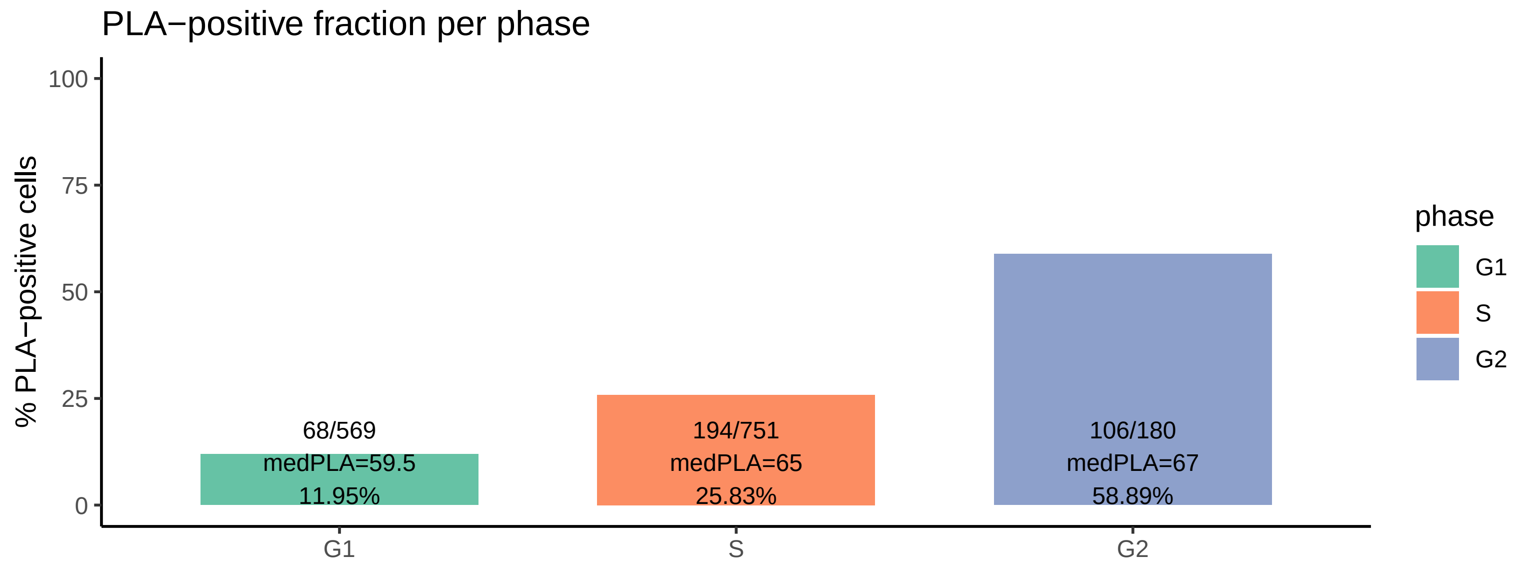
PLA−positive fraction per phase
% PLA−positive cells
100
75
50
25
0
68/569
medPLA=59.5
11.95%
194/751
medPLA=65
25.83%
106/180
medPLA=67
58.89%
G1
S
G2
phase
G1
S
G2
