# Supplementary material for: AURORA A interacts with DICER and SETD2 to promote S-phase progression
Source: EMBO Rep. 2026 Jun 30;27(15):4493–523. doi: 10.1038/s44319-026-00850-0 (PMC13458143; doi:10.1038/s44319-026-00850-0)
Supplement: Supplementary file 4 [file 44319_2026_850_MOESM11_ESM.zip › Figure 5/5A/5A_WB_overview.pdf]

5A

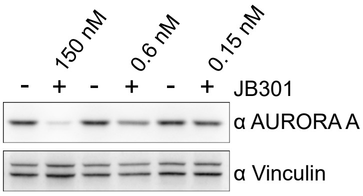
150 nM
0.6 nM
0.15 nM
- + - + - + JB301
α AURORA A
α Vinculin

Raw data:

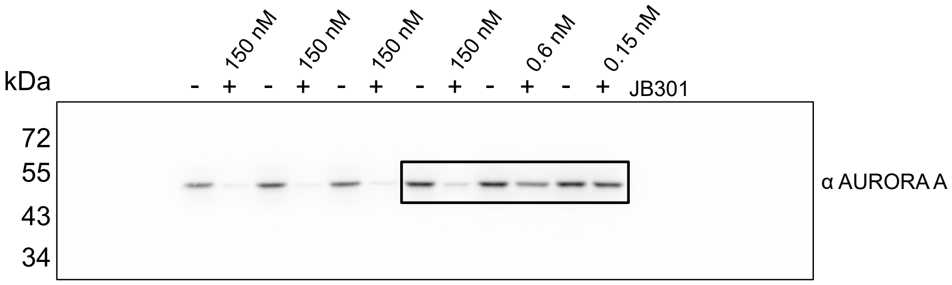
150 nM
150 nM
150 nM
150 nM
0.6 nM
0.15 nM
kDa
- + - + - + - + - + - + JB301
72
55
43
34
α AURORA A

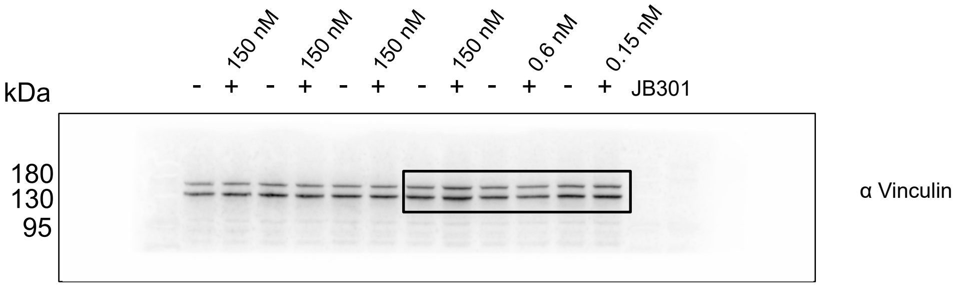
150 nM
150 nM
150 nM
150 nM
0.6 nM
0.15 nM
kDa
- + - + - + - + - + - + JB301
180
130
95
α Vinculin
