# Supplementary material for: AURORA A interacts with DICER and SETD2 to promote S-phase progression
Source: EMBO Rep. 2026 Jun 30;27(15):4493–523. doi: 10.1038/s44319-026-00850-0 (PMC13458143; doi:10.1038/s44319-026-00850-0)
Supplement: Supplementary file 4 [file 44319_2026_850_MOESM11_ESM.zip › Figure 5/5G/Volcano_SETD2_gene_body_coverage_3_vs_2.pdf]

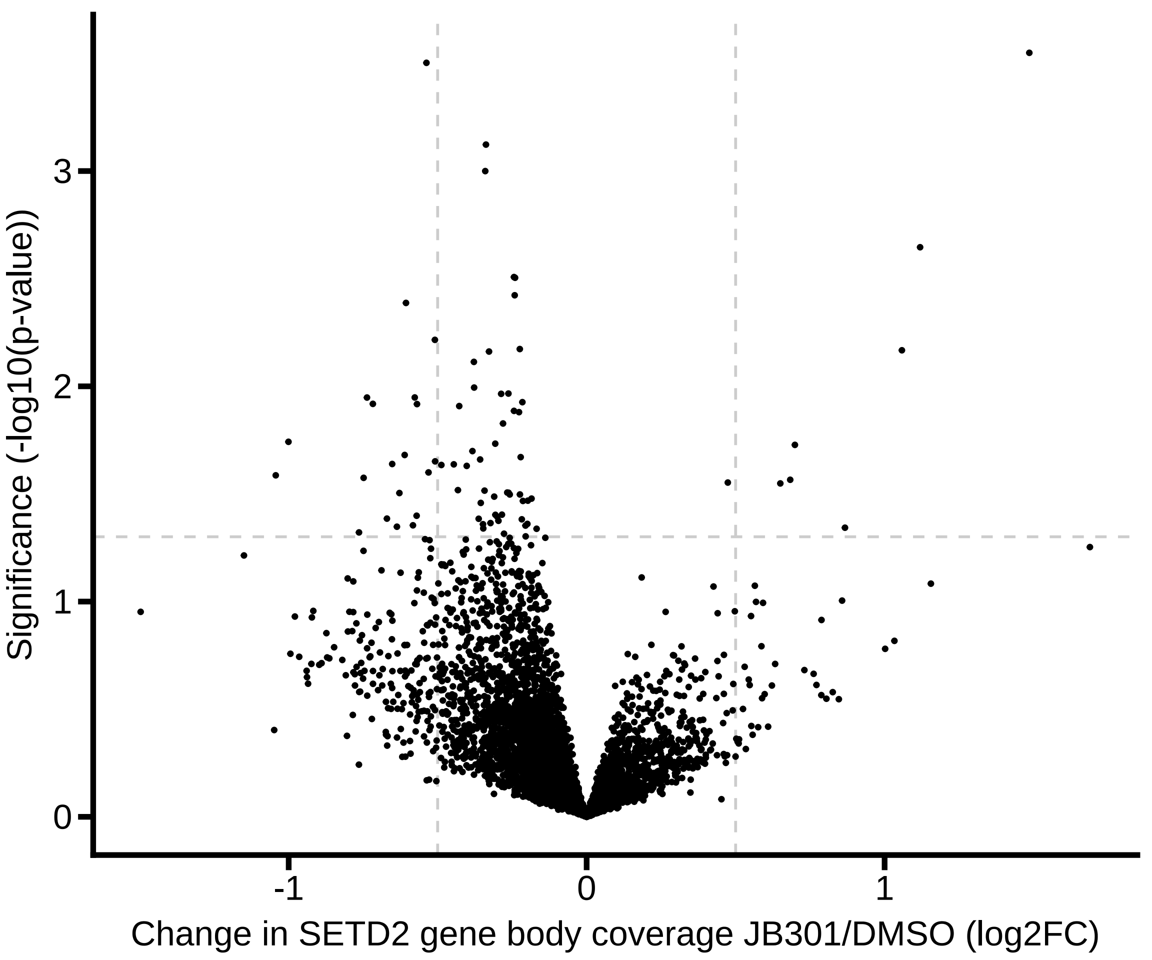

Significance (-log10(p-value))
3
2
1
0
-1
0
1
Change in SETD2 gene body coverage JB301/DMSO (log2FC)
