# Supplementary material for: AURORA A interacts with DICER and SETD2 to promote S-phase progression
Source: EMBO Rep. 2026 Jun 30;27(15):4493–523. doi: 10.1038/s44319-026-00850-0 (PMC13458143; doi:10.1038/s44319-026-00850-0)
Supplement: Supplementary file 4 [file 44319_2026_850_MOESM11_ESM.zip › Figure 5/5H/SETD2_metagene_all_replicates_expressed_R_loops_level_4.pdf]

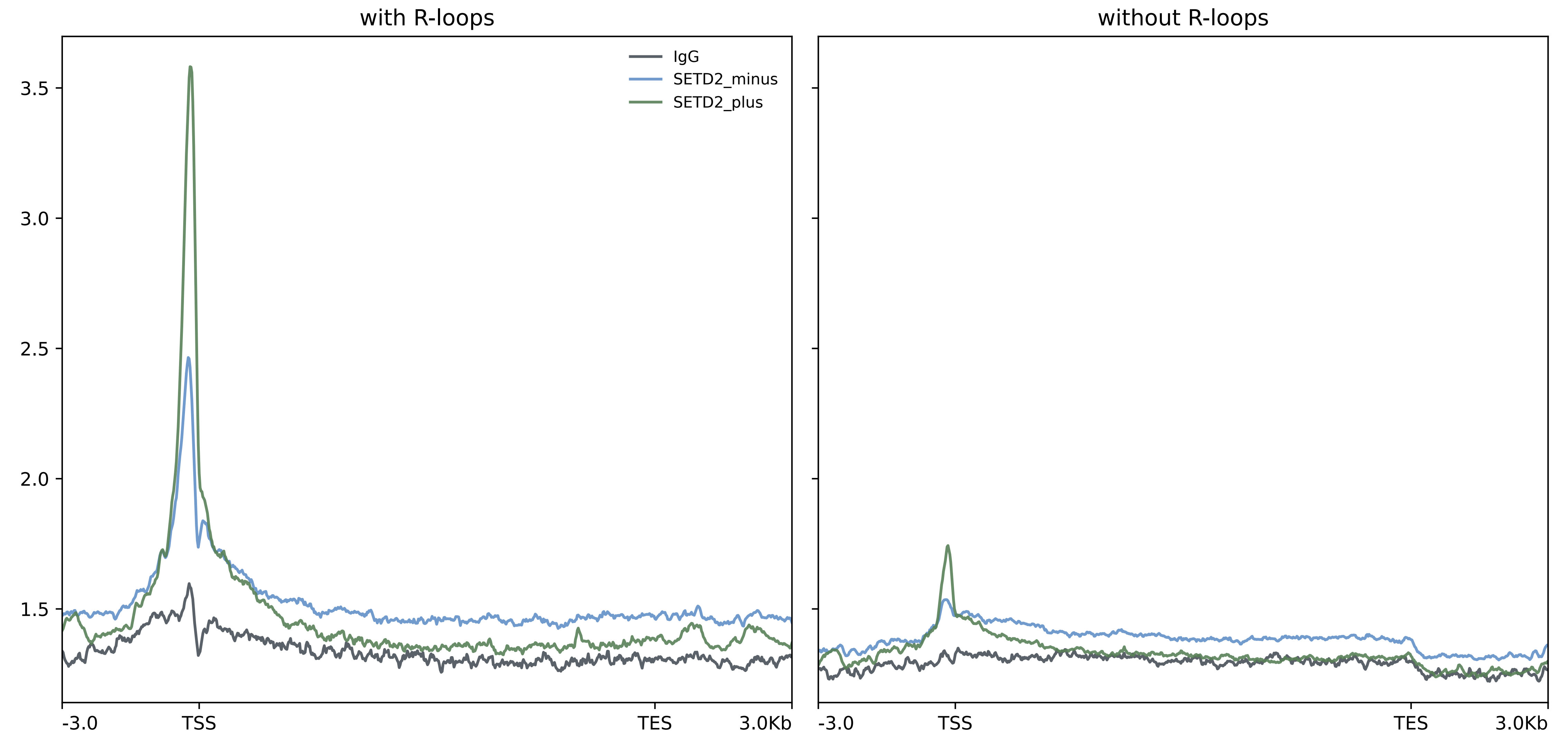

with R-loops
without R-loops
IgG
SETD2_minus
SETD2_plus
3.5
3.0
2.5
2.0
1.5
-3.0
TSS
TES
3.0Kb
-3.0
TSS
TES
3.0Kb
