# Supplementary material for: AURORA A interacts with DICER and SETD2 to promote S-phase progression
Source: EMBO Rep. 2026 Jun 30;27(15):4493–523. doi: 10.1038/s44319-026-00850-0 (PMC13458143; doi:10.1038/s44319-026-00850-0)
Supplement: Supplementary file 4 [file 44319_2026_850_MOESM11_ESM.zip › Figure 5/5I/SETD2_promoter_ratio_change_R_loops_4.pdf]

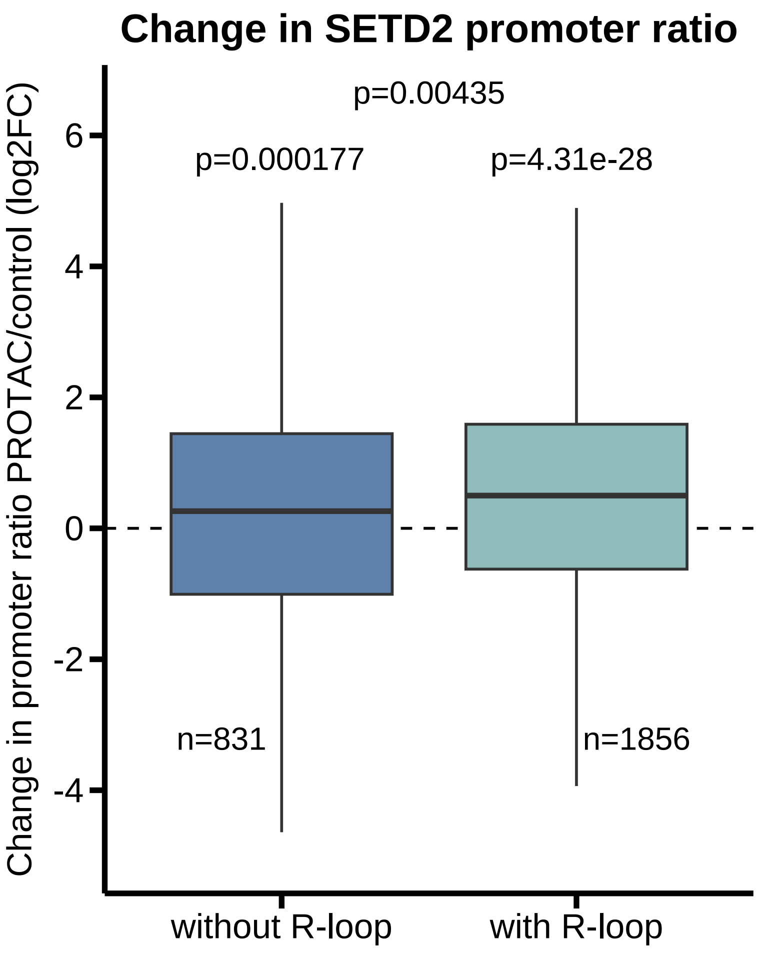

Change in SETD2 promoter ratio
p=0.00435
p=0.000177
p=4.31e-28
n=831
n=1856
Change in promoter ratio PROTAC/control (log2FC)
6
4
2
0
-2
-4
without R-loop
with R-loop
