# Supplementary material for: AURORA A interacts with DICER and SETD2 to promote S-phase progression
Source: EMBO Rep. 2026 Jun 30;27(15):4493–523. doi: 10.1038/s44319-026-00850-0 (PMC13458143; doi:10.1038/s44319-026-00850-0)
Supplement: Supplementary file 4 [file 44319_2026_850_MOESM11_ESM.zip › Figure 5/5J/SETD2_gene_body_coverage_change_DEseq_R_loops_4.pdf]

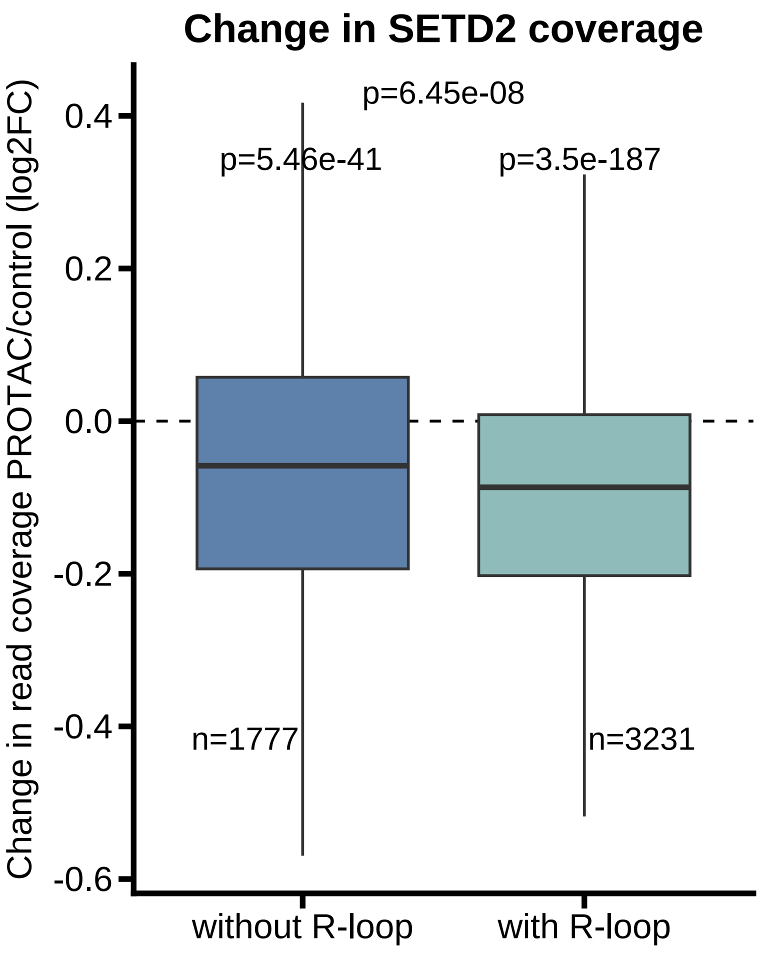

Change in SETD2 coverage
Change in read coverage PROTAC/control (log2FC)
0.4
0.2
0.0
-0.2
-0.4
-0.6
p=6.45e-08
p=5.46e-41
p=3.5e-187
n=1777
n=3231
without R-loop
with R-loop
