# Supplementary material for: AURORA A interacts with DICER and SETD2 to promote S-phase progression
Source: EMBO Rep. 2026 Jun 30;27(15):4493–523. doi: 10.1038/s44319-026-00850-0 (PMC13458143; doi:10.1038/s44319-026-00850-0)
Supplement: Supplementary file 5 [file 44319_2026_850_MOESM7_ESM.zip › Figure 1/1D/Gating_strategy.pdf]

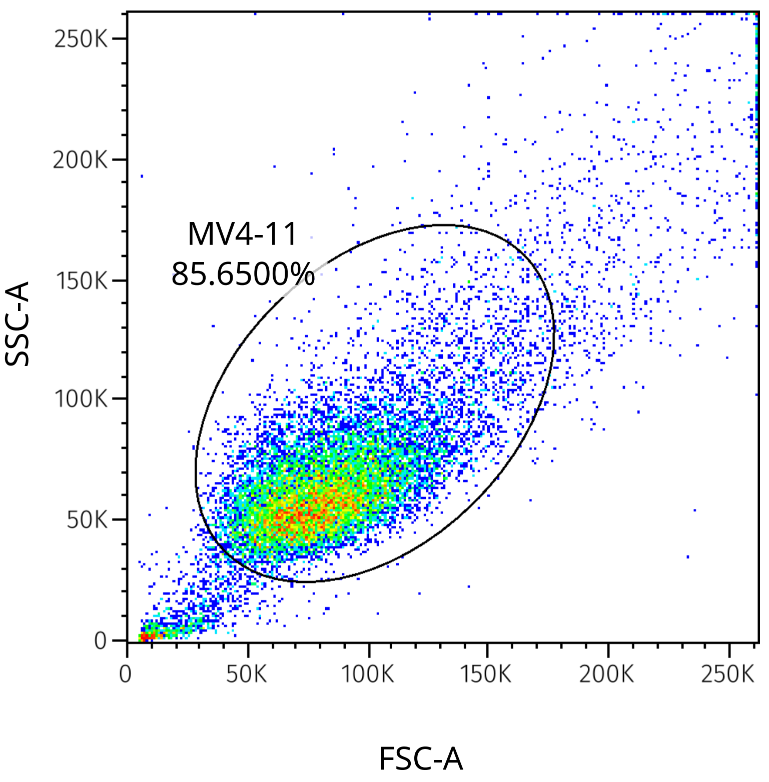

Sample Name: R1_DMSO_P1437.fcs
Population Name: Ungated
Count: 16000

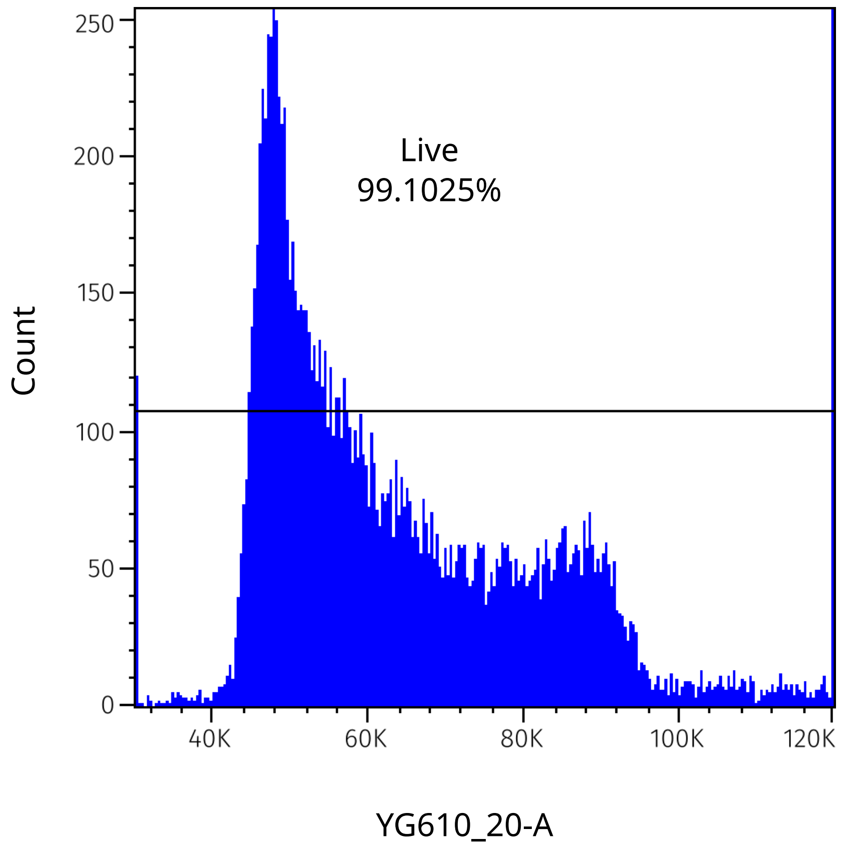

Sample Name: R1_DMSO_P1437.fcs
Population Name: MV4-11
Count: 13704

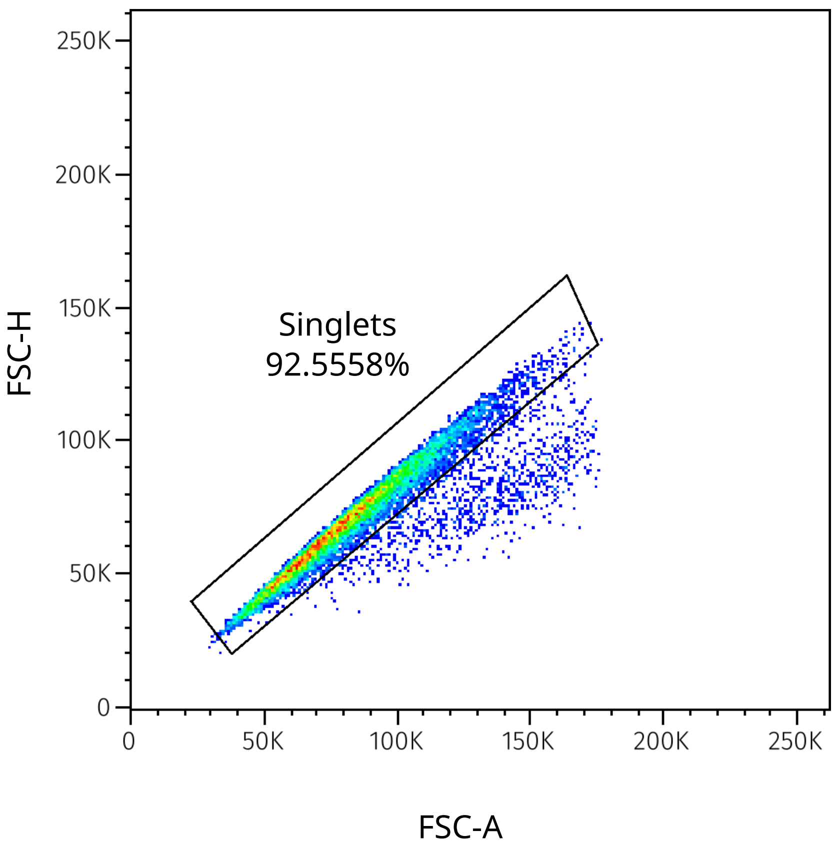

Sample Name: R1_DMSO_P1437.fcs
Population Name: Live
Count: 13581

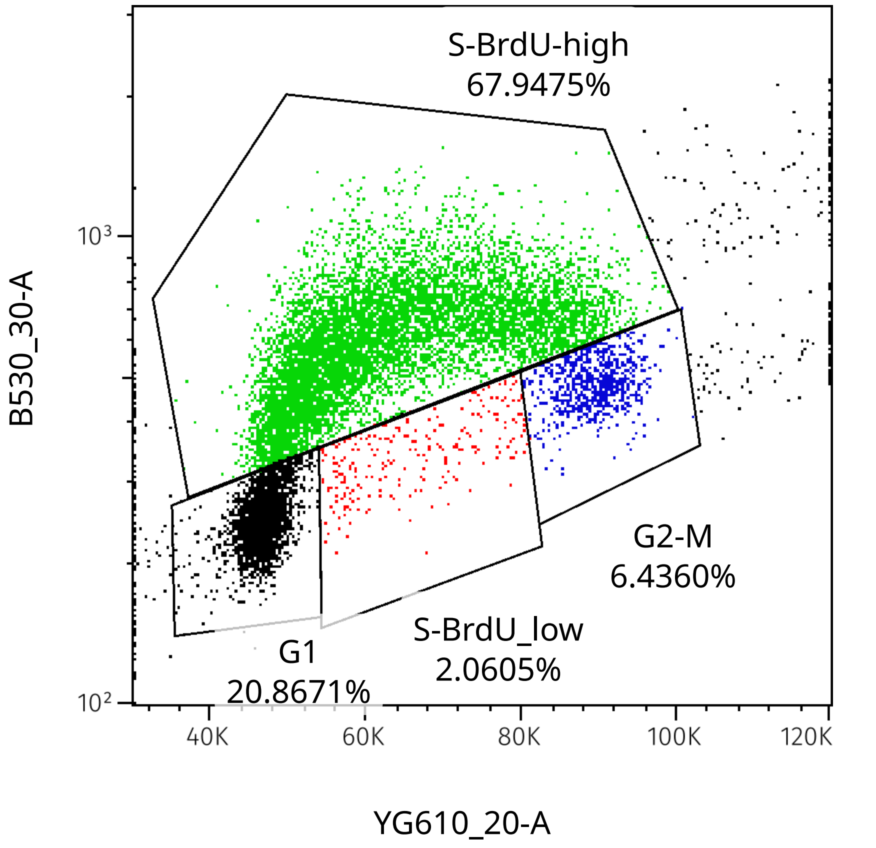

	Sample Name	Population Name	Count
	R1_DMSO_P1437.fcs	G1	2623
	R1_DMSO_P1437.fcs	S-BrdU-high	8541
	R1_DMSO_P1437.fcs	S-BrdU_low	259
	R1_DMSO_P1437.fcs	G2-M	809
	R1_DMSO_P1437.fcs	Singlets	12570
