# Supplementary material for: AURORA A interacts with DICER and SETD2 to promote S-phase progression
Source: EMBO Rep. 2026 Jun 30;27(15):4493–523. doi: 10.1038/s44319-026-00850-0 (PMC13458143; doi:10.1038/s44319-026-00850-0)
Supplement: Supplementary file 5 [file 44319_2026_850_MOESM7_ESM.zip › Figure 1/1D/3_Report/R1.pdf]

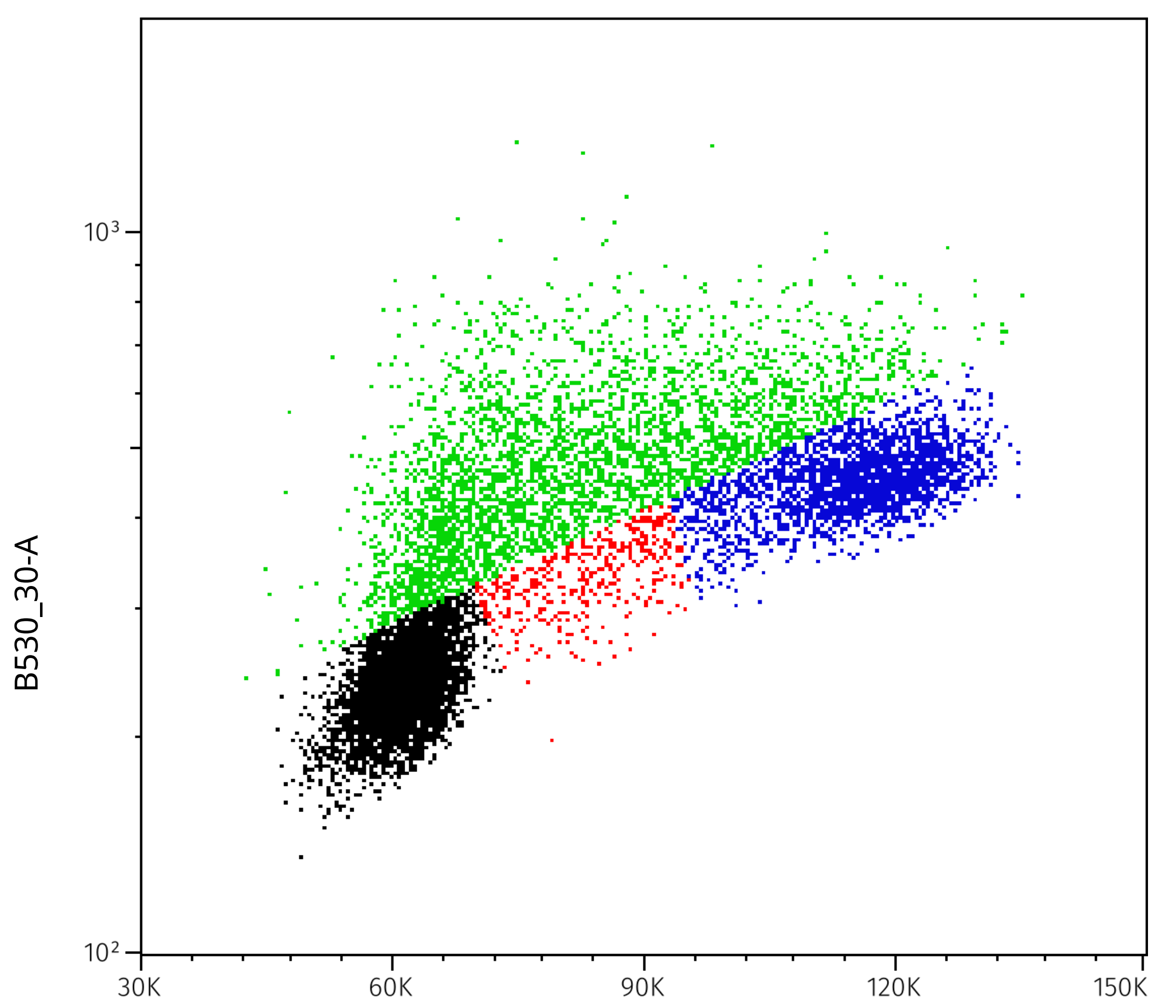

	Sample Name	Population Name	Count	Ungated/MV4-11/Live/Singlets/S-BrdU_high Freq. of Parent
■ (black)	DMSO.fcs	G1	4597	40.9897%
■ (green)	DMSO.fcs	S-BrdU_high	3630	32.3674%
■ (red)	DMSO.fcs	S-BrdU_low	425	3.7896%
■ (blue)	DMSO.fcs	G2-M	2422	21.5961%

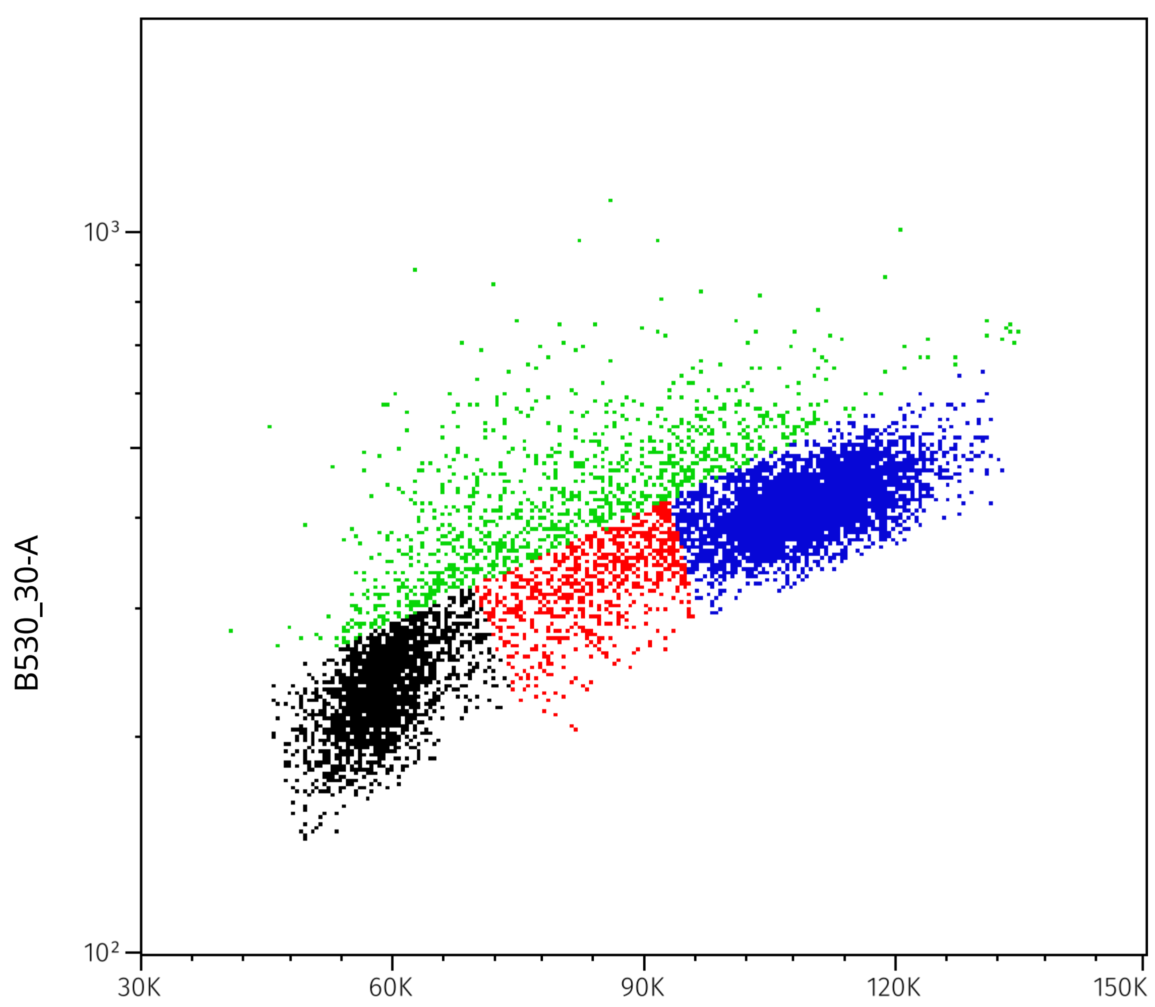

	Sample Name	Population Name	Count	Ungated/MV4-11/Live/Singlets/S-BrdU_high Freq. of Parent
■	JB170.fcs	G1	2465	26.9900%
■	JB170.fcs	S-BrdU_high	1194	13.0735%
■	JB170.fcs	S-BrdU_low	843	9.2303%
■	JB170.fcs	G2-M	4478	49.0310%

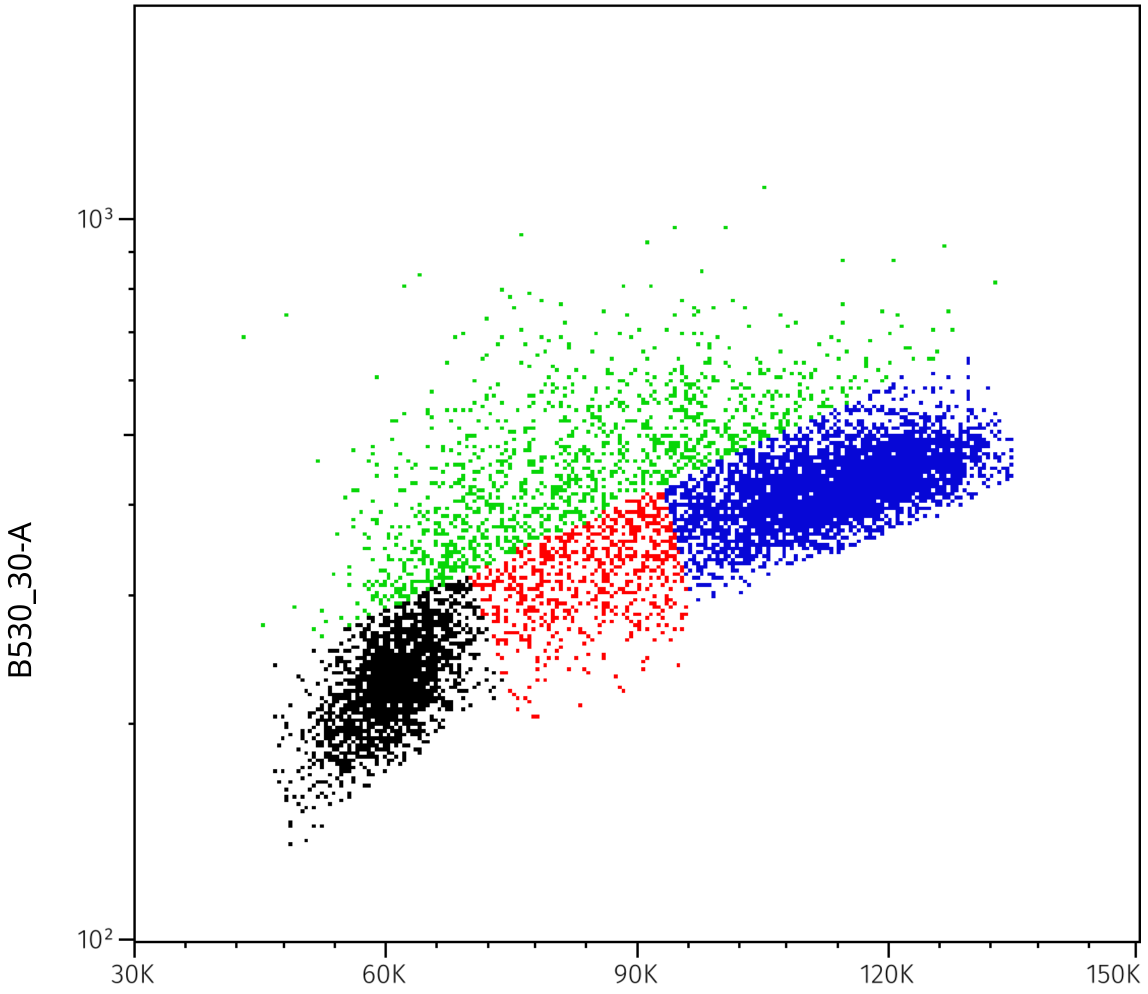

	Sample Name	Population Name	Count	Ungated/MV4-11/Live/Singlets/S-BrdU_high Freq. of Parent
(black)	JB301.fcs	G1	2347	24.7757%
(green)	JB301.fcs	S-BrdU_high	1335	14.0927%
(red)	JB301.fcs	S-BrdU_low	732	7.7272%
(blue)	JB301.fcs	G2-M	4852	51.2193%

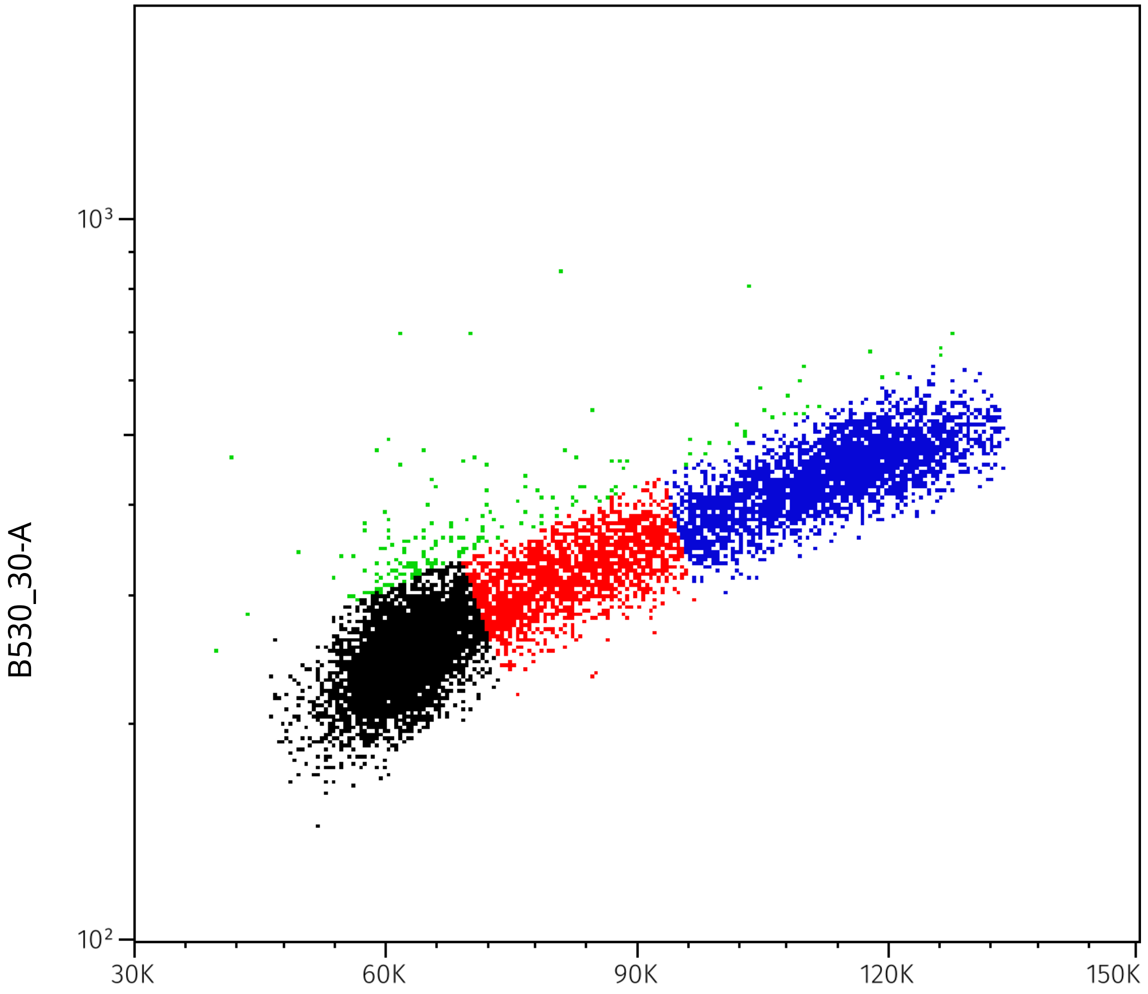

	Sample Name	Population Name	Count	Ungated/MV4-11/Live/Singlets/S-BrdU_high Freq. of Parent
■ (black)	no-BrdU_DMSO.fcs	G1	5040	47.4710%
■ (green)	no-BrdU_DMSO.fcs	S-BrdU_high	198	1.8649%
■ (red)	no-BrdU_DMSO.fcs	S-BrdU_low	1948	18.3479%
■ (blue)	no-BrdU_DMSO.fcs	G2-M	3308	31.1576%

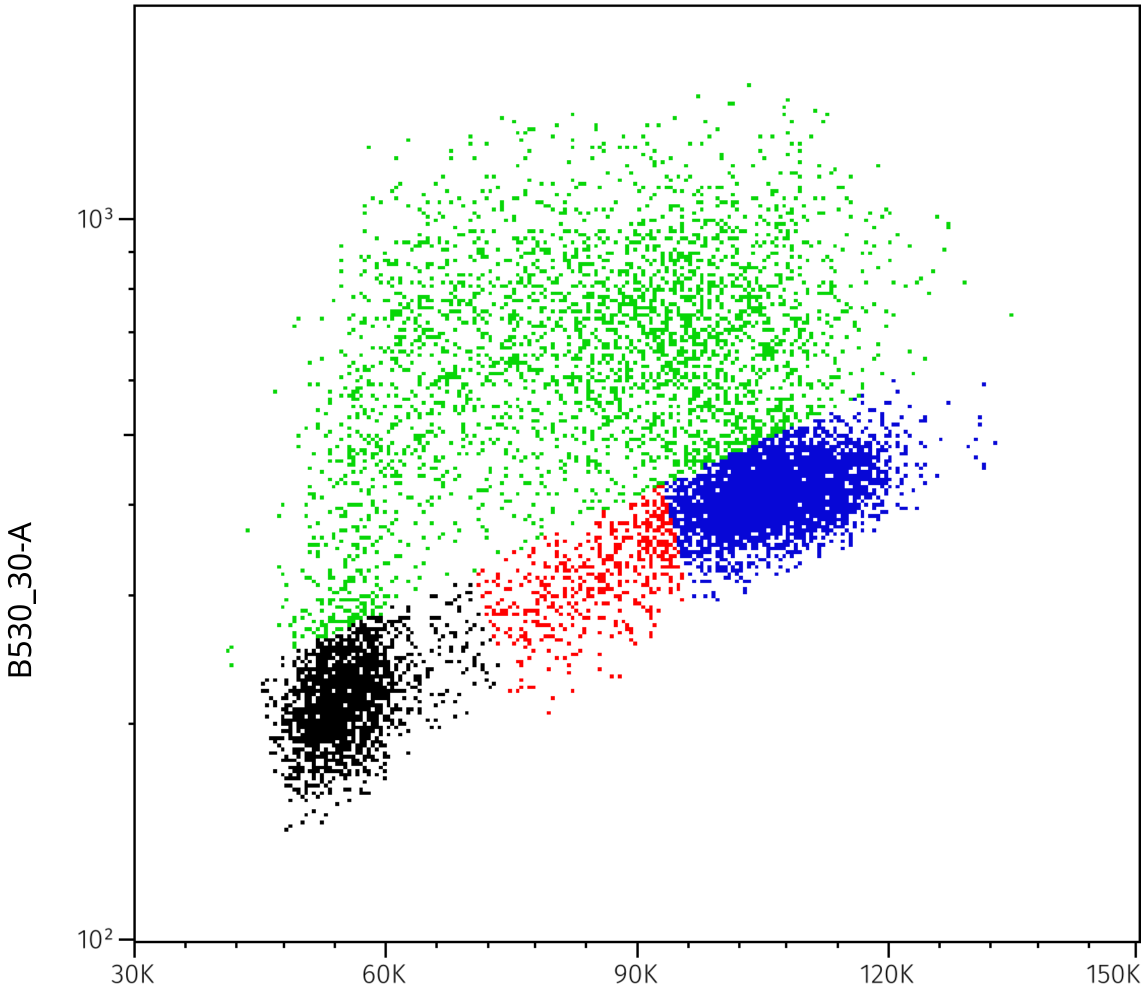

	Sample Name	Population Name	Count	Ungated/MV4-11/Live/Singlets/S-BrdU_high Freq. of Parent
■ (black)	Alisertib.fcs	G1	1902	19.4578%
■ (green)	Alisertib.fcs	S-BrdU_high	3152	32.2455%
■ (red)	Alisertib.fcs	S-BrdU_low	527	5.3913%
■ (blue)	Alisertib.fcs	G2-M	3991	40.8286%
