# Supplementary material for: AURORA A interacts with DICER and SETD2 to promote S-phase progression
Source: EMBO Rep. 2026 Jun 30;27(15):4493–523. doi: 10.1038/s44319-026-00850-0 (PMC13458143; doi:10.1038/s44319-026-00850-0)
Supplement: Supplementary file 5 [file 44319_2026_850_MOESM7_ESM.zip › Figure 1/1D/3_Report/R2-R3.pdf]

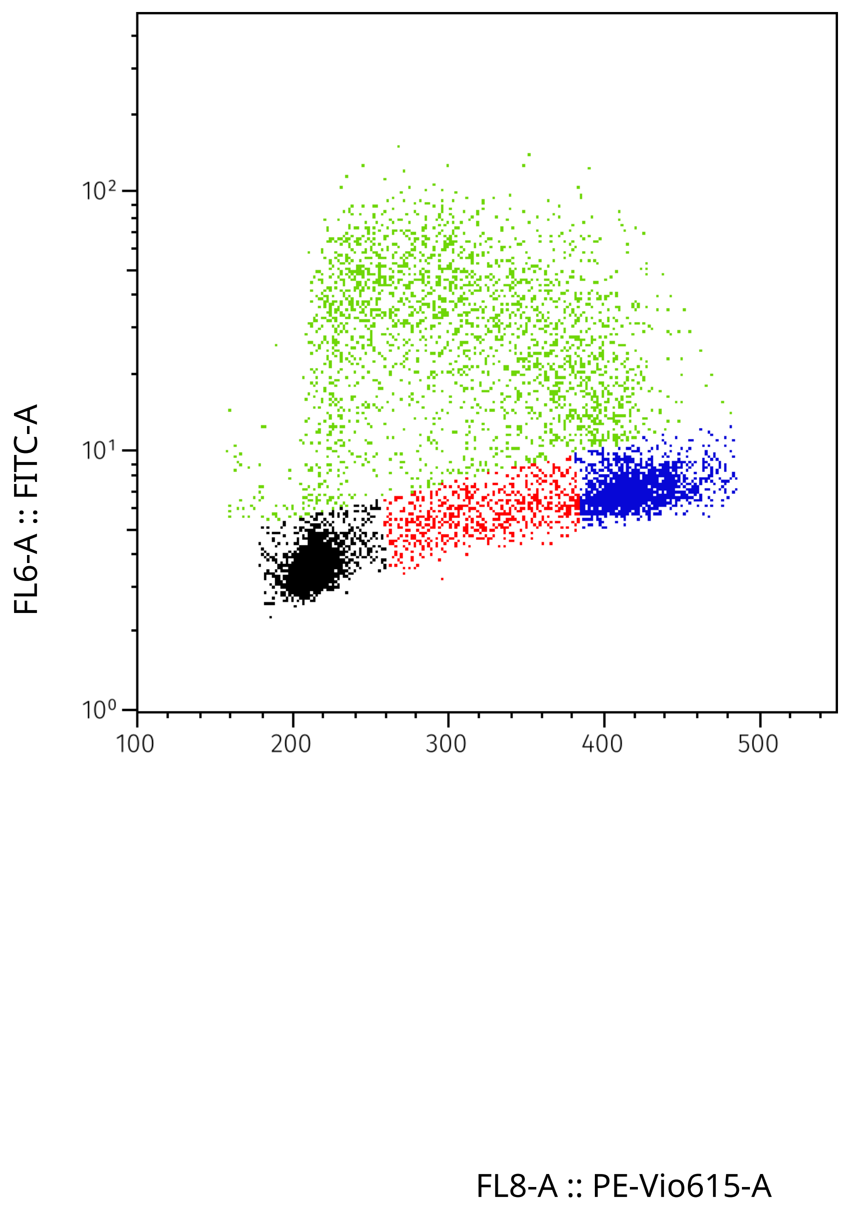

	Sample Name	Population Name	Count	Ungated Freq. of Parent
■ (black)	BA2026-03-10_DMSO_R1.0001.fcs	G1	2439	30.1260%
■ (green)	BA2026-03-10_DMSO_R1.0001.fcs	S-BrdU_high	2575	31.8058%
■ (red)	BA2026-03-10_DMSO_R1.0001.fcs	S-BrdU-low	670	8.2757%
■ (blue)	BA2026-03-10_DMSO_R1.0001.fcs	G2-M	1906	23.5425%

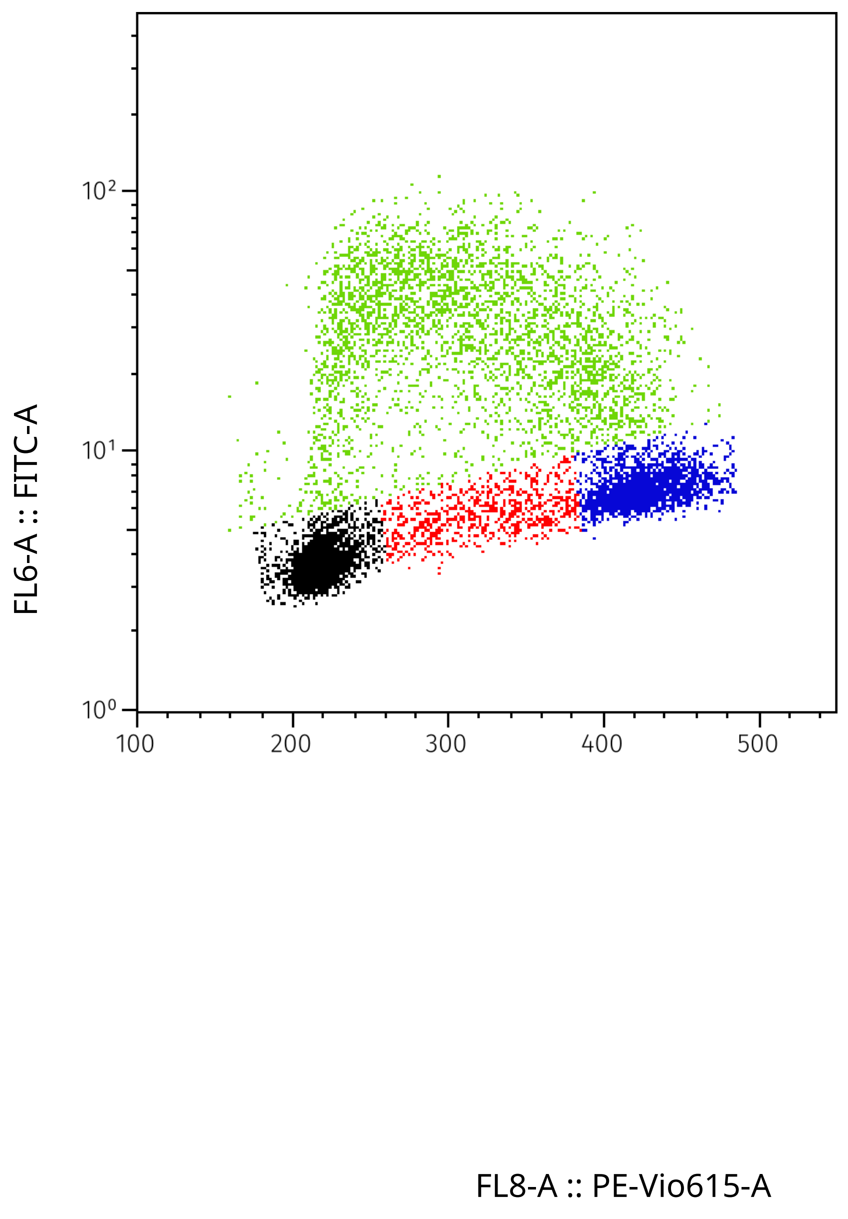

	Sample Name	Population Name	Count	Ungated Freq. of Parent
■ (black)	BA2026-03-10_DMSO_R2.0001.fcs	G1	3493	32.3276%
■ (green)	BA2026-03-10_DMSO_R2.0001.fcs	S-BrdU_high	3560	32.9477%
■ (red)	BA2026-03-10_DMSO_R2.0001.fcs	S-BrdU-low	778	7.2004%
■ (blue)	BA2026-03-10_DMSO_R2.0001.fcs	G2-M	2374	21.9713%

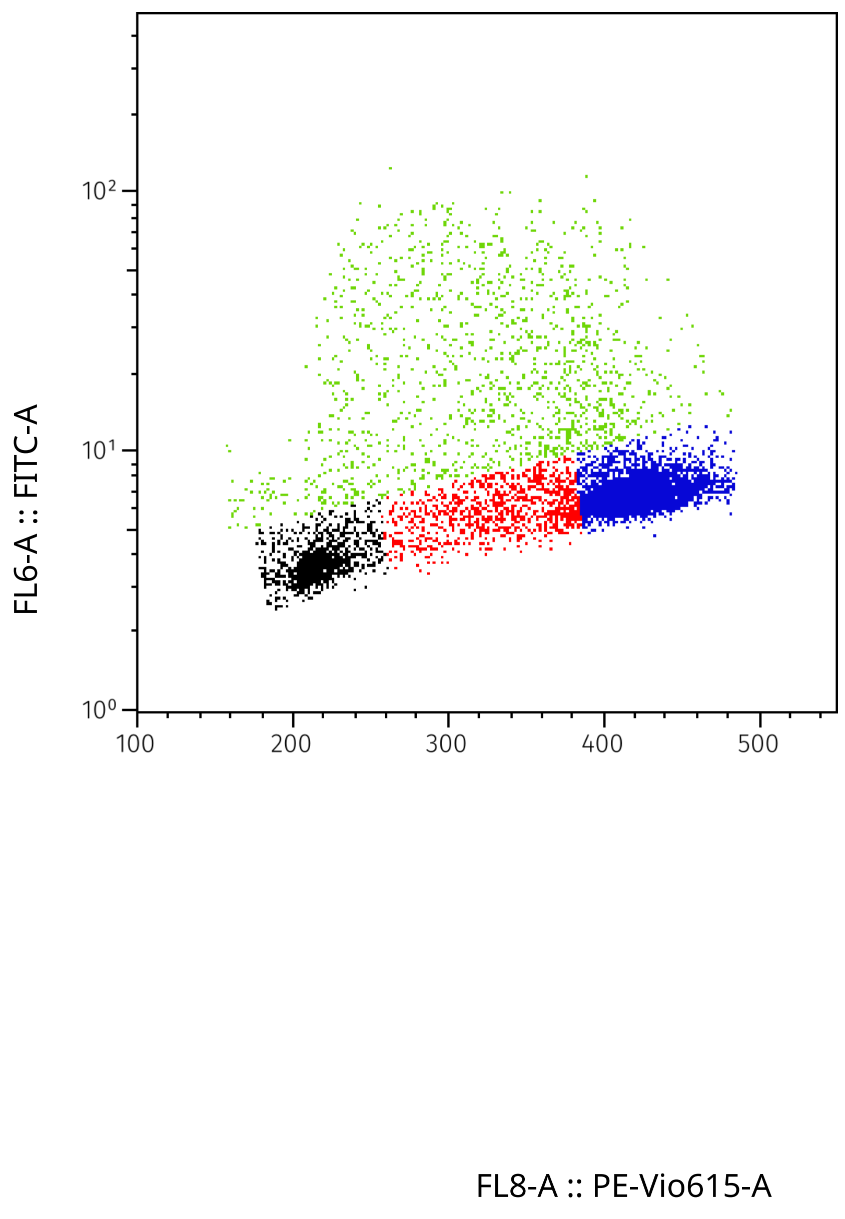

	Sample Name	Population Name	Count	Ungated Freq. of Parent
■ (black)	BA2026-03-10_JB170_R1.0001.fcs	G1	1284	15.7026%
■ (green)	BA2026-03-10_JB170_R1.0001.fcs	S-BrdU_high	1406	17.1946%
■ (red)	BA2026-03-10_JB170_R1.0001.fcs	S-BrdU-low	1015	12.4129%
■ (blue)	BA2026-03-10_JB170_R1.0001.fcs	G2-M	3999	48.9055%

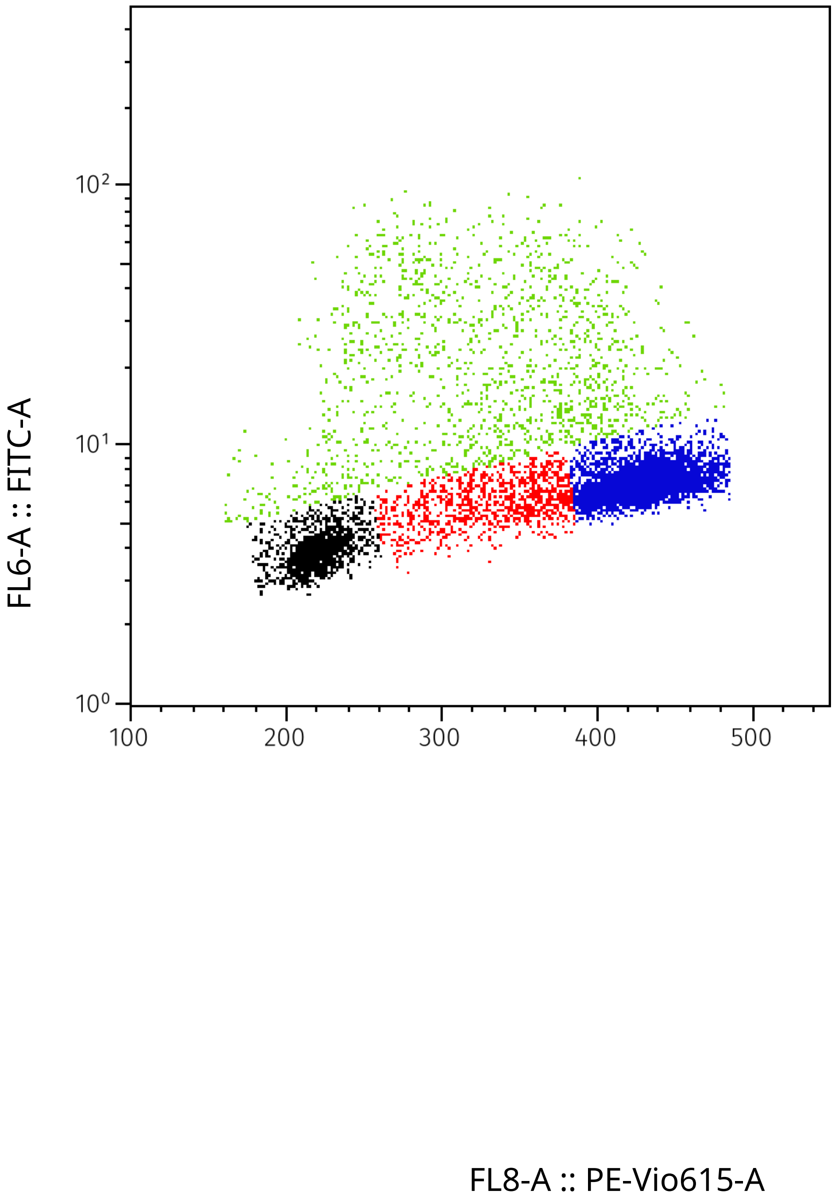

	Sample Name	Population Name	Count	Ungated Freq. of Parent
■ (black)	BA2026-03-10_JB170_R2.0001.fcs	G1	1340	15.1824%
■ (green)	BA2026-03-10_JB170_R2.0001.fcs	S-BrdU_high	1464	16.5874%
■ (red)	BA2026-03-10_JB170_R2.0001.fcs	S-BrdU-low	1000	11.3302%
■ (blue)	BA2026-03-10_JB170_R2.0001.fcs	G2-M	4535	51.3823%

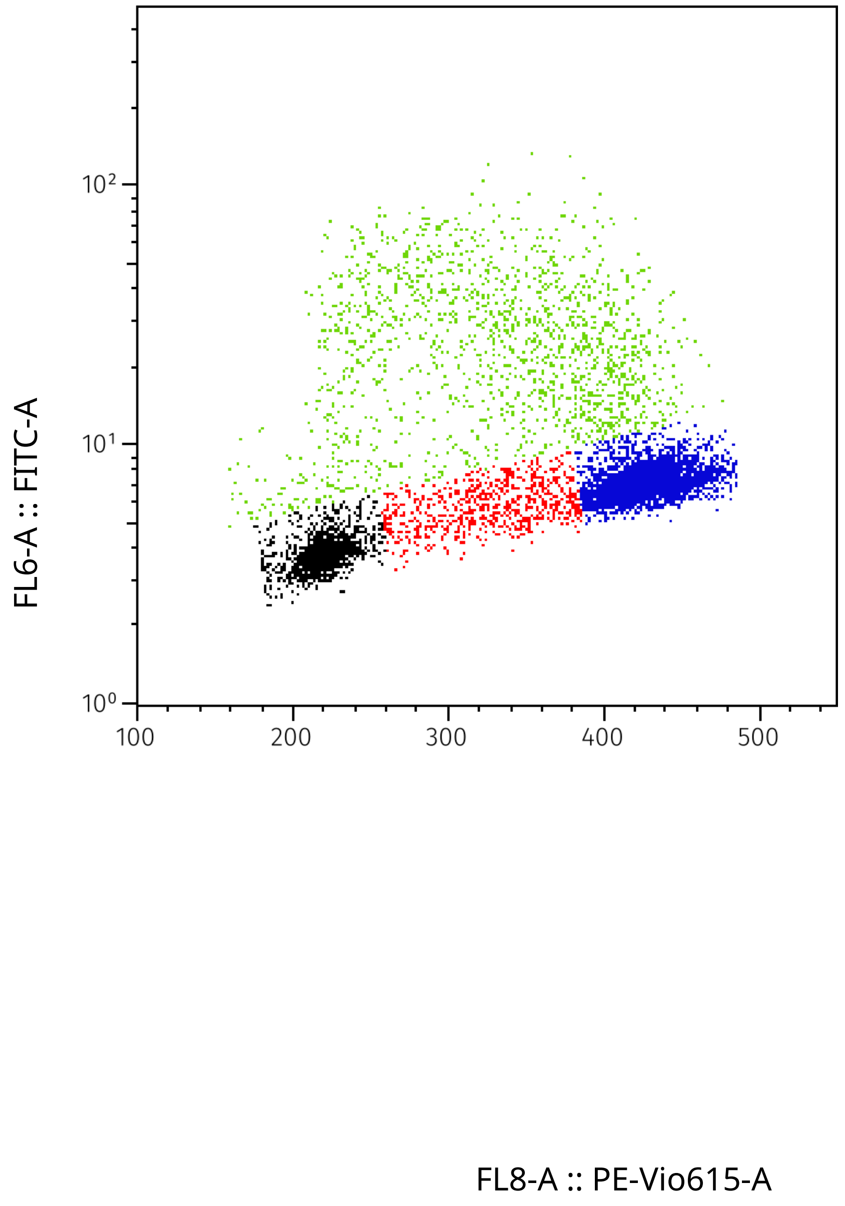

	Sample Name	Population Name	Count	Ungated Freq. of Parent
■ (black)	BA2026-03-10_JB301_R1.0001.fcs	G1	1319	17.5703%
■ (green)	BA2026-03-10_JB301_R1.0001.fcs	S-BrdU_high	1643	21.8862%
■ (red)	BA2026-03-10_JB301_R1.0001.fcs	S-BrdU-low	725	9.6577%
■ (blue)	BA2026-03-10_JB301_R1.0001.fcs	G2-M	3385	45.0912%

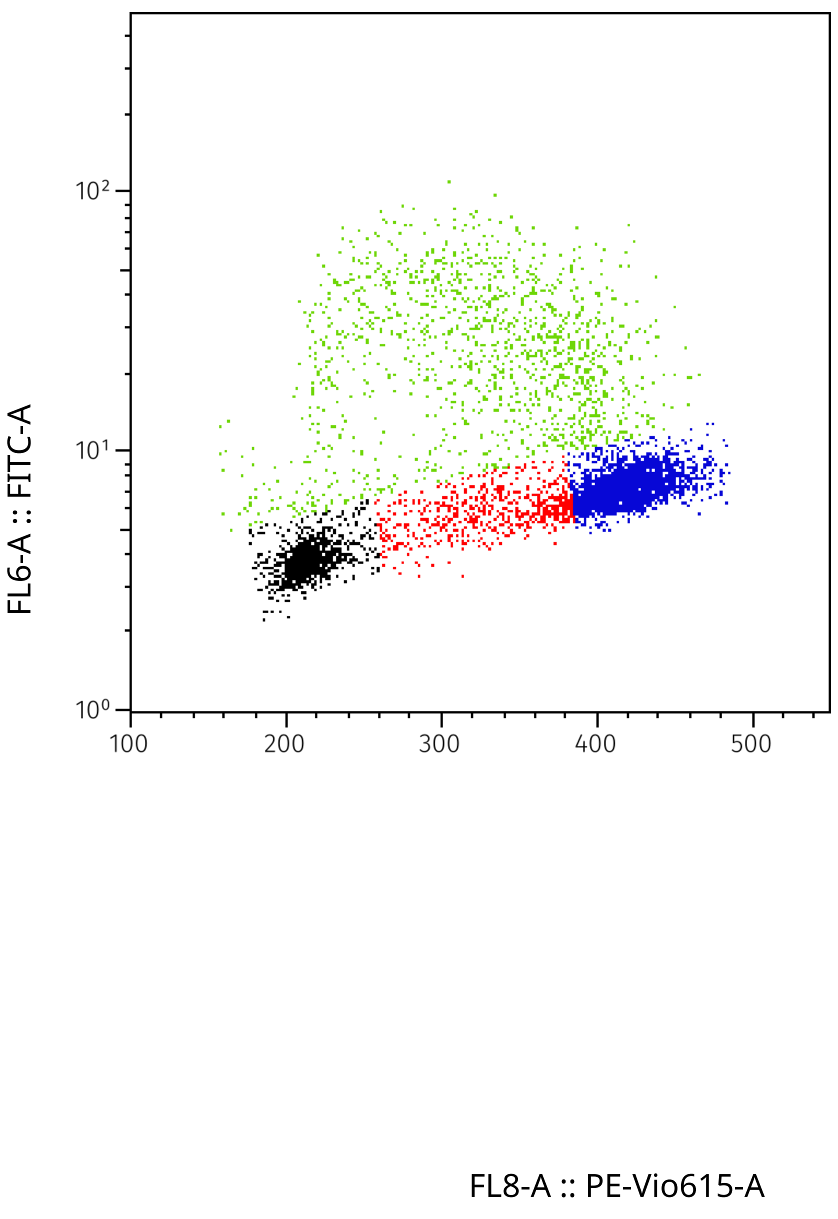

	Sample Name	Population Name	Count	Ungated Freq. of Parent
	BA2026-03-10_JB301_R2.0001.fcs	G1	1143	18.3673%
	BA2026-03-10_JB301_R2.0001.fcs	S-BrdU_high	1389	22.3204%
	BA2026-03-10_JB301_R2.0001.fcs	S-BrdU-low	688	11.0558%
	BA2026-03-10_JB301_R2.0001.fcs	G2-M	2654	42.6482%

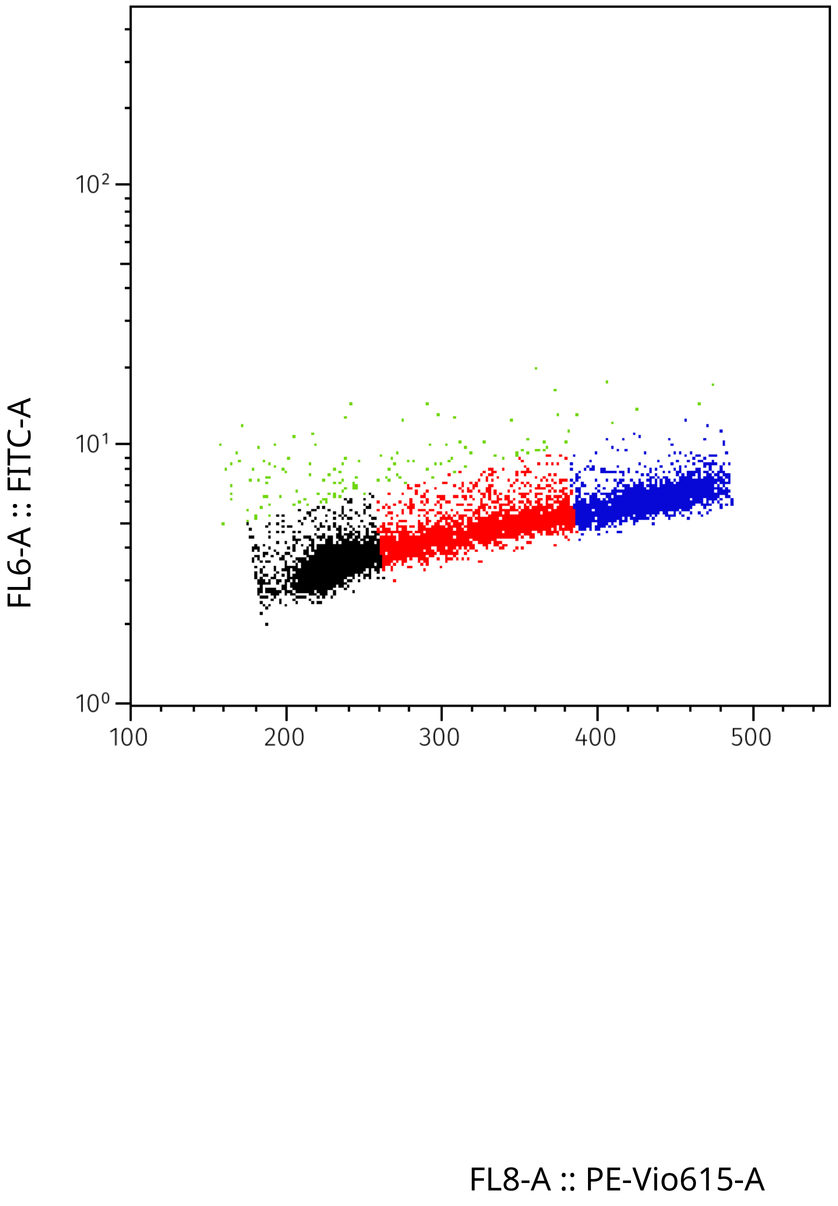

	Sample Name	Population Name	Count	Ungated Freq. of Parent
■	BA2026-03-10_no BrdU.0002.fcs	G1	5031	42.7152%
■	BA2026-03-10_no BrdU.0002.fcs	S-BrdU_high	133	1.1292%
■	BA2026-03-10_no BrdU.0002.fcs	S-BrdU-low	2773	23.5439%
■	BA2026-03-10_no BrdU.0002.fcs	G2-M	3305	28.0608%

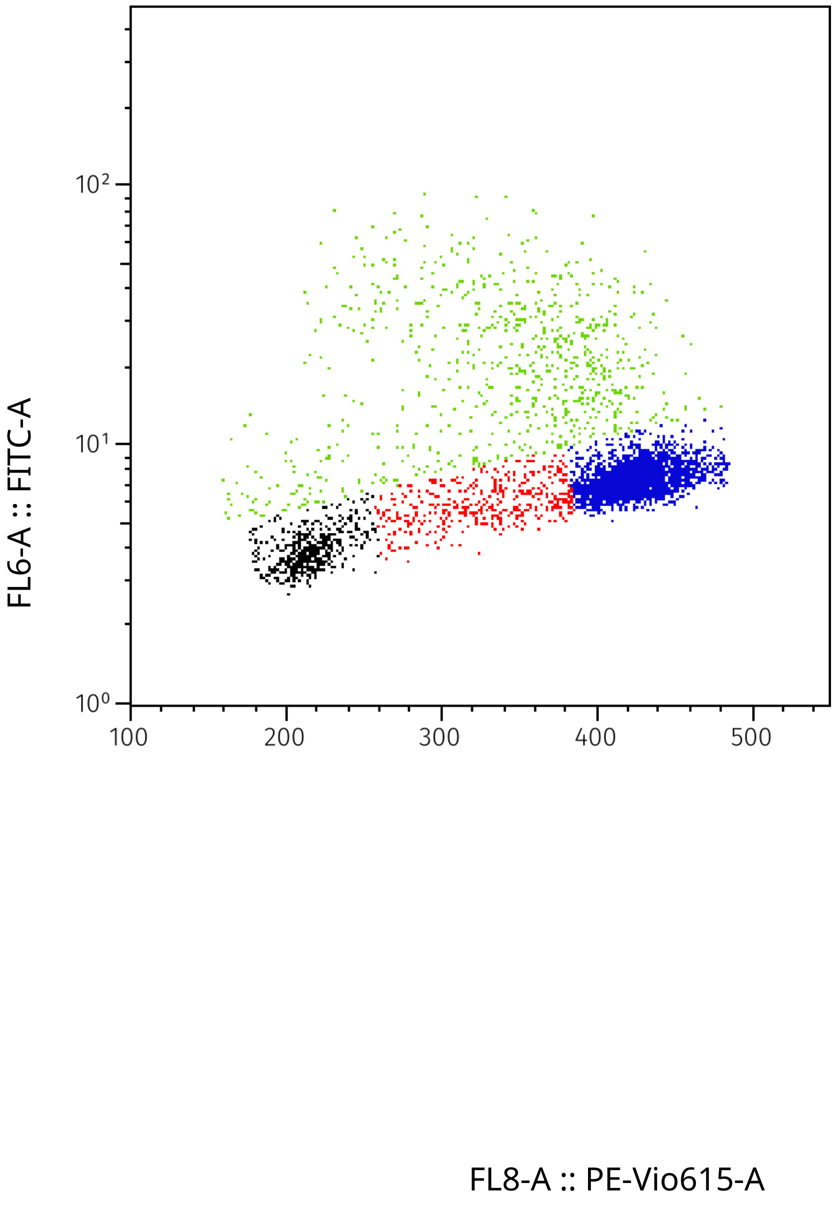

	Sample Name	Population Name	Count	Ungated Freq. of Parent
■ (black)	BA2026-03-10_Alisertib_R1.0001.fcs	G1	478	9.8801%
■ (green)	BA2026-03-10_Alisertib_R1.0001.fcs	S-BrdU_high	789	16.3084%
■ (red)	BA2026-03-10_Alisertib_R1.0001.fcs	S-BrdU-low	429	8.8673%
■ (blue)	BA2026-03-10_Alisertib_R1.0001.fcs	G2-M	2845	58.8053%

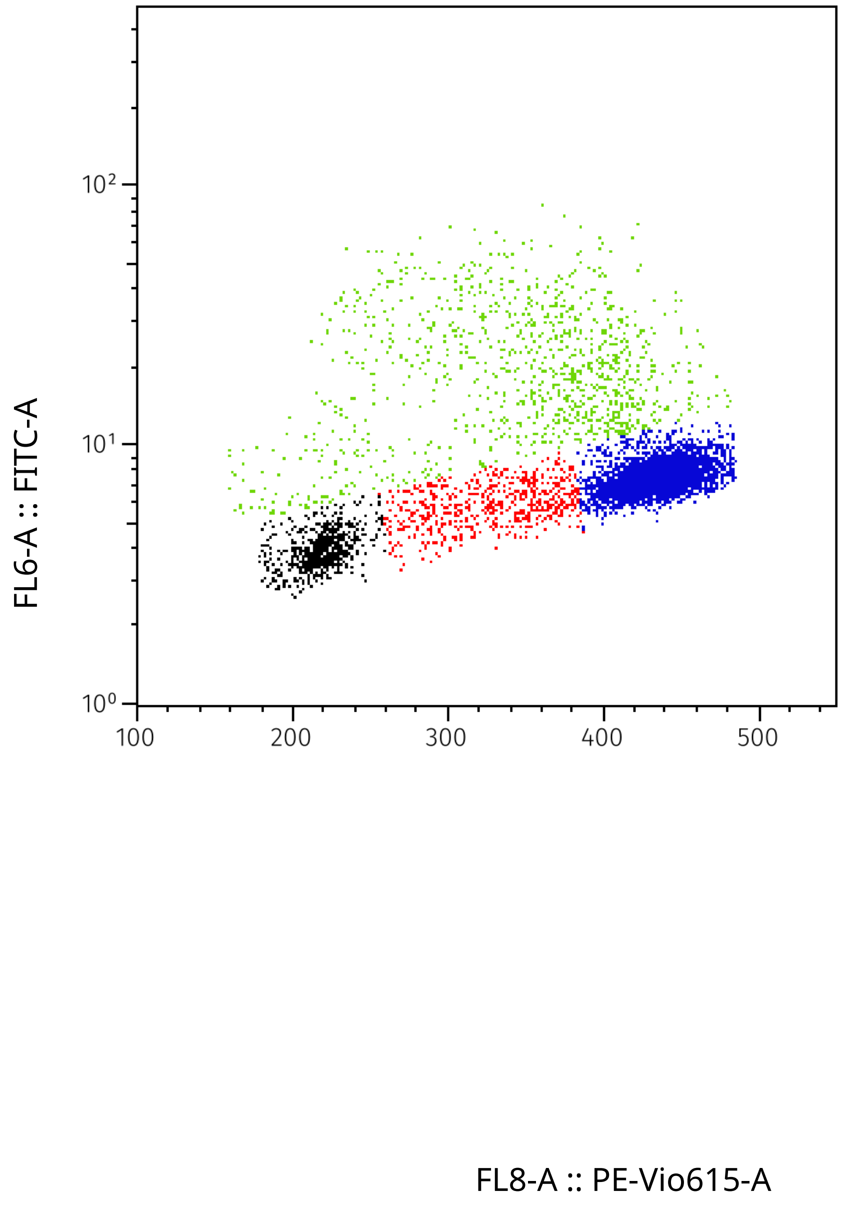

	Sample Name	Population Name	Count	Ungated Freq. of Parent
■ (black)	BA2026-03-10_Alisertib_R2.0001.fcs	G1	647	9.5838%
■ (green)	BA2026-03-10_Alisertib_R2.0001.fcs	S-BrdU_high	1088	16.1161%
■ (red)	BA2026-03-10_Alisertib_R2.0001.fcs	S-BrdU-low	601	8.9024%
■ (blue)	BA2026-03-10_Alisertib_R2.0001.fcs	G2-M	3979	58.9394%
