# Supplementary material for: AURORA A interacts with DICER and SETD2 to promote S-phase progression
Source: EMBO Rep. 2026 Jun 30;27(15):4493–523. doi: 10.1038/s44319-026-00850-0 (PMC13458143; doi:10.1038/s44319-026-00850-0)
Supplement: Supplementary file 5 [file 44319_2026_850_MOESM7_ESM.zip › Figure 1/1I/1I_WB_overview.pdf]

1I

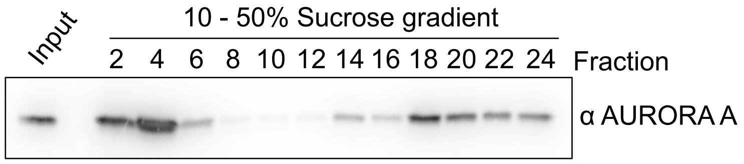
Input
10 - 50% Sucrose gradient
2 4 6 8 10 12 14 16 18 20 22 24 Fraction
α AURORA A

Raw data:

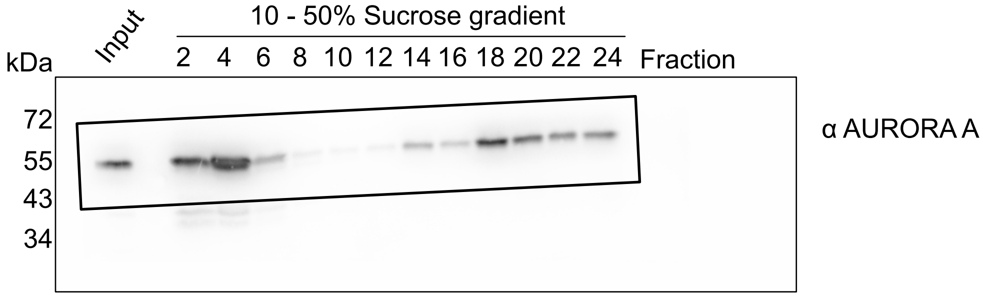
Input
10 - 50% Sucrose gradient
kDa
2 4 6 8 10 12 14 16 18 20 22 24 Fraction
72
55
43
34
α AURORA A
