# Supplementary material for: AURORA A interacts with DICER and SETD2 to promote S-phase progression
Source: EMBO Rep. 2026 Jun 30;27(15):4493–523. doi: 10.1038/s44319-026-00850-0 (PMC13458143; doi:10.1038/s44319-026-00850-0)

3F DICER IP

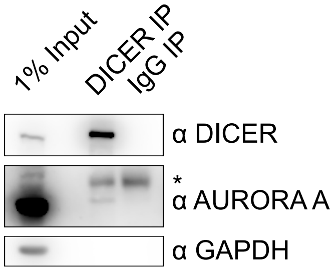

Raw data:

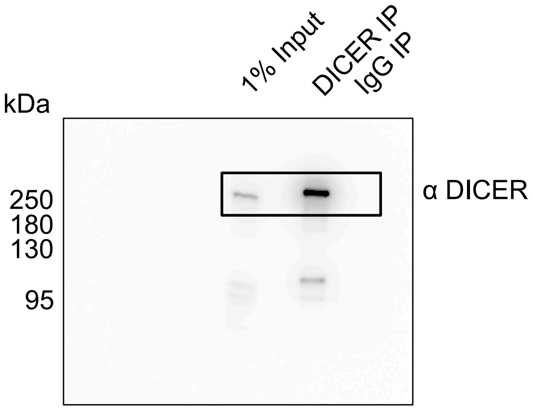

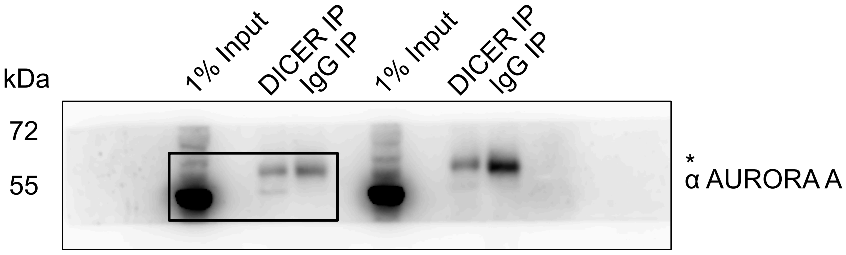

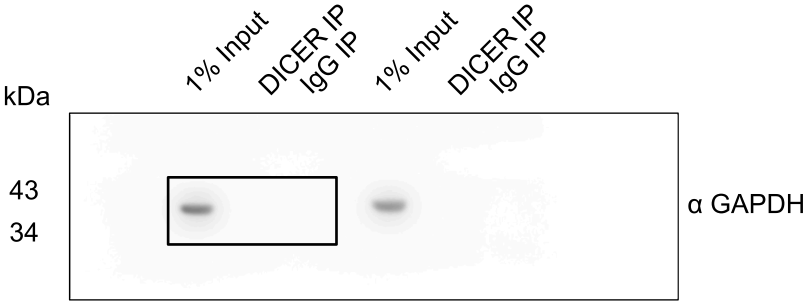

Supplement: Supplementary file 7 [file 44319_2026_850_MOESM9_ESM.zip › Figure 3/3F/3F_WB_overview.pdf]
