# Supplementary material for: AURORA A interacts with DICER and SETD2 to promote S-phase progression
Source: EMBO Rep. 2026 Jun 30;27(15):4493–523. doi: 10.1038/s44319-026-00850-0 (PMC13458143; doi:10.1038/s44319-026-00850-0)
Supplement: Supplementary file 7 [file 44319_2026_850_MOESM9_ESM.zip › Figure 3/3H/3H_WB_overview.pdf]

3H

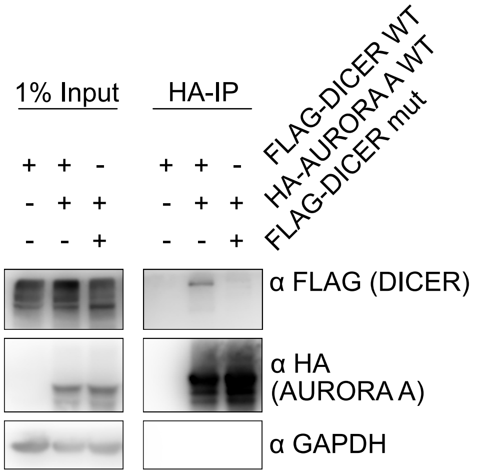
1% Input
HA-IP
FLAG-DICER WT
HA-AURORA A WT
FLAG-DICER mut
+ + - + + -
- + + - + +
- - + - - +
α FLAG (DICER)
α HA
(AURORA A)
α GAPDH

Raw data:

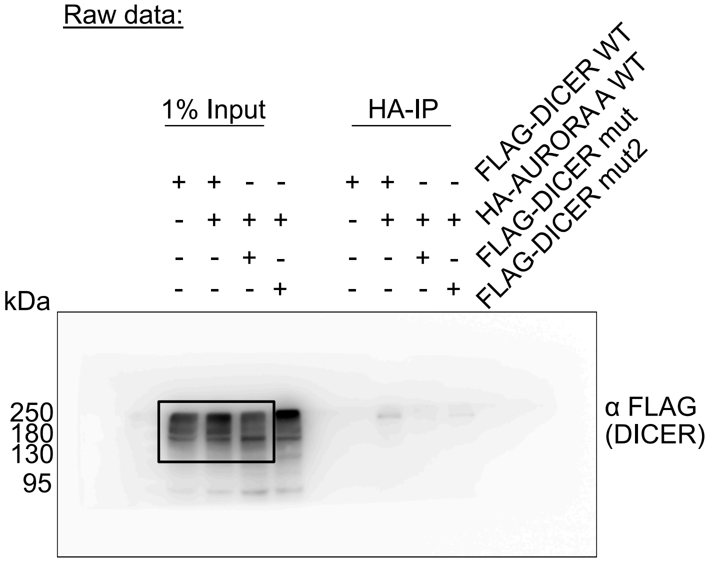
1% Input
HA-IP
FLAG-DICER WT
HA-AURORA A WT
FLAG-DICER mut
FLAG-DICER mut2
+ + - - + + - -
- + + + - + + +
- - + - - - + -
- - - + - - - +
kDa
250
180
130
95
α FLAG
(DICER)

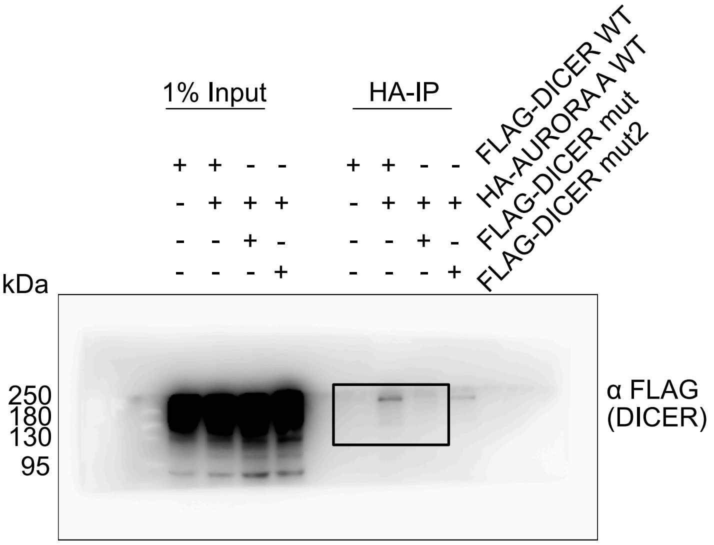
1% Input
HA-IP
FLAG-DICER WT
HA-AURORA A WT
FLAG-DICER mut
FLAG-DICER mut2
+ + - - + + - -
- + + + - + + +
- - + - - - + -
- - - + - - - +
kDa
250
180
130
95
α FLAG
(DICER)

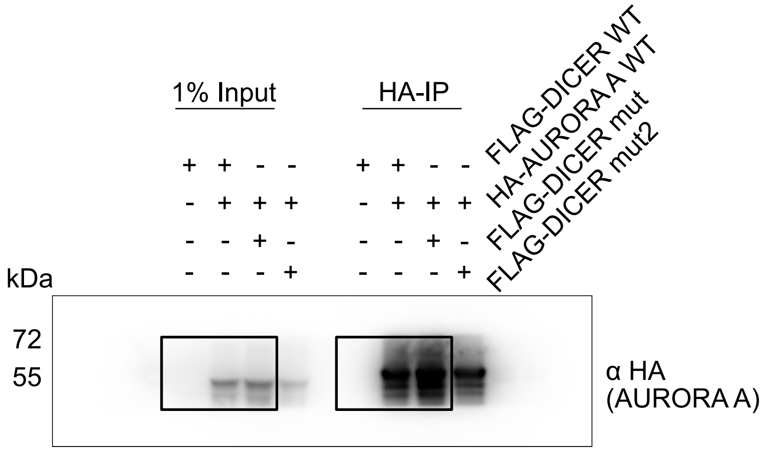
1% Input
HA-IP
FLAG-DICER WT
HA-AURORA A WT
FLAG-DICER mut
FLAG-DICER mut2
+ + - - + + - -
- + + + - + + +
- - + - - - + -
- - - + - - - +
kDa
72
55
α HA
(AURORA A)

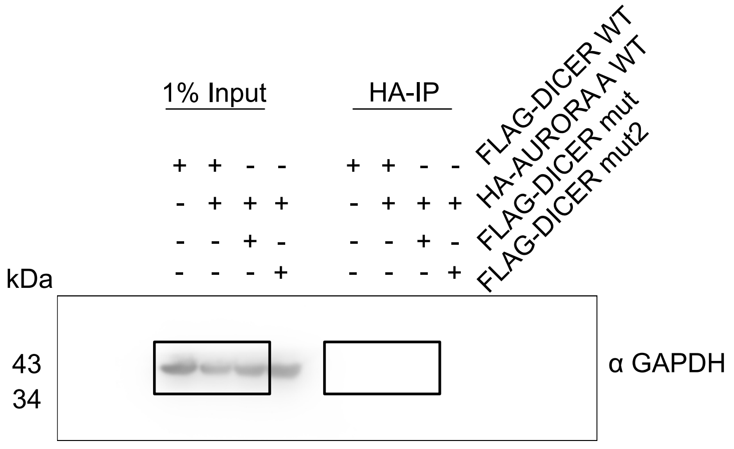
1% Input
HA-IP
FLAG-DICER WT
HA-AURORA A WT
FLAG-DICER mut
FLAG-DICER mut2
+ + - - + + - -
- + + + - + + +
- - + - - - + -
- - - + - - - +
kDa
43
34
α GAPDH
